# Szegő Kernel Asymptotics on Complete Strictly Pseudoconvex CR Manifolds

**DOI:** 10.1007/s12220-022-00990-4

**Published:** 2022-08-18

**Authors:** Chin-Yu Hsiao, George Marinescu, Huan Wang

**Affiliations:** 1 Institute of Mathematics, Academia Sinica, Taipei, Taiwan; 2 Mathematisches Institut, Universität zu Köln, Weyertal 86-90, 50931 Köln, Germany; 3 Institute of Mathematics ‘Simion Stoilow’, Romanian Academy, Bucharest, Romania; 4 Tong Jing Nan Lu 798, Suzhou, People’s Republic of China

**Keywords:** CR manifolds, Kohn Laplacian, Szegő kernel, Bochner–Kodaira–Nakano formula, Primary: 32V20, 32V30, Secondary: 32W10, 32L20

## Abstract

We prove a Bochner–Kodaira–Nakano formula and establish Szegő kernel expansions on complete strictly pseudoconvex CR manifolds with transversal CR $\mathbb {R}$-action under certain natural geometric conditions. As a consequence we show that such manifolds are locally CR embeddable.

## Introduction

The first goal of this paper is to develop a differential geometric formalism on strictly pseudoconvex CR manifolds with $\mathbb {R}$-action, analogous to the Kähler identities and Bochner–Kodaira–Nakano formula for Hermitian manifolds. We refine in this way Tanaka’s formulas in the spirit of Demailly’s general version of the latter formulas. This formalism leads to vanishing theorems and $L^2$-estimates for the $\overline{\partial }_b$-operator for complete CR manifolds.

The second goal is to generalize the result of Boutet de Monvel–Sjöstrand about the singularities of the Szegő kernel for complete strictly pseudoconvex CR manifolds with $\mathbb {R}$-action. This entails global and local embeddability theorems for CR manifolds with $\mathbb {R}$-action, including Sasakian manifolds. Moreover, by applying our result for the Grauert tube of a positive line bundle we obtain a new result about the expansion of the Bergman kernel on complete Kähler manifolds.

Let $(X, T^{(1,0)}X)$ be a CR manifold of dimension $2n+1$, $n\ge 1$. The orthogonal projection $S^{(q)}:L^2_{0,q}(X)\rightarrow \ker \Box ^{(q)}_b$ onto $\ker \Box ^{(q)}_b$ is called the Szegő projection, while its distribution kernel $S^{(q)}(x,y)$ is called the Szegő kernel, where $\Box ^{(q)}_b$ denotes the Kohn Laplacian acting on (0, *q*)-forms. The study of the Szegő kernel is a classical subject in several complex variables and CR geometry. If *X* is compact strictly pseudoconvex and $\Box ^{(0)}_b$ has closed range, Boutet de Monvel–Sjöstrand [5] showed that $S^{(0)}(x,y)$ is a complex Fourier integral operator. The Boutet de Monvel–Sjöstrand description of the Szegő kernel had a profound impact in several complex variables, symplectic and contact geometry, geometric quantization, and Kähler geometry. These ideas also partly motivated the introduction of the recent direct approaches and their various extensions, see [23, 24].

However, almost all the results on Szegő kernel assumed that *X* is compact, while for non-compact complex manifolds the Bergman kernel asymptotics was comprehensively studied [18, 19, 23–25], and used in the several applications mentioned above. Note that for CR manifolds, besides the global embeddability question [4, 26], there is an important delicate specific issue, namely the local embeddability [1, 20, 22, 27], which will be treated here by the analysis of the Szegő kernel.

The Szegő kernel was used by Boutet de Monvel–Guillemin [6] to introduce the Toeplitz quantization on compact contact manifolds. In the same vein, the question of “quantization commutes with reduction” was studied on CR manifolds in the recent paper [17]. It is natural to extend these results to complete Sasakian manifolds.

Let us see some simple examples. Consider the hypersurface $Y:=\{z=(z_1,\ldots ,z_n)\in {\mathbb {C}}^n;\, \mathrm{Im\,}z_n=f(z_1,\ldots ,z_{n-1})\}$, where $f\in \mathscr {C}^\infty ({\mathbb {C}}^{n-1},{\mathbb {R}})$. Then *Y* is a non-compact CR manifold carrying many smooth CR functions, but even in this simple example we do not know the behavior of the associated Szegő kernel. Another example is the Heisenberg manifold $\mathbb {H}=\mathbb {C}^n\times \mathbb {R}$ with CR structure $T^{(1,0)}\mathbb {H}:=\mathrm{span\,}\left\{ \frac{\partial }{\partial z_j}+ i\frac{\partial \phi }{\partial z_j}(z)\frac{\partial }{\partial x_{2n+1}}:1\le j\le n\right\}$, where $\phi \in \mathscr {C}^\infty (\mathbb {C}^n,\mathbb {R})$. Then, $\mathbb {H}$ is also a non-compact CR manifold and the Szegő kernel has been studied when $\phi$ is quadratic (see [14]). However, for general $\phi$ there are fewer results. Both *Y* and $\mathbb {H}$ are non-compact CR manifolds with transversal CR ${\mathbb {R}}$-action. Therefore, we think that the study of the Szegő kernels on non-compact CR manifolds with transversal CR ${\mathbb {R}}$-action is a very natural and interesting question.

In [15], the first author obtained the Szegő kernel asymptotic expansion on the non-degenerate part of the Levi form under the assumption that Kohn Laplacian has closed range in $L^2$. The method in [15] works well for non-compact setting, but for general non-compact CR manifolds, the closed range property is not a natural assumption. In the Heisenberg case mentioned above, even for $\phi$ quadratic, $\Box ^{(0)}_b$ does not have closed range; however, the Szegő kernel still has an asymptotic expansion.

In this paper, we show that $\Box ^{(0)}_b$ has local closed range with respect to a spectral projection $Q_\lambda$ (see Definition 4.12) under certain geometric conditions. Furthermore, combining this local closed range property with a detailed analysis, we establish Szegő kernel asymptotic expansions on non-compact strictly pseudoconvex complete CR manifolds with transversal CR $\mathbb {R}$-action under certain natural geometric conditions. To study the local closed range property, we establish a CR Bochner–Kodaira–Nakano formula analog to [9], see Theorem 3.3, which has its own interest. This is also a refinement of Tanaka’s basic identities [28, Theorems 5.1, 5.2] in our context. We remark that the results in this paper hold both for transversal CR $\mathbb {R}$-action and $S^1$-action.

We will work in the following setting. Let *X* be a connected smooth paracompact manifold of dimension $2n+1$, *HX* be a smooth sub-bundle of *TX* of rank 2*n*, and *J* be a smooth complex structure on the fibers of *HX*. Let $T^{(1,0)}X$ be the complex sub-bundle of the complexification $\mathbb {C}HX$ of *HX*, which corresponds to the *i* eigenspace of *J*, that is, $T^{(1,0)}X=\{v-iJv:v\in HX\}$. We say that *X* is a CR manifold (of hypersurface type) if the formal integrability condition

1.1 $$\begin{aligned} \big [\mathscr {C}^\infty (X,T^{(1,0)}X),\mathscr {C}^\infty (X,T^{(1,0)}X)\big ] \subset \mathscr {C}^\infty (X,T^{(1,0)}X). \end{aligned}$$

holds. The sub-bundle *HX* is called Levi distribution and the annihilator $(HX)^0\subset T^*X$ of *HX* is called the characteristic bundle of the CR manifold *X*.

We will assume in the sequel that *X* is orientable. Since *HX* is oriented by its complex structure, it follows that $(HX)^0$ is a real orientable line bundle, thus trivial. A global frame of $(HX)^0$, that is, a real non-vanishing 1-form $\omega _0\in \mathscr {C}^\infty (X,T^*X)$ such that $(HX)^0=\mathbb {R}\omega _0$, is called characteristic 1-form.

Given a characteristic 1-form $\omega _0$ on *X* the Levi form $\mathscr {L}^{\omega _0}$ is defined by

1.2 $$\begin{aligned} \mathscr {L}=\mathscr {L}^{\omega _{0}}_x(u,v)=\frac{1}{2}d\omega _{0}(u,Jv), \quad \text { for } u, v\in H_xX. \end{aligned}$$

We say that (*X*, *HX*, *J*) is strictly pseudoconvex if there exists a characteristic 1-form $\omega _0$ the Levi form $\mathscr {L}^{\omega _0}_x$ is positive definite at every point $x\in X$. If $\mathscr {L}^{\omega _0}$ is positive definite, then $d\omega _0$ is symplectic on *HX*, thus $\omega _0$ is a contact form and *HX* is a contact structure. Associated with a contact form $\omega _0$ one has the Reeb vector field $T=T^{\omega _0}$, uniquely defined by the equations

1.3 $$\begin{aligned} \omega _0(T)=1,\quad d\omega _0(T,\cdot )=0\quad \text {on}\,X. \end{aligned}$$

### Assumption 1.1

$(X,HX,J,\omega _0)$ is an orientable strictly pseudoconvex CR manifold of dimension $2n+1$, $n\ge 1$, where *HX* is the Levi distribution, *J* is the complex structure, and $\omega _0$ is a contact form. We assume that *X* is endowed with a smooth locally free $\mathbb {R}$-action preserving $\omega _0$ and *J* such that the infinitesimal generator of the $\mathbb {R}$-action is a Reeb vector field, denoted *T*.

We denote by $T^{(1,0)}X$ and $T^{(0,1)}X$ the bundles of tangent vectors of type (1, 0) and (0, 1), respectively. By Assumption 1.1 the $\mathbb {R}$-action is CR and transversal (see (2.12) and (2.13)), hence we have a decomposition $\mathbb {C}TX = T^{(1,0)}X \oplus T^{(0,1)}X \oplus \mathbb {C}T$. The Levi form (1.2) induces a Hermitian metric, called the Levi (or Webster) metric,

1.4 $$\begin{aligned} \langle \,\cdot \,|\,\cdot \,\rangle _{\mathscr {L}}=g_{\mathscr {L}}:=\frac{1}{2}d\omega _0(\cdot ,J\cdot )+\omega _0(\cdot )\omega _0(\cdot ) \end{aligned}$$

on *TX* and by extension on $\mathbb {C}TX$, with the following properties:

1.5 $$\begin{aligned} T^{(1,0)}X\perp T^{(0,1)}X,\,\,T\perp (T^{(1,0)}X\oplus T^{(0,1)}X),\,\, \langle \,T\,|\,T\,\rangle _{\mathscr {L}}=1. \end{aligned}$$

In Sect. 2.2, we observe that Assumption 1.1 implies that the contact metric manifold $(X,\omega _0,T,J,g_{\mathscr {L}})$ is a Sasakian manifold. Conversely, every compact Sasakian manifold admits an $\mathbb {R}$-action as in Assumption 1.1.

Let $K^*_X:=\det (T^{(1,0)}X)$ and let $R^{K^*_X}_{\mathscr {L}}$ be the curvature of $K^*_X$ induced by $\langle \,\cdot \,|\,\cdot \,\rangle _{\mathscr {L}}$ (see (2.26) and (4.31)).

More generally, we consider an arbitrary $\mathbb {R}$-invariant Hermitian metric $g=g_X=\langle \,\cdot \,|\,\cdot \,\rangle _g= \langle \,\cdot \,|\,\cdot \,\rangle$ on $\mathbb {C}TX$ such that (1.5) holds. Given such a metric we will denote by $\Theta _X$ its fundamental (1, 1)-form given by $\Theta _X(a,\overline{b})=\sqrt{-1}\langle a\,|\,b\rangle _g$ for $a,b\in T^{(1,0)}X$. Let $dv_X$ be the volume form induced by the $\mathbb {R}$-invariant metric $g_X$ as in (1.5). Let $(\,\cdot \,|\,\cdot \,)$ be the $L^2$ inner product on the space of smooth compactly supported functions $\mathscr {C}_c^\infty (X)$ with respect to $dv_X$. We denote by $L^2(X,dv_X)$ the completion of $\mathscr {C}_c^\infty (X)$ with respect to $(\,\cdot \,|\,\cdot \,)$.

We denote by $\overline{\partial }_b$ the tangential Cauchy–Riemann operator (see Definition 2.3). The Szegő projection is the orthogonal projection with respect to $(\,\cdot \,|\,\cdot \,)$,

1.6 $$\begin{aligned} S^{(0)}: L^2(X,dv_X)\rightarrow \ker \overline{\partial }_b\cap L^2(X,dv_X), \end{aligned}$$

on the space of square-integrable CR functions on *X*. The distribution kernel $S^{(0)}(x,y)\in {\mathscr {D}}'(X\times X)$ of the Szegő projection is called the Szegő kernel. The main result of this article is as follows:

### Theorem 1.2

Let $(X,HX,J,\omega _0)$ be an orientable strictly pseudoconvex CR manifold of dimension $2n+1$, $n\ge 1$, with an $\mathbb {R}$-action on *X* as in Assumption 1.1. Let $g_X$ be an $\mathbb {R}$-invariant metric as in (1.5) and let $\Theta _X$ be its fundamental form. Assume that the Levi metric $g_\mathscr {L}$ is complete and there is $C>0$ such that

1.7 $$\begin{aligned} \sqrt{-1}R^{K^*_X}_{\mathscr {L}}\ge -2C\sqrt{-1}\mathscr {L}\,,\quad {(2\sqrt{-1}\mathscr {L})^n\wedge \omega _0\ge C\Theta _X^n\wedge \omega _0.} \end{aligned}$$

Then the Szegő projection is a Fourier integral operator with complex phase, that is, for any local coordinate patch $(D,x=(x_1,\ldots ,x_{2n+1}))$ with $D\Subset X$, we have

1.8 $$\begin{aligned} S^{(0)}(x,y)-\int ^\infty _0e^{i\varphi (x,y)t}s(x,y,t)dt\in \mathscr {C}^\infty (D\times D), \end{aligned}$$

where the phase function $\varphi \in \mathscr {C}^\infty (D\times D)$ satisfies

1.9 $$\begin{aligned} \begin{aligned}&\varphi \in \mathscr {C}^\infty (D\times D),\ \ \mathrm{Im\,}\varphi (x, y)\ge 0,\\&\varphi (x, x)=0,\ \ \varphi (x, y)\ne 0\ \ \text {if}\ \ x\ne y,\\&d_x\varphi (x, y)\big |_{x=y}=\omega _0(x), \ \ d_y\varphi (x, y)\big |_{x=y}=-\omega _0(x), \\&\varphi (x, y)=-\overline{\varphi }(y, x), \end{aligned} \end{aligned}$$

and $s(x, y, t)\in S^{n}_{\mathrm{cl\,}} \big (D\times D\times {\mathbb {R}}_+\big )$ is a symbol of order *n* with asymptotic expansion $s(x,y,t)=\sum _{j=0}^\infty s_j(x,y)t^{n-j}$ whose leading term $s_0(x,y)$ satisfies

1.10 $$\begin{aligned} s_0(x, x)=\frac{1}{2}\pi ^{-n-1} |\mathrm{det\,}{\mathscr {L}}_x|,\ \ \text {for all}\, x\in D, \end{aligned}$$

where $\det {\mathscr {L}}_x$ is the determinant of $\mathscr {L}_x$ with respect to $g_X$, cf. (4.73).

We will show in Lemma 2.7 that $R^{K^*_X}_{\mathscr {L}}={\text {Ric}}_{\mathscr {L}}$ where ${\text {Ric}}_{\mathscr {L}}\in \Omega ^{1,1}(X)$ is the pseudohermitian Ricci form with respect to the pseudohermitian structure $\omega _0$ (see (2.10)). We refer to Definition 2.1 for the definition of the symbol space $S^{n}_{\mathrm{cl\,}}\big (D\times D\times {\mathbb {R}}_+\big )$ and to [19, Theorems 3.3, 4.4] for more properties for the phase $\varphi$ in (1.8).

Examples for the situation described in Theorem 1.2 are given by Galois coverings of compact strictly pseudoconvex CR manifolds (Examples 4.5, 5.3), circle bundles of positive line bundles over complete Kähler manifolds (Example 4.6), and, as mentioned before, the Heisenberg group (Sect. 5).

If we work with (*n*, 0)-forms we can drop some of the hypotheses of Theorem 1.2.

### Theorem 1.3

Let $(X,HX,J,\omega _0)$ be an orientable strictly pseudoconvex CR manifold of dimension $2n+1$, $n\ge 1$, with an $\mathbb {R}$-action on *X* as in Assumption 1.1. Assume that the Levi metric $g_{\mathscr {L}}$ is complete. Then the Szegő projection $S^{(n,0)}:L^2_{n,0}(X)\rightarrow \ker \overline{\partial }_b \subset L^2_{n,0}(X)$ is a Fourier integral operator with complex phase, that is, for any local coordinate patch $(D,x=(x_1,\ldots ,x_{2n+1}))$ with $D\Subset X$, the Szegő kernel has the form (1.8) with respect to the trivialization of $K_X$ given by $dz_1\wedge \ldots \wedge dz_n$.

The equivariant Kodaira embedding theorems for Sasakian manifolds were obtained in [13, 16]. From Theorem 1.2, we obtain a Boutet de Monvel type embedding theorem [4] for complete Sasakian manifolds as follows, which is a generalization of the embedding theorem for compact Sasakian manifolds [26].

### Corollary 1.4

In the situation of Theorem 1.2 the space of $L^2$ CR functions separate points and give local coordinates on *X*. In particular, for any compact set of $K\subset X$ there exists a positive integer *N* and CR functions $f_1,\ldots ,f_N\in L^2(X)\cap \mathscr {C}^\infty (X)$ such that $(f_1,\ldots ,f_N)$ is an embedding of *K* in $\mathbb {C}^N$.

As a consequence of Theorem 1.3 we obtain the following:

### Corollary 1.5

In the situation of Theorem 1.3 the space of $L^2$ CR (*n*, 0)-forms separate points and give local coordinates on *X*. Thus, *X* is locally CR embeddable in an Euclidean space. In particular, every Sasakian manifold with complete Levi metric $g_{\mathscr {L}}$ is locally CR embeddable by global CR (*n*, 0)-forms.

The question arises if one can extend these results for general strictly pseudoconvex CR manifolds (without Assumption 1.1 about the existence of an $\mathbb {R}$-action). An analytic property that we use is that the spectral projections $Q_\lambda$ of the operator $\sqrt{-1}T$ (see 4.7) commute to $\overline{\partial }_b$. Beyond that it is not clear what would be general geometric or analytic conditions that would imply that the Szegő projector is a Fourier integral operator.

We now apply our main result to complex manifolds. Let $(L,h^L)$ be a holomorphic line bundle over a Hermitian manifold $(M,\Theta _M)$, where $h^L$ denotes a Hermitian metric on *L* and $\Theta _M$ is a positive (1, 1) form on *M*. For every $k\in {\mathbb {N}}$, let $(L^k,h^{L^k})$ be the *k*-th power of $(L,h^L)$. The positive (1, 1) form $\Theta _M$ and $h^{L^k}$ induces a $L^2$ inner product $(\,\cdot \,|\,\cdot \,)_{\Theta _M}$ on $\Omega ^{0,q}_c(M,L^k))$. Let $L^2_{0,q}(M,L^k)$ be the completion of $\Omega ^{0,q}_c(M,L^k)$ with respect to $(\,\cdot \,|\,\cdot \,)_{\Theta _M}$. We write $L^2(M,L^k):=L^2_{0,0}(M,L^k)$. Let

$$\begin{aligned} H^0_{(2)}(M,L^k)=\ker \overline{\partial }_k:=\{u\in L^2(M,L^k);\, \overline{\partial }u=0\}, \end{aligned}$$

be the space of holomorphic square-integrable sections of $L^k$. Let $\{f_j^k\}_{j=1}^{{d_k}}$ be an orthonormal basis for $H^0_{(2)}(M,L^k)$ with respect to $(\,\cdot \,|\,\cdot \,)_{\Theta _M}$, where $d_k\in {\mathbb {N}}\cup \{\infty \}$. The Bergman kernel of $L^k$ is

1.11 $$\begin{aligned} P_{k}(x,y):=\sum ^{d_k}_{j=1}f_j^k(x)\otimes f_j^k(y)^*\in \mathscr {C}^\infty (X\times X, L^k\boxtimes (L^{k})^*). \end{aligned}$$

Let *s* be a local holomorphic frame of *L* defined on an open set $D\Subset M$, $|s|^2_{h^L}=e^{-2\phi }$, $\phi \in \mathscr {C}^\infty (D,{\mathbb {R}})$. On *D*, we write $f_j^k={\tilde{f}}_j^ks^{\otimes k}$, ${\tilde{f}}_j^k\in \mathscr {C}^\infty (D)$, $j=1,\ldots ,d_k$. The localized Bergman kernel on *D* is given by

1.12 $$\begin{aligned} P_{k,s}(x,y):=\sum ^{d_k}_{j=1}e^{-k\phi (x)} {\tilde{f}}_j^k(x)\overline{{\tilde{f}}_j^k(y)}e^{-k\phi (y)}\in \mathscr {C}^\infty (D\times D). \end{aligned}$$

Let $R^L$ be the Chern curvature of *L* induced by $h^L$. Assume that $\omega =\sqrt{-1}R^L$ is positive. Let $K^*_M:=\det (T^{(1,0)}M)$ and let $R^{K^*_M}_{\omega }$ be the curvature of $K^*_M$ induced by $\omega$. Applying Theorem 1.2 to the circle bundle of $(L,h^L)$, we get the following:

### Theorem 1.6

Let $(L,h^L)$ be a Hermitian holomorphic line bundle over a Hermitian manifold $(M,\Theta _M)$ of dimension *n*. We assume that $\omega =\sqrt{-1}R^L$ defines a complete Kähler metric on *M*. We assume moreover that there is $C>0$ such that

1.13 $$\begin{aligned} \sqrt{-1}R^{K^*_M}_{\omega }\ge -C\omega ,\quad \omega ^n\ge C\Theta _M^n \quad \text {on}\, M. \end{aligned}$$

Let *s* be a local holomorphic frame of *L* defined on an open set $D\Subset M$. Then,

1.14 $$\begin{aligned} P_{k,s}(x,y)\equiv e^{ik\Phi (x,y)}b(x,y,k)\mod O(k^{-\infty })\ \ \text {on}\, D, \end{aligned}$$

where $\Phi \in \mathscr {C}^\infty (D\times D)$, $\mathrm{Im\,}\Phi (x, y)\ge C|x-y|^2$, $C>0$, $\Phi (x,x)=0$, for every $x\in D$,

1.15 $$\begin{aligned} b(x,y,k)\in S^n_{\mathrm{loc\,}}(1;D\times D),\ \ b(x,y,k)\sim \sum ^{\infty }_{j=0}k^{n-j}b_j(x,y)\ \ \text {in}\, S^n_{\mathrm{loc\,}}(1;D\times D), \end{aligned}$$

$$b_j(x,y)\in \mathscr {C}^\infty (D\times D)$$, $$j=0,1,\ldots $$ , and

$$\begin{aligned} b_0(x,x)=(2\pi )^{-n}\frac{\omega ^n(x)}{\Theta ^n_M(x)}\,,\ \ \text {for every}\, x\in D. \end{aligned}$$

In particular, there exist coefficients $\varvec{b}_r\in \mathscr {C}^\infty (X)$, $r\in \mathbb {N}_0$, such that for any open set *U* of *X* with ${\overline{U}}$ compact, every $\ell \in {\mathbb {N}}_0$ and every $m\in {\mathbb {N}}$, there is a $C_{U,\ell ,m}>0$ independent of *k* such that

1.16 $$\begin{aligned} \Vert P_k(x,x)-\sum _{r=0}^m\varvec{b}_r(x)k^{n-r}\Vert _{\mathscr {C}^\ell (U)}\le C_{U,\ell ,m}k^{n-m-1}. \end{aligned}$$

We refer the reader to Sect. 2 for the precise meaning of the notation $A_k\equiv B_k\mod O(k^{-\infty })$ on *D* in (1.14), $S^n_{\mathrm{loc\,}}(1;D\times D)$ and the asymptotic sums in (1.15) and (1.16).

For compact or certain complete Kähler–Einstein manifolds, the expansion (1.16) was obtained by Tian [29] for $m=0$ and $\ell =4$. For general *m*, $\ell$, and compact manifolds, the existence of the expansion was first obtained in [8, 31]. In [23, Theorem 6.1.1] the expansion was generalized for complete Hermitian manifolds such that $R^{K^*_M}$ and $\partial \Theta _M$ are bounded below. Our conditions (1.13) are different from [23, Theorem 6.1.1], we replace the condition on $\partial \Theta _M$ by a condition on the volume form. The reason is that we use a local closed range condition instead of standard closed range or spectral gap condition.

This paper is organized as follows. In Sect. 2, we recall necessary notions of microlocal analysis, pseudohermitian geometry, and strictly pseudoconvex CR manifolds with transversal CR $\mathbb {R}$-actions. In Sect. 3, we prove the Bochner–Kodaira formula on CR manifolds with $\mathbb {R}$-action. Section 4 is devoted to the proof of the asymptotics of the Szegő kernel. In Sect. 5, we examine the Heisenberg group.

## Preliminaries

We use the following notations through this article: ${\mathbb {N}}=\{1,2,\ldots \}$ is the set of natural numbers, ${\mathbb {N}}_0={\mathbb {N}}\bigcup \{0\}$, ${\mathbb {R}}$ is the set of real numbers, $\overline{{\mathbb {R}}}_+=\{x\in {\mathbb {R}};\, x\ge 0\}$. For $m\in \mathbb {N}$, let $x=(x_1,\ldots ,x_m)$ be coordinates of $\mathbb {R}^m$. For $n\in \mathbb {N}$, let $z=(z_1,\ldots ,z_n)$, $z_j=x_{2j-1}+\sqrt{-1}x_{2j}$, $j=1,\ldots ,n$, be coordinates of $\mathbb {C}^n$. We write

2.1 $$\begin{aligned}&\frac{\partial }{\partial z_j}:=\frac{1}{2}\left( \frac{\partial }{\partial x_{2j-1}}-\sqrt{-1}\frac{\partial }{\partial x_{2j}}\right) , \quad \frac{\partial }{\partial \overline{z}_j}:=\frac{1}{2}\left( \frac{\partial }{\partial x_{2j-1}} +\sqrt{-1}\frac{\partial }{\partial x_{2j}}\right) ,\quad \quad \end{aligned}$$

2.2 $$\begin{aligned}&dz_j=dx_{2j-1}+\sqrt{-1}dx_{2j}, \quad d\overline{z}_j=dx_{2j-1}-\sqrt{-1}dx_{2j}. \end{aligned}$$

### Notions of Microlocal Analysis

Let *X* be a $\mathscr {C}^\infty$ paracompact manifold. We let *TX* and $T^*X$ denote the tangent bundle of *X* and the cotangent bundle of *X*, respectively. The complexified tangent bundle of *X* and the complexified cotangent bundle of *X* are denoted by $\mathbb CTX$ and ${\mathbb {C}} T^*X$, respectively. Write $\langle \,\cdot \,,\cdot \,\rangle$ to denote the pointwise duality between *TX* and $T^*X$. We extend $\langle \,\cdot \,,\cdot \,\rangle$ bilinearly to $\mathbb CTX\times {\mathbb {C}} T^*X$.

Let $D\subset X$ be an open set . The spaces of distributions of *D* and smooth functions of *D* will be denoted by ${\mathscr {D}}'(D)$ and $\mathscr {C}^\infty (D)$, respectively. Let ${\mathscr {E}}'(D)$ be the subspace of ${\mathscr {D}}'(D)$ whose elements have compact support in *D*. Let $\mathscr {C}^\infty _c(D)$ be the subspace of $\mathscr {C}^\infty (D)$ whose elements have compact support in *D*. Let $A: \mathscr {C}^\infty _c(D)\rightarrow {\mathscr {D}}'(D)$ be a continuous map. We write *A*(*x*, *y*) to denote the distribution kernel of *A*. In this work, we will identify *A* with *A*(*x*, *y*). The following two statements are equivalent:

(I) *A* is continuous: $${\mathscr {E}}'(D)\rightarrow \mathscr {C}^\infty (D)$$,
(II) $$A(x,y)\in \mathscr {C}^\infty (D\times D)$$.

If *A* satisfies (I) or (II), we say that *A* is smoothing on *D*. Let $A,B: \mathscr {C}^\infty _c(D)\rightarrow {\mathscr {D}}'(D)$ be continuous operators. We write

2.3 $$\begin{aligned} A\equiv B (\text {on}\, D) \end{aligned}$$

if $A-B$ is a smoothing operator. We say that *A* is properly supported if the restrictions of the two projections $(x,y)\rightarrow x$, $(x,y)\rightarrow y$ to $\mathrm{Supp\,}(A(x,y))$ are proper.

For $m\in {\mathbb {R}}$, let $H^m(D)$ denote the Sobolev space of order *m* on *D*. Put

$$\begin{aligned}&\quad H^m_\mathrm{loc\,}(D)=\big \{u\in {\mathscr {D}}'(D);\, \varphi u\in H^m(D), \, \forall \varphi \in \mathscr {C}^\infty _c(D)\big \}\,,\\&H^m_\mathrm{comp\,}(D)=H^m_\mathrm{loc}(D)\cap {\mathscr {E}}'(D)\,. \end{aligned}$$

Let *D* be an open coordinate patch of *X* with local coordinates *x*. We recall the following Hörmander symbol space.

#### Definition 2.1

For $m\in {\mathbb {R}}$, $S^m_{1,0}(D\times D\times {\mathbb {R}}_+)$ is the space of all $a(x,y,t)\in \mathscr {C}^\infty (D\times D\times {\mathbb {R}}_+)$ such that for all compact $K\Subset D\times D$ and all $\alpha , \beta \in {\mathbb {N}}^{2n+1}_0$, $\gamma \in {\mathbb {N}}_0$, there is a constant $C_{\alpha ,\beta ,\gamma }>0$ such that

$$\begin{aligned} |\partial ^\alpha _x\partial ^\beta _y\partial ^\gamma _t a(x,y,t)|\le C_{\alpha ,\beta ,\gamma }(1+|t|)^{m-|\gamma |},\ \ \text {for all}\, (x,y,t)\in K\times {\mathbb {R}}_+, t\ge 1. \end{aligned}$$

Put

$$\begin{aligned} S^{-\infty }(D\times D\times {\mathbb {R}}_+) :=\bigcap _{m\in {\mathbb {R}}}S^m_{1,0}(D\times D\times {\mathbb {R}}_+). \end{aligned}$$

Let $a_j\in S^{m_j}_{1,0}(D\times D\times {\mathbb {R}}_+)$, $j\in \mathbb {N}_0$, with $m_j\searrow -\infty$, $j\rightarrow \infty$. Then there exists $a\in S^{m_0}_{1,0}(D\times D\times {\mathbb {R}}_+)$, unique modulo $S^{-\infty }$, such that $a-\sum ^{k-1}_{j=0}a_j\in S^{m_k}_{1,0}(D\times D\times {\mathbb {R}}_+)$ for $k\in \mathbb {N}$. If *a* and $a_j$ have the properties above, we write

$$\begin{aligned} a\sim \sum ^{\infty }_{j=0}a_j\,\, \text {in}\, S^{m_0}_{1,0}(D\times D\times {\mathbb {R}}_+). \end{aligned}$$

The space $S^{m}_{\mathrm{cl\,}}(D\times D\times {\mathbb {R}}_+)$ of classical symbols of order *m* is defined as the space of symbols $s(x,y,t)\in S^{m}(D\times D\times {\mathbb {R}}_+)$ satisfying

2.4 $$\begin{aligned} \begin{aligned}&s(x, y, t)\sim \sum ^\infty _{j=0}s_j(x, y)t^{m-j}\text { in }\,S^{m}_{1, 0} (D\times D\times {\mathbb {R}}_+)\,,\\&s_j(x, y)\in \mathscr {C}^\infty (D\times D),\ j\in \mathbb {N}_0. \end{aligned} \end{aligned}$$

We explain now for the precise meaning of $A_k\equiv B_k\mod O(k^{-\infty })$ on *D* in (1.14), $S^n_{\mathrm{loc\,}}(1;D\times D)$ and the asymptotic sum in (1.15) (see also [18, Sect. 3.3]). A *k*-dependent smoothing operator $A_k:\Omega ^{0,q}_0(D)\rightarrow \Omega ^{0,q}(D)$ is called *k*-negligible if the kernel $A_k(x, y)$ of $A_k$ satisfies $|\partial ^\alpha _x\partial ^\beta _yA_k(x, y)|=O(k^{-N})$ uniformly on every compact set in $D\times D$, for all multi-indices $\alpha$, $\beta$, and all $N\in {\mathbb {N}}$. Let $C_k:\Omega ^{0,q}_0(D)\rightarrow \Omega ^{0,q}(D)$ be another *k*-dependent smoothing operator. We write $A_k\equiv C_k\mod O(k^{-\infty })$ or $A_k(x,y)\equiv C_k(x,y)\mod O(k^{-\infty })$ if $A_k-C_k$ is *k*-negligible.

We recall the definition of semi-classical Hörmander symbol spaces:

#### Definition 2.2

Let *U* be an open set in $\mathbb {R}^N$. Let $S(1;U)=S(1)$ be the set of $a\in \mathscr {C}^\infty (U)$ such that for every $\alpha \in {\mathbb {N}}^N_0$, there exists $C_\alpha >0$, such that $|\partial ^\alpha _xa(x)|\le C_\alpha$ on *U*. If $a=a(x,k)$ depends on $k\in (1,\infty )$, we say that $a(x,k)\in S_{\mathrm{loc\,}}(1)$ if $\chi (x)a(x,k)$ is uniformly bounded in *S*(1) when *k* varies in $(1,\infty )$, for any $\chi \in \mathscr {C}^\infty _0(U)$. For $m\in \mathbb {R}$, we put $S^m_{\mathrm{loc}}(1)=k^mS_{\mathrm{loc\,}}(1)$. If $a_j\in S^{m_j}_{\mathrm{loc\,}}(1)$, $m_j\searrow -\infty$, we say that $a\sim \sum ^\infty _{j=0}a_j$ in $S^{m_0}_{\mathrm{loc\,}}(1)$ if $a-\sum ^{N_0}_{j=0}a_j\in S^{m_{N_0+1}}_{\mathrm{loc\,}}(1)$ for every $N_0$. From this, we form $S^m_{\mathrm{loc\,}}(1;Y, E)$ in the natural way, where *Y* is a smooth paracompact manifold and *E* is a vector bundle over *Y*.

Let *X* be an orientable paracompact smooth manifold of dimension $2n+1$ with $n\ge 1$. The Levi form (1.2) of *X* at $x\in X$ induces a Hermitian quadratic form on $T^{(1,0)}_xX$ by

2.5 $$\begin{aligned} \mathscr {L}_x(u,\overline{v})=\frac{1}{2i} d\omega _{0}(u, \overline{v}) , \quad \text { for }\, u, v\in T^{(1,0)}_xX. \end{aligned}$$

Let $g^{\mathbb {C}TX}$ be a Hermitian metric on $\mathbb {C}TX$ such that the decomposition $\mathbb {C}TX = T^{(1,0)}X \oplus T^{(0,1)}X \oplus \mathbb {C}T$ is orthogonal. For $u,v\in \mathbb {C}TX$ we denote by $\langle u|v\rangle =\langle u|v\rangle _g$ the inner product given by $g^{\mathbb {C}TX}$ and for $u \in \mathbb {C}TX$, we write $|u|^2_g := \langle u| u \rangle _g$. Given such a metric we will denote by $\Theta _X$ its fundamental (1, 1)-form given by $\Theta _X(a,\overline{b})=\sqrt{-1}\langle a\,|\,b\rangle _g$ for $a,b\in T^{(1,0)}X$.

For $p, q\in {\mathbb {N}}_0$, define $T^{(p,q)}X :=(\Lambda ^pT^{(1,0)}X)\wedge (\Lambda ^qT^{(0,1)}X)$ and let $T^{\bullet ,\bullet }X=\bigoplus _{p,q\in \mathbb {N}_0}T^{(p,q)}X$. For $u\in \mathbb {C}TX$ and $\phi \in \mathbb {C}T^*X$, the pointwise duality is defined by $\langle u,\phi \rangle :=\phi (u).$ Let $T^{*(1,0)}X\subset \mathbb {C}T^*X$ be the dual bundle of $T^{(1,0)}X$ and $T^{*(0,1)}X\subset \mathbb {C}T^*X$ be the dual bundle of $T^{(0,1)}X$. For $p, q\in {\mathbb {N}}_0$, the bundle of (*p*, *q*) forms is denoted by $T^{*(p,q)}X:=(\Lambda ^pT^{*(1,0)}X)\wedge (\Lambda ^qT^{*(0,1)}X)$ and let $T^{*\bullet ,\bullet }X:=\oplus _{p,q\in {\mathbb {N}}_0}T^{*(p,q)}X$. The induced Hermitian inner product on $T^{\bullet ,\bullet }X$ and $T^{*\bullet ,\bullet }X$ by $\langle \,\cdot \,|\,\cdot \,\rangle$ are still denoted by $\langle \cdot |\cdot \rangle$. The Hermitian norms are still denoted by $|\cdot |$. Let $\Omega ^{p,q}(X):=\mathscr {C}^{\infty }(X,T^{*(p,q)}X)$ be the space of smooth (*p*, *q*)-forms on *X* and $\Omega ^{\bullet ,\bullet }(X):=\bigoplus _{p,q\in \mathbb {N}_0}\Omega ^{p,q}(X)$. Let $\mathscr {C}^\infty (X):=\Omega ^{0,0}(X)$.

#### Definition 2.3

Let $\pi ^{p,q}: \Lambda ^{p+q} \mathbb {C}T^*X\longrightarrow T^{*(p,q)}X$ be the natural projection for $p,q\in \mathbb {N}_0$, $p+q\ge 1$. The tangential (resp. anti-tangential) Cauchy–Riemann operator is given by

2.6 $$\begin{aligned} \overline{\partial }_b:= & {} \pi ^{p,q+1}\circ d: \Omega ^{p,q}(X) \longrightarrow \Omega ^{p,q+1}(X), \nonumber \\&\quad \partial _b:=\pi ^{p+1,q}\circ d: \Omega ^{p,q}(X) \longrightarrow \Omega ^{p+1,q}(X). \end{aligned}$$

Let $D\subset X$ be an open set. Let $\Omega ^{p,q}_c(D)$ be the space of smooth (*p*, *q*)-forms on *D* with compact support in *D*. Let $\Omega ^{\bullet ,\bullet }_c(D):= \bigoplus _{p,q\in \mathbb {N}_0}\Omega ^{p,q}_c(D)$. We write $\mathscr {C}^\infty _c(D):=\Omega ^{0,0}_c(D)$. Let $(\,\cdot \,|\,\cdot \,)$ be the $L^2$ inner product on $\Omega ^{\bullet ,\bullet }_c(X)$ induced by $\langle \,\cdot \,|\,\cdot \,\rangle$. Note that

2.7 $$\begin{aligned} (u|v):=\int _X\langle u(x)|v(x) \rangle dv_X(x),\ \ u, v\in \Omega ^{\bullet ,\bullet }_c(X), \end{aligned}$$

where $dv_X:=(\Theta _X^n/n!)\wedge \omega _0$ is the volume form induced by the Hermitian metric $\Theta _X$ on *X*. Let $L^2_{p,q}(X)$ be the completion of $\Omega ^{p,q}_c(X)$ with respect to $(\,\cdot \,|\,\cdot \,)$. Let $L^2_{\bullet ,\bullet }(X):=\bigoplus _{p,q\in \mathbb {N}_0} L^2_{p,q}(X)$. We write $L^2(X):=L^2_{0,0}(X)$. We denote by $\Vert u\Vert ^2:=(u|u)$ the $L^2$-norm on *X*. Let $\overline{\partial }_b^*$ and $\partial _b^*$ be the formal adjoints of $\overline{\partial }_b$ and $\partial _b$ with respect to $(\,\cdot \,|\,\cdot \,)$, respectively. Let $\square _b:=\overline{\partial }_b\overline{\partial }_b^*+\overline{\partial }_b^* \overline{\partial }_b$ be the Kohn Laplacian on $\Omega ^{\bullet ,\bullet }(X)$. Let $\overline{\square }_b:=\partial _b\partial _b^*+\partial _b^* \partial _b$ be the anti-Kohn Laplacian on $\Omega ^{\bullet ,\bullet }(X)$. We still denoted by $\overline{\partial }_b$ the maximal extension and by $\overline{\partial }_b^*$ the Hilbert space adjoint with respect to the $L^2$-inner product on *X*. We also denote by

2.8 $$\begin{aligned} \square _b=\overline{\partial }_b\overline{\partial }_b^*+\overline{\partial }_b^* \overline{\partial }_b: {{\,\mathrm{Dom}\,}}\square _b\subset L^2_{\bullet ,\bullet }(X)\rightarrow L^2_{\bullet ,\bullet }(X) \end{aligned}$$

the Gaffney extension of the Kohn Laplacian with the domain

2.9 $$\begin{aligned} {{\,\mathrm{Dom}\,}}\square _b=\big \{u\in L^2_{\bullet ,\bullet }(X): u\in {{\,\mathrm{Dom}\,}}\overline{\partial }_b\cap {{\,\mathrm{Dom}\,}}\overline{\partial }_b^*, ~\overline{\partial }_b u\in {{\,\mathrm{Dom}\,}}\overline{\partial }_b^*, ~\overline{\partial }_b^* u\in {{\,\mathrm{Dom}\,}}\overline{\partial }_b\big \}.\quad \end{aligned}$$

By a result of Gaffney, $\square _b$ is a self-adjoint operator (see e.g., [23, Proposition 3.1.2]).

### Pseudohermitian Geometry

The following is well known:

#### Proposition 2.4

[28, Proposition 3.1] Let $(X,HX,J,\omega _0)$ be an orientable strictly pseudoconvex CR manifold. Then there exists a unique affine connection, called Tanaka–Webster connection,

$$\begin{aligned} \nabla :=\nabla ^{\omega _0}: \mathscr {C}^\infty (X,TX)\rightarrow \mathscr {C}^\infty (X,T^*X\otimes TX) \end{aligned}$$

such that

(I) $$\nabla _U \mathscr {C}^\infty (X,HX)\subset \mathscr {C}^\infty (X,HX)$$ for $$U\in \mathscr {C}^\infty (X,TX)$$.
(II) $$\nabla T=\nabla J=\nabla d\omega _0=0$$.
(III) The torsion $T_\nabla$ of $\nabla$ satisfies: $T_\nabla (U, V)=d\omega _0(U, V)T$, $T_\nabla (T, JU)=-JT_\nabla (T, U)$, $U, V\in \mathscr {C}^\infty (X, HX).$

Recall that $\nabla J\in \mathscr {C}^\infty (X, T^* X\otimes \mathscr {L}(HX,HX))$, $\nabla d\omega _0\in \mathscr {C}^\infty (T^*X\otimes \Lambda ^2({\mathbb {C}} T^* X))$ are defined by $(\nabla _UJ)W=\nabla _U(JW)-J\nabla _UW$ and $\nabla _Ud\omega _0(W, V)=Ud\omega _0(W, V)-d\omega _0(\nabla _UW, V)- d\omega _0(W, \nabla _UV)$ for $U\in \mathscr {C}^\infty (X,TX), W, V\in \mathscr {C}^\infty (X,HX)$. Moreover, $\nabla J=0$ and $\nabla d\omega _0=0$ imply that the Tanaka–Webster connection is compatible with the Levi metric. By definition, the torsion of $\nabla$ is given by $T_\nabla (W, U)=\nabla _WU-\nabla _UW-[W, U]$ for $U, V\in \mathscr {C}^\infty (X,TX)$ and $\tau (T, U)$ for $U\in \mathscr {C}^\infty (X,HX)$ is called pseudohermitian torsion.

In the following, we will use the Einstein summation convention. Let $\{Z_\alpha \}_{\alpha =1}^n$ be a local frame of $T^{(1,0)}X$ and $\{\theta ^\alpha \}_{\alpha =1}^n$ be the dual frame of $\{Z_\alpha \}_{\alpha =1}^n$. We use the notations $Z_{\overline{\alpha }} :=\overline{Z_\alpha }$ and $\theta ^{\overline{\alpha }} =\overline{\theta ^\alpha }$. Write

$$\begin{aligned} \nabla Z_\alpha =\omega _\alpha ^\beta \otimes Z_\beta ,~\nabla Z_{\overline{\alpha }}= \omega _{\overline{\alpha }}^{\overline{\beta }} \otimes Z_{\overline{\beta }},~\text {and recall that}~\nabla T=0. \end{aligned}$$

We call $\omega _\alpha ^\beta$ the connection 1-form of Tanaka–Webster connection with respect to the frame $\{Z_\alpha \}_{\alpha =1}^n$. We denote by $\Theta _\alpha ^\beta$ the Tanaka–Webster curvature 2-form. Then,

$$\begin{aligned} \Theta _\alpha ^\beta =d\omega _\alpha ^\beta - \omega _\alpha ^\gamma \wedge \omega _\gamma ^\beta . \end{aligned}$$

By direct computation, we also have

$$\begin{aligned} \Theta _\alpha ^\beta =R_{\alpha j\overline{k}}^\beta \theta ^j \wedge \theta ^{\overline{k}}+A_{\alpha jk}^\beta \theta ^j \wedge \theta ^k+B_{\alpha jk}^\beta \theta ^{\overline{j}} \wedge \theta ^{\overline{k}}+C_0\wedge \omega _0, \end{aligned}$$

where $C_0$ is a 1-form. The term $R_{\alpha j\overline{k}}^\beta$ is called the pseudohermitian curvature tensor and the form

2.10 $$\begin{aligned} \begin{aligned} \mathrm{Ric\,}_{\mathscr {L}}:=R_{\alpha \overline{k}}\theta ^\alpha \wedge \theta ^{\overline{k}},\quad R_{\alpha \overline{k}} :=\sum _{j=1}^nR_{\alpha j\overline{k}}^j \end{aligned} \end{aligned}$$

is called pseudohermitian Ricci form.

### Strictly Pseudoconvex CR Manifolds with $\mathbb {R}$-Action

Let $(X,T^{(1,0)}X)$ be a CR manifold of $\dim X=2n+1$. Let $r: {\mathbb {R}}\times X\rightarrow X$, $r(x)=r\circ x$ for $r\in \mathbb {R}$, be an $\mathbb {R}$-action on *X*, see [13]. Let $\widehat{T}$ be the infinitesimal generator of the $\mathbb {R}$-action:

2.11 $$\begin{aligned} (\widehat{T}u)(x):=\frac{\partial }{\partial r}(u(r\circ x))\big |_{r=0}\,, \quad u\in \mathscr {C}^\infty (X). \end{aligned}$$

#### Definition 2.5

The $\mathbb {R}$-action is called locally free if $\widehat{T}(x)\ne 0$ at every $x\in X$.

By Assumption 1.1 we have

2.12 $$\begin{aligned}&\text {The } \mathbb {R}\text {-action is Cauchy--Riemann (CR)}: \big [\widehat{T},\mathscr {C}^{\infty }(X,T^{(1,0)}X)\big ]\subset \mathscr {C}^{\infty }(X,T^{(1,0)}X).\nonumber \\ \end{aligned}$$

2.13 $$\begin{aligned}&\text {The } \mathbb {R}\text {-action is transversal}: \mathbb {C}T_xX=T_x^{1,0}X\oplus T_x^{0,1}X\oplus \mathbb {C}\widehat{T}(x)~\text {at every}\, x\in X.\nonumber \\ \end{aligned}$$

Note that (2.12) implies that $L_{\widehat{T}}$ preserves *HX* and $[L_{\widehat{T}},J]=0$. Since $HX=\ker \omega _0$ we have for $U\in \mathscr {C}^{\infty }(X,HX)$,

$$\begin{aligned}&(L_{\widehat{T}}\omega _0)(U)=\widehat{T}(\omega _0(U))-\omega _0(L_{\widehat{T}}U)=0\\&(L_{\widehat{T}}\omega _0)(\widehat{T})=\widehat{T}(\omega _0(\widehat{T}))-\omega _0(L_{\widehat{T}}\widehat{T}) =\widehat{T}(\omega _0(\widehat{T})). \end{aligned}$$

We pose $f=\omega _0(\widehat{T})$ and $\omega _1=f^{-1}\omega _0$. Then $L_{\widehat{T}}\omega _1=0$ and $\omega _1(\widehat{T})=1$ since $(L_{\widehat{T}}\omega _1)(U)=0$ and $(L_{\widehat{T}}\omega _1)(\widehat{T})=\widehat{T}(\omega _1(\widehat{T}))=\widehat{T}(1)=0$. This also implies $\iota _{\widehat{T}}d\omega _1=0$. We have thus

$$\begin{aligned} \iota _{\widehat{T}}\omega _1=1,\,\,\iota _{\widehat{T}}d\omega _1=0,\,\, L_{\widehat{T}}J=0. \end{aligned}$$

We can therefore assume up to rescaling $\omega _0$ by a smooth function that the infinitesimal generator of the $\mathbb {R}$-action is a Reeb vector field $T=\widehat{T}$. This motivates the equality of the infinitesimal generator to the Reeb field in Assumption 1.1.

By [28, Lemma 3.2 (3)]) we have $2J\tau U=(L_T J )U$ for any $U\in HX$, hence the pseudohermitian torsion $\tau$ vanishes, which means that the contact metric manifold $(X,\omega _0,T,J,g_{\mathscr {L}})$ is a Sasakian manifold. Conversely, there exists a natural transversal CR $\mathbb {R}$-action on any compact Sasakian manifold. Recall that compact Sasakian manifolds can be classified in three categories based on the properties of the Reeb foliation consisting of the orbits of the Reeb field (see [7, Definition 6.1.25]). If the orbits of the Reeb field are all closed, then the Reeb field *T* generates a locally free, isometric $S^1$-action thus also an $\mathbb {R}$-action on $(X,g_{\mathscr {L}})$. In this case the Reeb foliation is called quasi-regular (and regular if the action is free). If the Reeb foliation is not quasi-regular, it is said to be irregular. In this case, *T* generates a transversal CR $\mathbb {R}$-action on *X*.

We use the local coordinates of Baouendi–Rothschild–Trevès (BRT charts) [3, Sect. 1], [19, Theorem 6.5] extensively as follows:

#### Theorem 2.6

(BRT charts) For each point $x\in X$, there exists a coordinate neighborhood $D=U\times \mathcal {I}$ with coordinates $x=(x_1,\ldots ,x_{2n+1})$ centered at 0, where $U=\{z=(z_1,\ldots ,z_n)\in \mathbb {C}^n:|z|<\epsilon \}$ and ${\mathcal {I}}=\{x_{2n+1}\in \mathbb {R}: |x_{2n+1}|<\epsilon _0 \}$, $\epsilon , \epsilon _0>0$, $z=(z_1,\ldots ,z_n)$ and $z_j=x_{2j-1}+\sqrt{-1}x_{2j}, j=1,\ldots ,n$, such that

2.14 $$\begin{aligned} T=\frac{\partial }{\partial x_{2n+1}}\quad \text {on}\quad D, \end{aligned}$$

and there exists $\phi \in \mathscr {C}^\infty (U,\mathbb {R})$ independent of $x_{2n+1}$ satisfying that

2.15 $$\begin{aligned} \left\{ Z_j :=\frac{\partial }{\partial z_j}+ i\frac{\partial \phi }{\partial z_j}(z)\frac{\partial }{\partial x_{2n+1}}\right\} _{j=1}^n \end{aligned}$$

is a frame of $T^{(1,0)}D$, and $\left\{ dz_j\right\} _{j=1}^n\subset T^{*(1,0)}D$ is the dual frame.

Let $D=U\times {\mathcal {I}}$ be a BRT chart. Let $f\in \mathscr {C}^\infty (D)$ and $u\in \Omega ^{p,q}(D)$ with $u=\sum _{I,J}u_{IJ}dz_I\wedge d\overline{z}_J$ with ordered sets *I*, *J* and $u_{IJ}\in \mathscr {C}^\infty (D)$, for all *I*, *J*. We have

2.16 $$\begin{aligned}&{df=\sum _{j=1}^nZ_j(f)dz_j+ \sum _{j=1}^n\overline{Z}_j(f)d\overline{z}_j+T(f)\omega _0},\end{aligned}$$

2.17 $$\begin{aligned}&\partial _b f =\sum _{j=1}^n Z_j(f)dz_j,\quad \overline{\partial }_b f =\sum _{j=1}^n\overline{Z}_j(f)d\overline{z}_j,\end{aligned}$$

2.18 $$\begin{aligned}&\partial _b u=\sum _{I,J} (\partial _b u_{IJ})\wedge dz_I\wedge d\overline{z}_J,\quad \overline{\partial }_b u =\sum _{I,J} (\overline{\partial }_b u_{IJ})\wedge dz_I\wedge d\overline{z}_J. \end{aligned}$$

For $u\in \Omega ^{p,q}(X)$, let $\mathcal {L}_T u$ be the Lie derivative of *u* in the direction of *T*. For simplicity, we write *Tu* to denote $\mathcal {L}_T u$. Since the $\mathbb {R}$-action is CR, $Tu\in \Omega ^{p,q}(X)$. On a BRT chart *D*, for $u\in \Omega ^{p,q}(D)$, $u=\sum _{I,J}u_{IJ}dz_I\wedge d{\overline{z}}_J$, we have $Tu=\sum _{I,J}(Tu_{IJ})\wedge dz_I\wedge d\overline{z}_J$ on *D*.

The Levi form $\mathscr {L}$ in a BRT chart $D\subset X$ has the form

2.19 $$\begin{aligned} \mathscr {L}=\partial \overline{\partial }\phi |_{T^{(1,0)}X}. \end{aligned}$$

Indeed, the characteristic 1-form $\omega _0$ and $d\omega _0$ on *D* are given by

2.20 $$\begin{aligned} \begin{aligned}&\omega _0(x)=dx_{2n+1}-i\sum _{j=1}^n\left( \frac{\partial \phi }{\partial z_j} dz_j-\frac{\partial \phi }{\partial \overline{z}_j}d\overline{z}_j\right) ,\\&d\omega _0(x)=2i\sum _{j,k=1}^n\frac{\partial ^2\phi }{\partial z_j\partial \overline{z}_k}dz_j\wedge d\overline{z}_k. \end{aligned} \end{aligned}$$

From now on, we assume that $\Theta _X$ is ${\mathbb {R}}$-invariant. Let $D=U\times {\mathcal {I}}$ be a BRT chart. The (1, 1) form $\Theta =\Theta _U$ on *U* is defined by, for $x=(z,x_{2n+1})\in D$,

2.21 $$\begin{aligned} \Theta (z):=\Theta _X(x). \end{aligned}$$

Note that it is independent of $x_{2n+1}$. More precisely,

2.22 $$\begin{aligned} \Theta (z)=\sqrt{-1}\sum ^n_{j,k=1}\left\langle Z_j|Z_k \right\rangle (x)dz_j\wedge d\overline{z}_k. \end{aligned}$$

Note that for another BRT coordinates $D=\widetilde{U}\times \widetilde{{\mathcal {I}}}$, $y=(w,y_{2n+1})$, there exist biholomorphic map $H\in \mathscr {C}^\infty (U,{\tilde{U}})$ and $G\in \mathscr {C}^\infty (U,{\mathbb {R}})$ such that $H(z)=w$, for all $z\in U$, $y_{2n+1}=x_{2n+1}+G(z)$, for all $(z,x_{2n+1})\in U\times {\mathcal {I}}$ and ${\tilde{U}}=H(U)$, $\tilde{{\mathcal {I}}}={\mathcal {I}}+G(U)$. We deduce that $\Theta$ is independent of the choice of BRT coordinates, i.e., $\Theta =\Theta _U=\Theta _{\widetilde{U}}$.

Until further notice, we work on a BRT chart $D=U\times {\mathcal {I}}$. For $p, q\in {\mathbb {N}}_0$, let $T^{*(p,q)}U$ be the bundle of (*p*, *q*) forms on *U* and let $T^{*\bullet ,\bullet }U:=\oplus _{p,q\in {\mathbb {N}}_0}T^{*(p,q)}U$. For $p, q\in {\mathbb {N}}_0$, let $T^{(p,q)}U$ be the bundle of (*p*, *q*) vector fields on *U* and let $T^{\bullet ,\bullet }U:=\oplus _{p,q\in {\mathbb {N}}_0}T^{(p,q)}U$. The (1, 1) form $\Theta$ induces Hermitian metrics on $T^{\bullet ,\bullet }U$ and $T^{*\bullet ,\bullet }U$. We shall use $\langle \,\cdot \,,\,\cdot \,\rangle _h$ to denote all the induced Hermitian metrics. The volume form on *U* induced by $\Theta$ is given by $d\lambda (z):=\Theta ^n/n!$. Thus, the volume form $dv_X$ can be represented by

2.23 $$\begin{aligned} dv_X(x)=d\lambda (z)\wedge dx_{2n+1} ~\text {on}~D. \end{aligned}$$

The $L^2$-inner product on $\Omega ^{\bullet ,\bullet }_c(U)$ with respect to $\Theta$ is given by

2.24 $$\begin{aligned} \langle s_1,s_2\rangle _{L^2(U)}:=\int _U\langle s_1(z),s_2(z) \rangle _h d\lambda (z), \ \ s_1, s_2\in \Omega ^{\bullet ,\bullet }_c(U). \end{aligned}$$

Let $t\in \mathbb {R}$ be fixed. The $L^2$-inner product on $\Omega ^{\bullet ,\bullet }_c(U)$ with respect to $\Theta$ and $e^{-2t\phi (z)}$ is given by

2.25 $$\begin{aligned} \langle s_1,s_2\rangle _{L^2(U,e^{-2t\phi })}:= \int _U\langle s_1(z),s_2(z) \rangle _h e^{-2t\phi (z)} d\lambda (z),\ \ s_1, s_2\in \Omega ^{\bullet ,\bullet }_c(U). \end{aligned}$$

The Chern curvature of $K_U^*:=\det (T^{(1,0)}U)$ with respect to $\Theta$ is given by

$$\begin{aligned} R^{K_U^*}:=\overline{\partial }\partial \log \det \left( \Big \langle \frac{\partial }{\partial z_j}, \frac{\partial }{\partial z_k}\Big \rangle _h\right) ^n_{j,k=1}, \quad R^{K_U^*}\in \Omega ^{1,1}(U). \end{aligned}$$

On *X*, define $K^*_X:=\det (T^{(1,0)}X)$. Then, $K^*_X$ is a CR line bundle over *X*. The Chern curvature $R^{K^*_X}$ of $K^*_X$ with respect to $\Theta _X$ is defined as follows: On a BRT chart *D*, let

2.26 $$\begin{aligned} R^{K_X^*}:=\overline{\partial }_b\partial _b\log \det \left( \langle Z_j|Z_k \rangle \right) _{j,k=1}^n. \end{aligned}$$

It is easy to see that $R^{K_X^*}$ is independent of the choice of BRT coordinates and hence $R^{K_X^*}$ is globally defined, i.e., $R^{K^*_X}\in \Omega ^{1,1}(X)$.

Let $$\{ L_j\}_{j=1}^n$$ be an $${\mathbb {R}}$$-invariant orthonormal frame of $$T^{(0,1)}D$$ with the dual (orthonormal) frame $$\{ e_j \}_{j=1}^n$$. Then $$\{ \overline{L}_j \}_{j=1}^n $$ is an $${\mathbb {R}}$$-invariant orthonormal frame of $$T^{(1,0)}D $$ with the dual (orthonormal) frame $$\{ \overline{e}_j \}_{j=1}^n$$. Since $$\Theta _X$$ is $${\mathbb {R}}$$-invariant, there exist $$c_j^k=c_j^k(z), w_j^k=w_j^k(z)\in \mathscr {C}^\infty (U)$$, $$j,k=1,\ldots ,n$$, satisfying $$\sum _{k=1}^n c_j^kw_k^l=\delta _j^l$$, for all $$j, l=1,\ldots ,n$$, such that for $$j=1,\ldots ,n$$,

2.27 $$\begin{aligned}&\overline{L}_j=\sum _{k=1}^n \overline{c}_j^k Z_k,\quad \quad \overline{e}_j=\overline{w}^j_k dz_k,\end{aligned}$$

2.28 $$\begin{aligned}&L_j=\sum _{k=1}^n c_j^k \overline{Z}_k,\quad \quad e_j= w^j_k d\overline{z}_k. \end{aligned}$$

We can check that $\{w_j:=\sum _{k=1}^n \overline{c}_j^k \frac{\partial }{\partial z_k};\, j=1,\ldots ,n\}$ and $\{\overline{w}_j:=\sum _{k=1}^n c_j^k \frac{\partial }{\partial \overline{z}_k};\, j=1,\ldots ,n\}$ are orthonormal frames for $T^{(1,0)}U$ and $T^{(0,1)}U$ with respect to $\Theta$, respectively, and $\{{\overline{e}}_j;\, j=1,\ldots ,n\}$, $\{e_j;\, j=1,\ldots ,n\}$ are dual frames for $\{w_j;\, j=1,\ldots ,n\}$ and $\{\overline{w}_j;\, j=1,\ldots ,n\}$, respectively. We also write $w^j$ and ${\overline{w}}^j$ to denote ${\overline{e}}_j$ and $e_j$, respectively, $j=1,\ldots ,n$.

#### Lemma 2.7

We have

$$\begin{aligned} \mathrm{Ric\,}_{\mathscr {L}}=R^{K^*_X}_{\mathscr {L}} \end{aligned}$$

on *X*.

#### Proof

Fix $p\in D$ and let $x=(x_1,\ldots ,x_{2n+1})$ be BRT local coordinates defined on an open set *D* of *p* with $x(p)=0$. We take $x=(x_1,\ldots ,x_{2n+1})=(z_1,\ldots ,z_n,x_{2n+1})$, $z_j=x_{2j-1}+ix_{2j}$, $j=1,\ldots ,n$, so that

2.29 $$\begin{aligned} \phi (z)=\frac{1}{2}\sum ^n_{j=1}|z_j|^2+O(|z|^4), \end{aligned}$$

where $\phi \in \mathscr {C}^\infty (D)$ is as in (2.15).

In the following, we will use Einstein summation convention. Write $\nabla _{Z_i}Z_j=\Gamma ^l_{ij}Z_l$, where $\nabla$ denotes the Tanaka–Webster connection (see Proposition 2.4). From [28, Lemma 3.2],

2.30 $$\begin{aligned} d\omega _0\left( \nabla _{Z_i}Z_j,\overline{Z}_k\right) =Z_i(d\omega _0(Z_j,\overline{Z}_k))- d\omega _0\left( Z_j,[Z_i,\overline{Z}_k]_{T^{(0,1)}X}\right) . \end{aligned}$$

Directly,

2.31 $$\begin{aligned}&d\omega _0\left( \nabla _{Z_i}Z_j,\overline{Z}_k\right) =d\omega _0\left( \Gamma ^l_{ij}Z_l,\overline{Z}_k\right) =2i\Gamma _{ij}^l\frac{\partial ^2\phi }{\partial z_l\partial \overline{z}_k}, \end{aligned}$$

2.32 $$\begin{aligned}&Z_i\left( d\omega _0\left( Z_j,\overline{Z}_k\right) \right) =2i\frac{\partial ^3\phi }{\partial z_i\partial z_j\partial \overline{z}_k}, \end{aligned}$$

$$\begin{aligned}{}[Z_i,\overline{Z}_k]|_{T^{(0,1)}X}=0 \end{aligned}$$

and hence

2.33 $$\begin{aligned} 2i\Gamma _{ij}^l\frac{\partial ^2\phi }{\partial z_l\partial \overline{z}_k} =2i\frac{\partial ^3\phi }{\partial z_i\partial z_j\partial \overline{z}_k}, \end{aligned}$$

for all $i, j, l, k=1,\ldots ,n$. Accordingly, by (2.29) and (2.33), we get that for all $i,j,k=1,\cdots ,n$,

2.34 $$\begin{aligned} \Gamma ^k_{ij}(0)=0. \end{aligned}$$

Moreover, by taking $\frac{\partial }{\partial \overline{z}_h}$ both sides in (2.33), from (2.29) and (2.33), it is not difficult to check that

2.35 $$\begin{aligned} \frac{\partial \Gamma ^k_{ij}}{\partial \overline{z}_h}(0) =2\frac{\partial ^4\phi }{\partial z_i\partial z_j\partial \overline{z}_k\partial \overline{z}_h}(0). \end{aligned}$$

It is clear that $\{dz_j\}_{j=1}^n$ and $\{d{\overline{z}}_j\}_{j=1}^n$ are the dual frames of $\{Z_j\}_{j=1}^n$ and $\{\overline{Z}_j\}_{j=1}^n$, respectively. Denote

$$\begin{aligned} \nabla Z_\alpha =\omega _\alpha ^\beta \otimes Z_\beta , \end{aligned}$$

and we can check that the (1, 1) part of $d\omega _\alpha ^\beta$ is

$$\begin{aligned} -\sum _{k,l=1}^n \left( \overline{Z}_l\Gamma ^\beta _{k\alpha })\right) dz_k\wedge d{\overline{z}}_l \end{aligned}$$

and the (1, 1) part of $\Theta _\alpha ^\beta =d\omega _\alpha ^\beta -\omega _\alpha ^\gamma \wedge \omega _\gamma ^\beta$ denoted by

$$\begin{aligned} \sum _{k,l=1}^n R_{\alpha k\overline{l}}^\beta \theta ^k\wedge \theta ^{\overline{l}} \end{aligned}$$

equals the (1, 1) part of $d\omega _\alpha ^\beta$. Hence, the pseudohermitian Ricci curvature tensor at origin is

$$\begin{aligned} R_{\alpha \overline{l}}(0)=\sum _{k=\beta =1}^n R_{\alpha k\overline{l}}^\beta (0) =-\sum _{k=\beta =1}^n\frac{\partial \Gamma ^\beta _{k\alpha }}{\partial \overline{z}_l}(0)= -2\sum _{k=1}^n\frac{\partial ^4\phi }{\partial z_k\partial \overline{z}_k \partial z_\alpha \partial \overline{z}_l}(0). \end{aligned}$$

We get that

2.36 $$\begin{aligned} \mathrm{Ric\,}_{\mathscr {L}}(0)=-2\sum _{k=1}^n\frac{\partial ^4\phi }{\partial z_k\partial \overline{z}_k \partial z_\alpha \partial \overline{z}_l}(0)dz_\alpha \wedge d{\overline{z}}_l. \end{aligned}$$

On the other hand, by directed computation, we can check that

2.37 $$\begin{aligned} R^{K_X^*}(0)=\overline{\partial }_b\partial _b\log \det \left( \langle Z_j|Z_k \rangle \right) _{j,k=1}^n(0) =-2\sum _{k=1}^n\frac{\partial ^4\phi }{\partial z_k\partial \overline{z}_k \partial z_\alpha \partial \overline{z}_l}(0)dz_\alpha \wedge d{\overline{z}}_l. \end{aligned}$$

From (2.36) and (2.37), the lemma follows. $\square$

## Bochner–Kodaira Formula on CR Manifolds with $\mathbb {R}$-Action

In this section, we will prove the Bochner–Kodaira–Nakano for CR manifolds with transversal CR $\mathbb {R}$-action. They are refinements of Tanaka’s basic identities [28, Theorems 5.1, 5.2] in our context. Namely, Tanaka’s formulas hold for any strictly pseudoconvex manifold endowed with the Levi metric, while our formulas are specific to CR manifolds with $\mathbb {R}$-action endowed with arbitrary Hermitian metric $\Theta _X$.

### The Fourier Transform on BRT Charts

Let $D=U\times {\mathcal {I}}$ be a BRT chart. Let $f\in \mathscr {C}^\infty _c(D)$. We write $f=f(x)=f(z,x_{2n+1})$. For each fixed $x_{2n+1}\in I$, $f(\cdot ,x_{2n+1})\in \mathscr {C}_c^\infty (U)$. For each fixed $z\in U$, $f(z,\cdot )\in \mathscr {C}_c^\infty ({\mathcal {I}})$. Let $p,q\in \mathbb {N}_0$, $u\in \Omega _c^{p,q}(D)$. We write $u=\sum _{I,J} u_{IJ}dz_I\wedge d\overline{z}_J\in \Omega ^{p,q}_c(D)$ and we always assume that the summation is performed only over increasingly ordered indices $I=i_1<i_2<\ldots<i_p, J=j_1<j_2<\ldots <j_q$, and $u_{IJ}\in \mathscr {C}^\infty _c(D)$, for all $\{I, J\}$. For each fixed $z\in U$, $u_{IJ}(z,\cdot )\in \mathscr {C}_c^\infty ({\mathcal {I}})$.

#### Definition 3.1

The Fourier transform of the function $f\in \mathscr {C}^\infty _c(D)$ with respect to $x_{2n+1}$, denoted by $\widehat{f}$, is defined by

3.1 $$\begin{aligned} \widehat{f}(z,t):=\int _{-\infty }^{\infty } e^{-itx_{2n+1}}f(z,x_{2n+1}) dx_{2n+1} \in \mathscr {C}^\infty (U\times {\mathbb {R}}). \end{aligned}$$

The Fourier transform of the form $u= \sum _{I,J} u_{IJ}dz_I\wedge d\overline{z}_J\in \Omega ^{p,q}_c(D)$ with respect to $x_{2n+1}$, denoted by $\widehat{u}$, is defined by

3.2 $$\begin{aligned} \widehat{u}(z,t)=\sum _{I,J}\widehat{u}_{IJ}(z,t)dz_I\wedge d\overline{z}_J\in \Omega ^{p,q}(U\times {\mathbb {R}}):= \mathscr {C}^\infty (U\times {\mathbb {R}}, T^{*(p,q)}U). \end{aligned}$$

Note that $\widehat{f}\in \mathscr {C}^\infty (U\times \mathbb {R})$ and $\widehat{f}(\cdot ,t)\in \mathscr {C}_c^\infty (U)$ for every $t\in \mathbb {R}$. Similarly, $\widehat{u}\in \Omega ^{p,q}(U\times \mathbb {R})$ and $\widehat{u}(\cdot ,t)\in \Omega ^{p,q}_c(U)$ for every $t\in \mathbb {R}$. From Parseval’s formula, we have for $u, v\in \Omega _c^{p,q}(D)$,

3.3 $$\begin{aligned} \int _{-\infty }^{\infty } \langle u(z,x_{2n+1})| v(z,x_{2n+1})\rangle dx_{2n+1}=(1/2\pi ) \int _{-\infty }^{\infty } \langle \widehat{u}(z,t), \widehat{v}(z,t)\rangle _h dt, \end{aligned}$$

for every $z\in U$. By using integration by parts, we have for $u\in \Omega ^{p,q}_c(D)$,

3.4 $$\begin{aligned} -\sqrt{-1}\widehat{Tu}=t\widehat{u},\quad , i.e., \quad ~-\sqrt{-1}\widehat{\frac{\partial u}{\partial x_{2n+1}}}(z,t)=t\widehat{u}(z,t). \end{aligned}$$

Let $t\in \mathbb {R}$ be fixed. Let $|(z,1)|^2_h:=e^{-2t\phi (z)}$ be the Hermitian metric on the trivial line bundle $U\times \mathbb {C}$ over *U*. The Chern connection of $(U\times \mathbb {C},e^{-2t\phi })$ is given by

3.5 $$\begin{aligned} \nabla ^{(U\times \mathbb {C},e^{-2t\phi })}= \nabla ^{1,0}+\nabla ^{0,1}, \quad \nabla ^{1,0}= \partial -2t\partial \phi , \quad \nabla ^{0,1}=\overline{\partial }. \end{aligned}$$

Indeed, $\nabla ^{(U\times \mathbb {C},e^{-2t\phi })}= d+h^{-1}\partial h=d+e^{2t\phi }\partial (e^{-2t\phi })$. The curvature of $(U\times \mathbb {C},e^{-2t\phi })$ is

3.6 $$\begin{aligned} R^{(U\times \mathbb {C},e^{-2t\phi })}= \left( \nabla ^{(U\times \mathbb {C},e^{-2t\phi })}\right) ^2=2t\partial \overline{\partial }\phi . \end{aligned}$$

We can identify $\partial \overline{\partial }\phi$ with Levi form $\mathscr {L}$ and write $R^{(U\times \mathbb {C},e^{-2t\phi })}=2t\mathscr {L}$. Moreover, we will identify $\Omega ^{\bullet ,\bullet }(U)$ and $\Omega ^{\bullet ,\bullet }_c(U)$ with $\Omega ^{\bullet ,\bullet }(U, U\times {\mathbb {C}})$ and $\Omega ^{\bullet ,\bullet }_c(U,U\times {\mathbb {C}})$, respectively.

#### Proposition 3.2

Let $u,v\in \Omega ^{\bullet ,\bullet }_c(D)$. We have

3.7 $$\begin{aligned}&\widehat{\overline{\partial }_b u}=e^{-t\phi }\overline{\partial }(e^{t\phi }\widehat{u}) \quad \text {on}~U\times \mathbb {R}, \end{aligned}$$

3.8 $$\begin{aligned}&\widehat{\overline{\partial }_b^* v}=e^{-t\phi }\overline{\partial }^*(e^{t\phi }\widehat{v})\quad \text {on}~U\times \mathbb {R}, \end{aligned}$$

3.9 $$\begin{aligned}&\widehat{\partial _b u}=e^{-t\phi }\nabla ^{1,0}(e^{t\phi }\widehat{u}) \quad \text {on}~U\times \mathbb {R}, \end{aligned}$$

3.10 $$\begin{aligned}&\widehat{\partial _b^*u}=e^{-t\phi }\nabla ^{1,0*}(e^{t\phi }\widehat{u})\quad \text {on}~U\times \mathbb {R}, \end{aligned}$$

where $\overline{\partial }^*,\nabla ^{1,0*}$ are the formal adjoints of $\overline{\partial },\nabla ^{1,0}$ with respect to $\langle \,\cdot \,,\,\cdot \,\rangle _{L^2(U,e^{-2t\phi })}$, respectively, and $\overline{\partial }_b^*$, $\partial ^*_b$ are the formal adjoints of $\overline{\partial }_b$, $\partial _b$ with respect to $(\,\cdot \,|\,\cdot \,)$, respectively.

#### Proof

Let $$u=\sum _{I,J}u_{IJ}dz_I\wedge d\overline{z}_J$$. By $$\overline{\partial }_b u=\sum _{I,J} \sum _{j=1}^n\left( \frac{\partial u_{IJ}}{\partial \overline{z}_j}- i\frac{\partial \phi }{\partial \overline{z}_j}\frac{\partial u_{IJ}}{\partial x_{2n+1}}\right) d\overline{z}_j\wedge dz_I\wedge d\overline{z}_J$$,

3.11 $$\begin{aligned} \begin{aligned} \left( \widehat{\overline{\partial }_b u}\right) (z,t)&=\sum _{I,J} \sum _{j=1}^n\left( \frac{\partial \widehat{u}_{IJ}}{\partial \overline{z}_j}(z,t) +t\frac{\partial \phi }{\partial \overline{z}_j}(z)\widehat{u}_{IJ}(z,t)\right) d\overline{z}_j\wedge dz_I\wedge d\overline{z}_J\\&=e^{-t\phi (z)}\overline{\partial }(e^{t\phi } \sum _{I,J}\widehat{u}_{IJ}dz_I\wedge d\overline{z}_{J})(z,t)\\&=\left( e^{-t\phi }\overline{\partial }(e^{t\phi }\widehat{u})\right) (z,t). \end{aligned} \end{aligned}$$

Thus the first equality holds. From Parseval’s formula,

3.12 $$\begin{aligned} (\overline{\partial }_b u|v)= & {} \int _D \langle \overline{\partial }_b u|v\rangle d\lambda (z)dx_{2n+1} \nonumber \\= & {} \int _{U}\left( (2\pi )^{-1}\int _{-\infty }^{\infty } \langle \widehat{\overline{\partial }_b u}(z,t),\widehat{v}(z,t)\rangle _h dt\right) d\lambda (z) \nonumber \\= & {} (2\pi )^{-1}\int _{-\infty }^{\infty }\int _U \langle e^{-t\phi }\overline{\partial }(e^{t\phi }\widehat{u}), \widehat{v}\rangle _h d\lambda (z)dt \nonumber \\= & {} (2\pi )^{-1}\int _{-\infty }^{\infty } \langle \overline{\partial }(e^{t\phi }\widehat{u}),e^{t\phi }\widehat{v} \rangle _{L^2(U,e^{-2t\phi })} dt \nonumber \\= & {} (2\pi )^{-1}\int _{-\infty }^{\infty } \langle e^{t\phi }\widehat{u} ,\overline{\partial }^*(e^{t\phi }\widehat{v}) \rangle _{L^2(U,e^{-2t\phi })} dt \nonumber \\= & {} (2\pi )^{-1}\int _{-\infty }^{\infty }\int _U \langle \widehat{u},e^{-t\phi } \overline{\partial }^*(e^{t\phi }\widehat{v}) \rangle _h d\lambda (z)dt. \end{aligned}$$

Meanwhile, we have

3.13 $$\begin{aligned} (\overline{\partial }_b u|v)=(u|\overline{\partial }_b^* v)= (2\pi )^{-1}\int _{-\infty }^{\infty }\int _{U}\langle \widehat{u}, \widehat{\overline{\partial }_b^* v}\rangle _h d\lambda (z)dt. \end{aligned}$$

Thus the second equality holds. The proofs of the third and the fourth equalities are similar. $\square$

### CR Bochner–Kodaira–Nakano Formula I

Analog to [23, (1.4.32)], we define the Lefschetz operator $\Theta _X\wedge \cdot$ on $\bigwedge ^{\bullet ,\bullet }(T^*X)$ and its adjoint $\Lambda =i(\Theta _X)$ with respect to the Hermitian inner product $\langle \cdot |\cdot \rangle$ associated with $\Theta _X$. The Hermitian torsion of $\Theta _X$ is defined by

3.14 $$\begin{aligned} \mathcal {T}:=[\Lambda ,\partial _b\Theta _X]. \end{aligned}$$

Let $D=U\times {\mathcal {I}}$ be a BRT chart and let $\{{\overline{L}}_j\}^n_{j=1}\subset T^{(1,0)}D$, $\{{\overline{e}}_j\}^n_{j=1}\subset T^{*(1,0)}D$, $\{w_j\}^n_{j=1}\subset T^{(1,0)}U$ be as in the discussion after (2.26). We can check that

3.15 $$\begin{aligned} \Theta _X\wedge \cdot =\sqrt{-1}\overline{e}_j\wedge e_j\wedge \cdot , \quad \Lambda =-\sqrt{-1}i_{L_j}i_{\overline{L}_j}\quad \text {on}~D. \end{aligned}$$

Note that $i_{L_j}$ and $i_{\overline{L}_j}$ are the adjoints of $e_j\wedge$ and $\overline{e}_j\wedge$, respectively.

Since $\partial \Theta (z)=\partial _b\Theta _X(x)$ on *D*, and $\Theta \wedge \cdot =\sqrt{-1}\overline{e}_j\wedge e_j\wedge \cdot$, $\Lambda =-\sqrt{-1}i_{\overline{w}_j}i_{w_j}$ on *U*, see [23, 1.4.32], we have $\mathcal {T}=[\Lambda ,\partial _b\Theta ]=[\Lambda ,\partial \Theta ]$ on $\Omega ^{\bullet ,\bullet }(D)$, which is independent of $x_{2n+1}$. We remark that $\mathcal {T}$ is a differential operator of order zero. With respect to the Hermitian inner product $\langle \cdot |\cdot \rangle$ associated with $\Theta _X$, we have the adjoint operator $\mathcal {T}^*$, the conjugate operator $\overline{\mathcal {T}}$ and the adjoint of the conjugate operator $\overline{\mathcal {T}}^*$ for $\mathcal {T}$.

#### Theorem 3.3

With the notations used above, we have on $\Omega ^{\bullet ,\bullet }(X)$,

3.16 $$\begin{aligned} \square _b=\overline{\square }_b+[2\sqrt{-1}\mathscr {L}, \Lambda ](-\sqrt{-1}T)+(\partial _b\,\mathcal {T}^*+\mathcal {T}^*\,\partial _b)- (\overline{\partial }_b\,\overline{\mathcal {T}}^*+\overline{\mathcal {T}}^*\,\overline{\partial }_b).\quad \end{aligned}$$

#### Proof

Since the both side of (3.16) are globally defined, we can check (3.16) on a BRT chart. Now, we work on a BRT chart $D=U\times {\mathcal {I}}$. We will use the same notations as before. Let

$$\begin{aligned}\begin{aligned}&\square ^{(U\times \mathbb {C}, e^{-2t\phi })}=\overline{\partial }\,\overline{\partial }^*+ \overline{\partial }^*\,\overline{\partial }: \Omega ^{\bullet ,\bullet }_c(U) \rightarrow \Omega ^{\bullet ,\bullet }_c(U),\\&\overline{\square }^{(U\times \mathbb {C}, e^{-2t\phi })}:= \nabla ^{1,0*}\nabla ^{1,0}+\nabla ^{1,0}\nabla ^{1,0*}: \Omega ^{\bullet ,\bullet }_c(U)\rightarrow \Omega ^{\bullet ,\bullet }_c(U), \end{aligned} \end{aligned}$$

where $\nabla ^{1,0}$ is given by (3.5), $\overline{\partial }^*,\nabla ^{1,0*}$ are the formal adjoints of $\overline{\partial },\nabla ^{1,0}$ with respect to $\langle \,\cdot \,,\,\cdot \,\rangle _{L^2(U,e^{-2t\phi })}$, respectively. From [23, (1.4.44)],

$$\begin{aligned} \square ^{(U\times \mathbb {C}, e^{-2t\phi })}= \overline{\square }^{(U\times \mathbb {C}, e^{-2t\phi })}+ [2\sqrt{-1}t\mathscr {L},\Lambda ]+(\nabla ^{1,0}\mathcal {T}^*+ \mathcal {T}^*\nabla ^{1,0})-(\overline{\partial }\,\overline{\mathcal {T}}^*+ \overline{\mathcal {T}}^*\,\overline{\partial }). \end{aligned}$$

Let $u, v\in \Omega ^{\bullet ,\bullet }_c(D)$. Let $s_1(z):=e^{t\phi (z)}\widehat{u}(z,t)\in \Omega ^{\bullet ,\bullet }(U\times {\mathbb {R}})$, $s_2(z):=e^{t\phi (z)}\widehat{v}(z,t)\in \Omega ^{\bullet ,\bullet }(U\times {\mathbb {R}})$. Firstly, we have

3.17 $$\begin{aligned} (1/2\pi )\int _{-\infty }^{\infty }\langle \square ^{(U\times \mathbb {C}, e^{-2t\phi })}s_1,s_2 \rangle _{L^2(U,e^{-2t\phi })}dt=(\,\overline{\partial }_b u\,|\,\overline{\partial }_bv\,) +(\,\overline{\partial }_b^* u\,|\,\overline{\partial }_b^*v\,).\quad \end{aligned}$$

In fact, from Proposition 3.2,

$$\begin{aligned} \begin{aligned}&\int _{-\infty }^{\infty }\langle \square ^{(U\times \mathbb {C}, e^{-2t\phi })}s_1,s_2\rangle _{L^2(U,e^{-2t\phi })}dt\\&=\int _{-\infty }^{\infty }\Bigr (\langle \overline{\partial }s_1,\overline{\partial }s_2 \rangle _{L^2(U,e^{-2t\phi })}+\langle \overline{\partial }^* s_1,\overline{\partial }^* s_2 \rangle _{L^2(U,e^{-2t\phi })}\Bigr )dt\\&=\int _{-\infty }^{\infty }\Bigr (\langle \widehat{ \overline{\partial }_bu}, \widehat{\overline{\partial }_bv} \rangle _{L^2(U)}+\langle \widehat{ \overline{\partial }_b^*u}, \widehat{\overline{\partial }_b^*v} \rangle _{L^2(U)}\Bigr )dt\\&=2\pi (\overline{\partial }_bu|\overline{\partial }_bv)+2\pi (\overline{\partial }_b^*u|\overline{\partial }_b^*v). \end{aligned} \end{aligned}$$

Similarly, we have

3.18 $$\begin{aligned} (1/2\pi )\int _{-\infty }^{\infty }\langle \overline{\square }^{(U\times \mathbb {C}, e^{-2t\phi })}s_1,s_2\rangle _{L^2(U,e^{-2t\phi })}dt= (\partial _bu|\partial _bv)+(\partial _b^*u|\partial _b^*v). \end{aligned}$$

Thirdly, we have

3.19 $$\begin{aligned} (1/2\pi )\int _{-\infty }^{\infty }t\langle [2\sqrt{-1}\mathscr {L},\Lambda ]s_1,s_2 \rangle _{L^2(U,e^{-2t\phi })}dt =([2\sqrt{-1}\mathscr {L},\Lambda ](-\sqrt{-1}T)u| v). \end{aligned}$$

In fact, it follows from

$$\begin{aligned} \begin{aligned}&\int _{-\infty }^{\infty }t\langle [2\sqrt{-1}\mathscr {L},\Lambda ]s_1,s_2 \rangle _{L^2(U,e^{-2t\phi })}dt\\&=\int _{-\infty }^{\infty }\langle t[2\sqrt{-1}\mathscr {L},\Lambda ]\widehat{u}, \widehat{v} \rangle _{L^2(U)}dt\\&=\int _{-\infty }^{\infty }\langle [2\sqrt{-1}\mathscr {L},\Lambda ] (-\widehat{\sqrt{-1}Tu}),\widehat{v}\rangle _{L^2(U)}dt\\&=2\pi ([2\sqrt{-1}\mathscr {L},\Lambda ]({-\sqrt{-1}Tu })|{v}). \end{aligned} \end{aligned}$$

Fourthly, we consider the rest terms

3.20 $$\begin{aligned} \langle (\nabla ^{1,0}\mathcal {T}^*+\mathcal {T}^*\nabla ^{1,0}) s_1,s_2 \rangle _{L^2(U,e^{-2t\phi })},\quad \langle (\nabla ^{0,1}\overline{\mathcal {T}}^*+\overline{\mathcal {T}}^*\nabla ^{0,1}) s_1, s_2 \rangle _{L^2(U,e^{-2t\phi })}. \end{aligned}$$

By Proposition 3.2, we have

3.21 $$\begin{aligned} \begin{aligned}&\int _{-\infty }^{\infty }\langle (\nabla ^{1,0}\mathcal {T}^*+ \mathcal {T}^*\nabla ^{1,0})s_1,s_2\rangle _{L^2(U,e^{-2t\phi })}dt\\&=\int _{-\infty }^{\infty }\langle \Bigr (\nabla ^{1,0}\mathcal {T}^*s_1,s_2 \rangle _{L^2(U,-2t\phi )}+\langle \mathcal {T}^*\nabla ^{1,0}s_1,s_2 \rangle _{L^2(U,-2t\phi )}\Bigr )dt\\&=\int _{-\infty }^{\infty }\langle \Bigr (\nabla ^{1,0}\mathcal {T}^* e^{t\phi } \widehat{u},e^{t\phi }\widehat{v}\rangle _{L^2(U,-2t\phi )}+ \langle \mathcal {T}^*\nabla ^{1,0}e^{t\phi }\widehat{u},e^{t\phi }\widehat{v} \rangle _{L^2(U,-2t\phi )}\Bigr )dt\\&=\int _{-\infty }^{\infty }\langle \Bigr (\mathcal {T}^* e^{t\phi }\widehat{u}, \nabla ^{1,0*}(e^{t\phi }\widehat{v})\rangle _{L^2(U,-2t\phi )}+ \langle \nabla ^{1,0}(e^{t\phi }\widehat{u}),\mathcal {T}e^{t\phi } \widehat{v}\rangle _{L^2(U,-2t\phi )}\Bigr )dt\\&=\int _{-\infty }^{\infty }\langle \Bigr (\mathcal {T}^*\widehat{u}, e^{-t\phi }\nabla ^{1,0*}(e^{t\phi }\widehat{v}) \rangle _{L^2(U)}+\langle e^{-t\phi }\nabla ^{1,0}e^{t\phi }\widehat{u}, \mathcal {T}\widehat{v}\rangle _{L^2(U)}\Bigr )dt\\&=\int _{-\infty }^{\infty }\langle \Bigr (\mathcal {T}^*\widehat{u}, \widehat{\partial _b^*v} \rangle _{L^2(U)}+\langle \widehat{\partial _bu}, \mathcal {T}\widehat{v} \rangle _{L^2(U)}\Bigr )dt\\&=2\pi (\mathcal {T}^*u|\partial _b^*v)+2\pi (\partial _b u|\mathcal {T}v). \end{aligned} \end{aligned}$$

Thus we obtain

3.22 $$\begin{aligned} \begin{aligned}&(1/2\pi )\int _{-\infty }^{\infty }\langle (\nabla ^{1,0}\mathcal {T}^*+ \mathcal {T}^*\nabla ^{1,0})s_1,s_2 \rangle _{L^2(U,e^{-2t\phi })}dt\\&=(\mathcal {T}^* u|\partial _b^* v)+(\partial _b u|\mathcal {T}v)\\&=((\partial _b\mathcal {T}^*+\mathcal {T}^*\partial _b)u|v). \end{aligned} \end{aligned}$$

Similarly, we obtain

3.23 $$\begin{aligned} (1/2\pi )\int _{-\infty }^{\infty }\langle (\overline{\partial }\,\overline{\mathcal {T}}^*+ \overline{\mathcal {T}}^*\overline{\partial })s_1,s_2 \rangle _{L^2(U,e^{-2t\phi })}dt= ((\overline{\partial }_b\overline{\mathcal {T}}^*+\overline{\partial }_b\overline{\mathcal {T}}^*)u|v). \end{aligned}$$

From (3.17), (3.18), (3.19), (3.22), and (3.23), we get that for $u, v\in \Omega ^{\bullet ,\bullet }_c(D)$,

$$\begin{aligned} (\square _b u|v)=((\overline{\square }_b+[2\sqrt{-1}\mathscr {L},\Lambda ](-\sqrt{-1}T)+ (\partial _b\mathcal {T}^*+\mathcal {T}^*\partial _b)-(\overline{\partial }_b\overline{\mathcal {T}}^*+\overline{\mathcal {T}}^*\overline{\partial }_b))u|v). \end{aligned}$$

The theorem follows. $\square$

#### Corollary 3.4

(CR Nakano’s inequality I) With the notations used above, for any $u\in \Omega _c^{\bullet ,\bullet }(X)$,

3.24 $$\begin{aligned} \begin{aligned} \frac{3}{2}(\square _b u|u)&\ge ([2\sqrt{-1}\mathscr {L},\Lambda ](-\sqrt{-1}Tu)|u)\\&-\frac{1}{2}(\Vert \mathcal {T}u\Vert ^2+\Vert \mathcal {T}^* u\Vert ^2+\Vert \overline{\mathcal {T}}u\Vert ^2+\Vert \overline{\mathcal {T}}^*u\Vert ^2). \end{aligned} \end{aligned}$$

If $(X,T^{(1,0)}X)$ is Kähler, i.e., $d\Theta _X=0$, then

3.25 $$\begin{aligned} (\square _b u|u)\ge ([2\sqrt{-1}\mathscr {L},\Lambda ](-\sqrt{-1}Tu)|u). \end{aligned}$$

#### Proof

By the Cauchy–Schwarz inequality, Theorem 3.3 and since $\mathcal {T}=0$, $\mathcal {T}^*=0$ if $d\Theta _X=0$, we get the corollary. $\square$

The following follows from straightforward calculation, we omit the proof.

#### Proposition 3.5

For a real (1, 1)-form $\sqrt{-1}\alpha \in \Omega ^{1,1}(D)$, if we choose local orthonormal frame $\{{\overline{L}}_j\}_{j=1}^n$ of $T^{(1,0)}D$ with the dual frame $\{{\overline{e}}_j\}_{j=1}^n$ of $T^{*(1,0)}D$ such that $\sqrt{-1}\alpha =\sqrt{-1}\lambda _j(x){\overline{e}}_j\wedge e_j$ at a given point $x\in D$, then for any $f=\sum _{I,J}f_{IJ}(x){\overline{e}}^I \wedge e^J \in \Omega ^{\bullet ,\bullet }(D)$, we have

3.26 $$\begin{aligned}{}[\sqrt{-1}\alpha ,\Lambda ]f(x)=\sum _{I,J}\left( \sum _{j\in I} \lambda _j(x)+\sum _{j\in J}\lambda _j(x)- \sum _{j=1}^n\lambda _j(x)\right) f_{IJ}(x){\overline{e}}^I \wedge e^J. \end{aligned}$$

#### Corollary 3.6

With the notations used above, let $\Theta _X$ be a Hermitian metric on *X* such that

3.27 $$\begin{aligned} 2\sqrt{-1}\mathscr {L}=\Theta _X. \end{aligned}$$

Then for any $u\in \Omega _c^{n,q}(X)$ with $1\le q\le n$,

3.28 $$\begin{aligned} \left( -\sqrt{-1}Tu|u\right) \le \frac{1}{q}\left( \Vert \overline{\partial }_b u\Vert ^2+\Vert \overline{\partial }^*_b u\Vert ^2\right) . \end{aligned}$$

#### Proof

By applying (3.26) for $\sqrt{-1}\alpha :=2\sqrt{-1}\mathscr {L}$, $\lambda _j=1$ for all *j*, we have

3.29 $$\begin{aligned}{}[2\sqrt{-1}\mathscr {L},\Lambda ](-\sqrt{-1}T u)= q(-\sqrt{-1}Tu),\ \ \text {for all}\, u\in \Omega ^{n,q}_c(X). \end{aligned}$$

By $d\Theta _X=d(2\sqrt{-1}\mathscr {L})=0$ and Corollary 3.4, we obtain

3.30 $$\begin{aligned} (\square _b u|u)\ge ([2\sqrt{-1}\mathscr {L},\Lambda ](-\sqrt{-1}Tu)|u)=q(-\sqrt{-1}Tu|u). \end{aligned}$$

$$\square $$

Let *E* be a CR line bundle over *X* (see Definition 2.4 in [13]. We say that *E* is a ${\mathbb {R}}$-equivariant CR line bundle over *X* if the ${\mathbb {R}}$-action on *X* can be CR lifted to *E* and for every point $x\in X$, we can find a *T*-invariant local CR frame of *E* defined near *x* (see [16, Definitions 2.6, 2.9]). Here, we also use *T* to denote the vector field acting on sections of *E* induced by the ${\mathbb {R}}$-action on *E*. From now on, we assume that *E* is a ${\mathbb {R}}$-equivariant CR line bundle over *X* with a ${\mathbb {R}}$-invariant Hermitian metric $h^E$ on *E*. For $p, q\in {\mathbb {N}}_0$, let $\Omega ^{p,q}(X,E)$ be the space of smooth (*p*, *q*)-forms of *X* with values in *E* and let $\Omega ^{\bullet ,\bullet }(X,E):= \oplus _{p,q\in {\mathbb {N}}_0}\Omega ^{p,q}(X,E)$. Let $\Omega ^{p,q}_c(X,E)$ be the subspace of $\Omega ^{p,q}(X,E)$ whose elements have compact support in *X* and let $\Omega ^{\bullet ,\bullet }_c(X,E):= \oplus _{p,q\in {\mathbb {N}}_0}\Omega ^{p,q}_c(X,E)$. For $p, q\in {\mathbb {N}}_0$, let

$$\begin{aligned} \overline{\partial }_{b,E}: \Omega ^{p,q}(X,E)\rightarrow \Omega ^{p,q+1}(X,E) \end{aligned}$$

be the tangential Cauchy–Riemann operator with values in *E*. Let $(\,\cdot \,|\,\cdot \,)_E$ be the $L^2$ inner product on $\Omega ^{\bullet ,\bullet }_c(X,E)$ induced by $\langle \,\cdot \,|\,\cdot \,\rangle$ and $h^E$. Let

$$\begin{aligned} \overline{\partial }^*_{b,E}: \Omega ^{p,q+1}(X,E)\rightarrow \Omega ^{p,q}(X,E) \end{aligned}$$

be the formal adjoint of $\overline{\partial }_{b,E}$ with respect to $(\,\cdot \,|\,\cdot \,)_E$. Put

$$\begin{aligned} \Box _{b,E}:=\overline{\partial }_{b,E}\,\overline{\partial }^*_{b,E}+ \overline{\partial }^*_{b,E}\,\overline{\partial }_{b,E}:\Omega ^{\bullet ,\bullet }(X,E) \rightarrow \Omega ^{\bullet ,\bullet }(X,E). \end{aligned}$$

Let

3.31 $$\begin{aligned} \nabla ^E: \Omega ^{\bullet ,\bullet }(X,E)\rightarrow \Omega ^{\bullet ,\bullet }(X,E\otimes \mathbb CT^*X) \end{aligned}$$

be the connection on *E* induced by $h^E$ given as follows: Let *s* be a *T*-invariant local CR frame of *E* on an open set *D* of *X*,

3.32 $$\begin{aligned} |s|^2_{h^E}=e^{-2\Phi },\ \ \Phi \in \mathscr {C}^\infty (D,{\mathbb {R}}). \end{aligned}$$

Then,

3.33 $$\begin{aligned} \nabla ^E(u\otimes s):=(\overline{\partial }_bu+\partial _bu-2(\partial _b\Phi )\wedge u +\omega _0\wedge (Tu))\otimes s, \ \ u\in \Omega ^{\bullet ,\bullet }(D). \end{aligned}$$

It is straightforward to check that (3.33) is independent of the choices of *T*-invariant local CR trivializing sections *s* and hence is globally defined. Put

3.34 $$\begin{aligned} (\nabla ^E)^{0,1}:=\overline{\partial }_b,\ \ (\nabla ^E)^{1,0}:= \partial _b-2\partial _b\Phi . \end{aligned}$$

Let

3.35 $$\begin{aligned} \overline{\Box }_{b,E}:=(\nabla ^E)^{1,0}((\nabla ^E)^{1,0})^*+ ((\nabla ^E)^{1,0})^*(\nabla ^E)^{1,0}: \Omega ^{\bullet ,\bullet }(X,E)\rightarrow \Omega ^{\bullet ,\bullet }(X,E), \end{aligned}$$

where $((\nabla ^E)^{1,0})^*$ is the adjoint of $(\nabla ^E)^{1,0}$ with respect to $(\,\cdot \,|\,\cdot \,)_E$. Let $R^E\in \Omega ^{1,1}(X)$ be the curvature of *E* induced by $h^E$ given by $R^E:=-2\overline{\partial }_b\partial _b\Phi$ on *D*, where $\Phi$ is as in (3.32). It is easy to check that $R^E$ is globally defined. Let $D=U\times {\mathcal {I}}$ be a BRT chart. Since *E* is ${\mathbb {R}}$-equivariant, on *D*, *E* is a holomorphic line bundle over *U*. We can repeat the proof of Theorem 3.3 with minor changes and conclude the following:

#### Theorem 3.7

Let *E* be a ${\mathbb {R}}$-equivariant CR line bundle over *X* with a ${\mathbb {R}}$-invariant Hermitian metric $h^E$. With the notations used above, we have on $\Omega ^{\bullet ,\bullet }(X,E)$,

3.36 $$\begin{aligned} \begin{aligned} \Box _{b,E}&=\overline{\Box }_{b,E}+[2\sqrt{-1}\mathscr {L},\Lambda ](-\sqrt{-1}T)+[\sqrt{-1}R^E, \Lambda ]\\&\quad +\Bigr ((\nabla ^E)^{1,0}\mathcal {T}^*+ \mathcal {T}^*(\nabla ^E)^{1,0}\Bigr )- \Bigr (\overline{\partial }_{b,E}\overline{\mathcal {T}}^*+ \overline{\mathcal {T}}^*\overline{\partial }_{b,E}\Bigr ), \end{aligned} \end{aligned}$$

where $R^E\in \Omega ^{1,1}(X)$ is the curvature of *E* induced by $h^E$.

### CR Bochner–Kodaira–Nakano Formula II

The bundle $K^*_X:=\det (T^{(1,0)}X)$ is a ${\mathbb {R}}$-equivariant CR line bundle over *X*. The (1, 1) form $\Theta _X$ induces a ${\mathbb {R}}$-invariant Hermitian metric $h^{K^*_X}$ on $K^*_X$. Let $R^{K^*_X}$ be the curvature of $K^*_X$ induced by $h^{K^*_X}$. Let

$$\begin{aligned} \Psi : T^{*0,q}X\rightarrow T^{*n,q}X\otimes K^*_X \end{aligned}$$

be the natural isometry defined as follows: Let $D=U\times {\mathcal {I}}$ be a BRT chart. Let $\{{\overline{L}}_j\}^n_{j=1}\subset T^{(1,0)}D$, $\{{\overline{e}}_j\}^n_{j=1}\subset T^{*(1,0)}D$ be as in the discussion after (2.26). Then,

$$\begin{aligned} \Psi u:={\overline{e}}_1\wedge \ldots \wedge {\overline{e}}_n \wedge u\otimes ({\overline{L}}_1\wedge \ldots \wedge {\overline{L}}_n)\in T^{*n,q}X\otimes K^*_X,\ \ u\in T^{*0,q}X. \end{aligned}$$

It is easy to see that the definition above is independent of the choices of ${\mathbb {R}}$-invariant orthonormal frame $\{{\overline{L}}_j\}^n_{j=1}\subset T^{(1,0)}D$ and hence is globally defined. We have the isometry:

$$\begin{aligned} \Psi : \Omega ^{0,q}(X)\rightarrow \Omega ^{n,q}(X,K^*_X). \end{aligned}$$

Moreover, it is straightforward to see that

3.37 $$\begin{aligned}&\overline{\partial }_bu=\Psi ^{-1}\overline{\partial }_{b,K^*_X}\Psi u,\ \ \overline{\partial }^*_bu\nonumber \\&\quad =\Psi ^{-1}\overline{\partial }^*_{b,K^*_X}\Psi u,\ \ \Box _bu=\Psi ^{-1}\Box _{b,K^*_X}\Psi u,\ \ \text {for every}\, u\in \Omega ^{0,q}(X).\nonumber \\ \end{aligned}$$

We can now prove

#### Theorem 3.8

With the notations used above, we have on $\Omega ^{0,\bullet }(X)$,

3.38 $$\begin{aligned} \begin{aligned} \square _b&=\Psi ^{-1}\Box _{b,K^*_X}\Psi + 2\mathscr {L}(\overline{L}_j,L_k)e_k\wedge i_{L_j}(-\sqrt{-1}T) +R^{K_X^*}(\overline{L}_j,L_k)e_k\wedge i_{L_j}\\&\quad +\Psi ^{-1}(\nabla ^{K^*_X})^{1,0} \mathcal {T}^*\Psi -\Bigr (\overline{\partial }_b\Psi ^{-1}\overline{\mathcal {T}}^*\Psi + \Psi ^{-1}\overline{\mathcal {T}}^*\Psi \overline{\partial }_b\Bigr ), \end{aligned} \end{aligned}$$

where $\{{\overline{L}}_j\}_{j=1}^n$ is a local ${\mathbb {R}}$-invariant orthonormal frame of $T^{(1,0)}X$ with dual frame $\{{\overline{e}}_j \}_{j=1}^n\subset T^{*(1,0)}X$.

#### Proof

Let $u\in \Omega ^{0,q}(X)$. From (3.37) and (3.36), we have

3.39 $$\begin{aligned} \begin{aligned} \Box _bu&=\Psi ^{-1}\Box _{b,K^*_X}\Psi u\\&=\Psi ^{-1}\overline{\Box }_{b,K^*_X}\Psi u+ \Psi ^{-1}[2\sqrt{-1}\mathscr {L},\Lambda ](-\sqrt{-1}T)(\Psi u)+ \Psi ^{-1}[\sqrt{-1}R^{K^*_X}, \Lambda ]\Psi u\\&\quad +\Psi ^{-1}\Bigr ((\nabla ^{K^*_X})^{1,0}\mathcal {T}^*+ \mathcal {T}^*(\nabla ^{K^*_X})^{1,0}\Bigr )\Psi u- \Bigr (\overline{\partial }_b\Psi ^{-1}\overline{\mathcal {T}}^*\Psi u+ \Psi ^{-1}\overline{\mathcal {T}}^*\Psi \overline{\partial }_bu\Bigr ). \end{aligned} \end{aligned}$$

It is straightforward to check that

3.40 $$\begin{aligned} \begin{aligned}&\qquad [2\sqrt{-1}\mathscr {L},\Lambda ]=2\mathscr {L}({\overline{L}}_j,L_k) ({\overline{e}}_j\wedge i_{{\overline{L}}_k}-i_{L_j}e_k\wedge ),\\&[2\sqrt{-1}R^{K^*_X},\Lambda ]= R^{K^*_X}({\overline{L}}_j,L_k)({\overline{e}}_j\wedge i_{{\overline{L}}_k}- i_{L_j}e_k\wedge ). \end{aligned} \end{aligned}$$

From (3.39), (3.40) and noting that $({\overline{e}}_j\wedge i_{{\overline{L}}_k}-i_{L_j}e_k\wedge )v= e_k\wedge i_{L_j}v$, $\mathcal {T}^*(\nabla ^{K^*_X})^{1,0}v=0$, for every $v\in \Omega ^{n,q}(X,K^*_X)$ and *T* commutes with $\Psi$, we get (3.38). $\square$

#### Corollary 3.9

With the notations used above, assume that $2\sqrt{-1}\mathscr {L}=\Theta _X$ and there is $C>0$ such that

$$\begin{aligned} \sqrt{-1}R^{K_X^*}\ge -C\Theta _X\ \ \text {on}\, X. \end{aligned}$$

Then, for any $u\in \Omega _c^{0,q}(X)$ with $1\le q\le n$, we have

3.41 $$\begin{aligned} \left( -\sqrt{-1}Tu|u\right) \le \frac{1}{q}\left( \Vert \overline{\partial }_b u\Vert ^2+ \Vert \overline{\partial }_b^* u\Vert ^2\right) +C\Vert u\Vert ^2. \end{aligned}$$

#### Proof

Since $2\sqrt{-1}\mathscr {L}=\Theta _X$, we can choose ${\mathbb {R}}$-invariant orthonormal frame $\{{\overline{L}}_j\}^n_{j=1}$ such that $2\mathscr {L}(\overline{L}_j,L_k)=\delta _{jk}$, for every *j*, *k*. We write $u=\sum _{J}u_Je_J$ on *D* with $u_J\in \mathscr {C}^\infty (D)$ and $e_J=e_{j_1}\wedge \ldots \wedge e_{j_q}$, $j_1<\ldots <j_q$. We have

3.42 $$\begin{aligned}&\langle \, 2\mathscr {L}(\overline{L}_j,L_k)e_k\wedge i_{L_j}(-\sqrt{-1}T)u\,|\,u\,\rangle =\langle \, \sum _J q(-\sqrt{-1}Tu_J)e_J\,|\,u\,\rangle \nonumber \\&\quad =q\langle \,-\sqrt{-1}Tu\,|\,u\,\rangle . \end{aligned}$$

Since $\sqrt{-1}R^{K_X^*}\ge -C\Theta _X$, as (3.42), we can check that

3.43 $$\begin{aligned} \langle R^{K_X^*}(\overline{L}_j,L_k)e_k\wedge i_{L_j} u|u\rangle \ge -Cq|u|^2. \end{aligned}$$

Since $d\Theta _X=d(2\sqrt{-1}\mathscr {L})=0$, we have $\mathcal {T}=[\Lambda ,\partial _b\Theta _X]=0$. From this observation, (3.38), (3.42), and (3.43), we obtain

3.44 $$\begin{aligned} \left( \Vert \overline{\partial }_b u\Vert ^2+\Vert \overline{\partial }_b^* u\Vert ^2\right) \ge q\left( -\sqrt{-1}Tu|u\right) -qC\Vert u\Vert ^2 \end{aligned}$$

holds for every $u\in \Omega _c^{0,q}(X)$ with $1\le q\le n$. $\square$

## Szegő Kernel Asymptotics

In this section, we will establish Szegő kernel asymptotic expansions on *X* under certain curvature assumptions.

### Complete CR Manifolds

Let *X* be a CR manifold as in Assumption 1.1. Let $g_X$ be the $\mathbb {R}$-invariant Hermitian metric as in (1.5). We will assume in the following that the Riemannian metric induced by $g_X$ on *TX* is complete and study the extension $\overline{\partial }_b$, $\overline{\partial }_b^*$, and *T*. We denote by the same symbols the maximal weak extensions in $L^2$ of these differential operators.

Since $g_X$ is complete we know by [10, Lemma 2.4, p. 366] that there exists a sequence $\{\chi _k\}^{\infty }_{k=1}\subset \mathscr {C}^\infty _c(X)$ such that $0\le \chi _k\le 1$, $\chi _{k+1}=1$ on $\mathrm{supp\,}\chi _k$, $|d\chi _k|_g\le \frac{1}{2^k}$, for every $k\in \mathbb {N}$, and $\bigcup ^{\infty }_{k=1}\mathrm{supp\,}\chi _k=X$. By using this sequence as in the Andreotti–Vesentini lemma on complex Hermitian manifolds (cf. [10, Theorem 3.2, p. 368], [23, Lemma 3.3.1]) and the classical Friedrichs lemma, we obtain the following.

#### Lemma 4.1

Assume that $(X,g_X)$ is complete. Then $\Omega _c^{p,q}(X)$ is dense in ${{\,\mathrm{Dom}\,}}\overline{\partial }_b$, ${{\,\mathrm{Dom}\,}}\overline{\partial }_b^*$, ${{\,\mathrm{Dom}\,}}T$, ${{\,\mathrm{Dom}\,}}\overline{\partial }_b\cap {{\,\mathrm{Dom}\,}}\overline{\partial }_b^*$, and ${{\,\mathrm{Dom}\,}}T\cap {{\,\mathrm{Dom}\,}}\overline{\partial }_b\cap {{\,\mathrm{Dom}\,}}\overline{\partial }_b^*$ with respect to the graph norms of $\overline{\partial }_b$, $\overline{\partial }_b^*$, *T*, $\overline{\partial }_b+\overline{\partial }_b^*$, and $\overline{\partial }_b+\overline{\partial }_b^*+T$. Here the graph norm of a linear operator *R* is defined by $\Vert u\Vert +\Vert Ru\Vert$ for $u\in {{\,\mathrm{Dom}\,}}R$.

As a consequence, analog to [23, Corollary 3.3.3], we obtain the following:

#### Corollary 4.2

If $(X,g_X)$ is complete, then the maximal extension of the formal adjoint of $\overline{\partial }_b$ and *T* coincide with their Hilbert space adjoint, respectively.

#### Corollary 4.3

If $(X,g_X)$ is complete, then $\sqrt{-1}T: {{\,\mathrm{Dom}\,}}\sqrt{-1}T\subset L^2_{\bullet ,\bullet }(X)\rightarrow L^2_{\bullet ,\bullet }(X)$ is self-adjoint, that is, $(\sqrt{-1}T)^*=\sqrt{-1}T$.

Using these results, we extend the estimates from Corollary 3.9 as follows:

#### Theorem 4.4

Let *X* be a CR manifold as in Assumption 1.1. Assume that $2\sqrt{-1}\mathscr {L}=\Theta _X$, $g_X$ is complete and there is $C>0$ such that

$$\begin{aligned} \sqrt{-1}R^{K^*_X}\ge -C\Theta _X. \end{aligned}$$

Then, for any $u\in L^2_{0,q}(X)$, $1\le q\le n$, $u\in {{\,\mathrm{Dom}\,}}\overline{\partial }_b\bigcap \mathrm{Dom}\overline{\partial }^*_b\bigcap {{\,\mathrm{Dom}\,}}(\sqrt{-1}T)$, we have

4.1 $$\begin{aligned} (-\sqrt{-1}Tu\,|\,u\,)\le \frac{1}{q}\Bigr (\Vert \overline{\partial }_bu\Vert ^2+ \Vert \overline{\partial }^*_bu\Vert ^2\Bigr )+C\Vert u\Vert ^2. \end{aligned}$$

#### Proof

Let $u\in L^2_{0,q}(X)$, $1\le q\le n$, $u\in {{\,\mathrm{Dom}\,}}\overline{\partial }_b\bigcap {{\,\mathrm{Dom}\,}}\overline{\partial }^*_b\bigcap {{\,\mathrm{Dom}\,}}(\sqrt{-1}T)$. From Lemma 4.1, we can find $\{u_j\}^{\infty }_{j=1}\subset \Omega ^{\bullet ,\bullet }_c(X)$ such that

4.2 $$\begin{aligned} \lim _{j\rightarrow \infty }\Bigr (\Vert u_j-u\Vert ^2+\Vert \overline{\partial }_bu_j-\overline{\partial }_bu\Vert ^2+ \Vert \sqrt{-1}Tu_j-\sqrt{-1}Tu\Vert ^2\Bigr )=0. \end{aligned}$$

From (3.41), we have for every $j=1,2,\ldots$,

4.3 $$\begin{aligned} (-\sqrt{-1}Tu_j\,|\,u_j\,)\le \frac{1}{q}\Bigr (\Vert \overline{\partial }_bu_j\Vert ^2+ \Vert \overline{\partial }^*_bu_j\Vert ^2\Bigr )+C\Vert u_j\Vert ^2. \end{aligned}$$

Taking $j\rightarrow \infty$ in (4.3) and using (4.2), we get (4.1). $\square$

Let us describe two examples of CR manifolds with complete $\mathbb {R}$-invariant metric $g_X$.

#### Example 4.5

Let $(X,HX,J,\omega _0)$ be a compact strictly pseudoconvex CR manifold as in Assumption 1.1 and let $g_X$ be a $\mathbb {R}$-invariant metric as in (1.5). Let $\pi :\widetilde{X}\rightarrow X$ be a Galois covering of *X*, that is, there exists a discrete, proper action $\Gamma$ such that $X=\widetilde{X}/\Gamma$. By pulling back the objects from *X* by the projection $\pi$ we obtain a strictly pseudoconvex CR manifold $(\widetilde{X},H\widetilde{X},\widetilde{J},\widetilde{\omega }_0)$ satisfying Assumption 1.1. Moreover, the metric $\widetilde{g}_X=\pi ^*g_X$ is a complete $\mathbb {R}$-invariant metric satisfying (1.5).

#### Example 4.6

Let us consider now the case of a circle bundle associated to a Hermitian holomorphic line bundle. Let $(M,J,\Theta _M)$ be a complete Hermitian manifold. Let $(L,h^L)\rightarrow M$ be a Hermitian holomorphic line bundle over *M*. Let $h^{L^*}$ be the Hermitian metric on $L^*$ induced by $h^L$. Let

4.4 $$\begin{aligned} X:=\{v\in L^*;\, |v|^2_{h^{L^*}}=1\} \end{aligned}$$

be the circle bundle of $L^*$; it is isomorphic to the $S^1$ principal bundle associated to *L*. Since *X* is a hypersurface in the complex manifold $L^*$, it has a CR structure (*X*, *HX*, *J*) inherited from the complex structure of $L^*$ by setting $T^{(1,0)}X= TX\cap T^{(1,0)} L^{*}$.

In this situation, $S^{1}$ acts on *X* by fiberwise multiplication, denoted $(x,e^{i\theta })\mapsto xe^{i\theta }$. A point $x\in X$ is a pair $x=(p,\lambda )$, where $\lambda$ is a linear functional on $L_p$, the $S^1$ action is $xe^{i\theta }=(p,\lambda )e^{i\theta }=(p,e^{i\theta }\lambda )$.

Let $\omega _0$ be the connection 1-form on *X* associated to the Chern connection $\nabla ^L$. Then

4.5 $$\begin{aligned} d\omega _0=\pi ^*(iR^L), \end{aligned}$$

where $R^L$ is the curvature of $\nabla ^L$. Assume $R^L$ is positive, hence *X* is a strictly pseudoconvex CR manifold. Hence, $(X,HX,J,\omega _0)$ fulfills Assumption 1.1. We denote by $\partial _\theta$ the infinitesimal generator of the $S^1$ action on *X*. The span of $\partial _\theta$ defines a rank one sub-bundle $T^VX\cong TS^1\subset TX$, the vertical sub-bundle of the fibration $\pi :X\rightarrow M$. Moreover (1.3) holds for $T=\partial _\theta$.

We construct now a Riemannian metric on *X*. Let $g_M$ be a *J*-invariant metric on *TM* associated to $\Theta _M$. The Chern connection $\nabla ^L$ on *L* induces a connection on the $S^1$-principal bundle $\pi :X\rightarrow M$, and let $T^H X \subset TX$ be the corresponding horizontal bundle. Let $g_X= \pi ^*g_M\oplus d\theta ^2/4\pi ^2$ be the metric on $TX= T^HX\oplus T S^1$, with $d\theta ^2$ the standard metric on $S^1= \mathbb {R}/2\pi \mathbb {Z}$. Then $g_X$ is a $\mathbb {R}$-invariant Hermitian metric on *X* satisfying (1.5). Since $g_M$ is complete it is easy to see that $g_X$ is also complete.

### The Operators $Q_\lambda$, $Q_{[\lambda _1,\lambda ]}$, $Q_{\tau }$

From now on, we assume that *X* is a CR manifold satisfying Assumption 1.1 and $(X,g_X)$ is complete. Let ${\mathbb {S}}$ denote the spectrum of $\sqrt{-1}T$. By the spectral theorem, there exists a finite measure $\mu$ on ${\mathbb {S}}\times {\mathbb {N}}$ and a unitary operator

$$\begin{aligned} U: L^2_{\bullet ,\bullet }(X)\rightarrow L^2({\mathbb {S}}\times {\mathbb {N}}, d\mu ) \end{aligned}$$

with the following properties: If $h: \mathbb S\times {\mathbb {N}}\rightarrow {\mathbb {R}}$ is the function $h(s,n)=s$, then the element $\xi$ of $L^2_{\bullet ,\bullet }(X)$ lies in ${{\,\mathrm{Dom}\,}}(\sqrt{-1}T)$ if and only if $hU(\xi )\in L^2(\mathbb S\times {\mathbb {N}}, d\mu )$. We have

$$\begin{aligned} U\sqrt{-1}TU^{-1}\varphi =h\varphi , \text {for all}\, \varphi \in U({{\,\mathrm{Dom}\,}}(\sqrt{-1}T)). \end{aligned}$$

Let $\lambda _1, \lambda \in {\mathbb {R}}$, $\lambda _1<\lambda$, and let $\tau (t)\in \mathscr {C}^\infty ({\mathbb {R}},[0,1])$. Put

4.6 $$\begin{aligned} \begin{aligned} \mathscr {E}(\lambda ,\sqrt{-1}T)&:=U^{-1}\Bigr (\mathrm{Image\,}U \cap \{\mathbb {1}_{(-\infty ,\lambda ]}(s)h(s,n);\, h(s,n)\in L^2({\mathbb {S}}\times {\mathbb {N}},d\mu )\}\Bigr ),\\ \mathscr {E}([\lambda _1,\lambda ],\sqrt{-1}T)&:= U^{-1}\Bigr (\mathrm{Image\,}U\cap \{\mathbb {1}_{[\lambda _1,\lambda ]}(s)h(s,n);\, h(s,n)\in L^2({\mathbb {S}}\times {\mathbb {N}},d\mu )\}\Bigr ),\\ \mathscr {E}(\tau ,\sqrt{-1}T)&:=U^{-1}\Bigr (\mathrm{Image\,}U\cap \{\tau (s)h(s,n);\, h(s,n)\in L^2({\mathbb {S}}\times {\mathbb {N}},d\mu )\}\Bigr ), \end{aligned} \end{aligned}$$

where $\mathbb {1}_A$ denotes the characteristic function of the set *A*. Let

4.7 $$\begin{aligned} \begin{aligned}&Q_\lambda : L^2_{\bullet ,\bullet }(X)\rightarrow \mathscr {E}(\lambda ,\sqrt{-1}T),\\&Q_{[\lambda _1,\lambda ]}: L^2_{\bullet ,\bullet }(X) \rightarrow \mathscr {E}([\lambda _1,\lambda ],\sqrt{-1}T),\\&Q_{\tau }: L^2_{\bullet ,\bullet }(X)\rightarrow \mathscr {E}(\tau ,\sqrt{-1}T) \end{aligned} \end{aligned}$$

be the orthogonal projections with respect to $(\,\cdot \,|\,\cdot \,)$.

Since *X* is strictly pseudoconvex, from [21, Lemma 3.4 (3), p. 239], [13, Theorem 3.5], we have one of the following two cases:

4.8 $$\begin{aligned} \begin{aligned}&\text {(a) The}\, {\mathbb {R}}\,\text {-action is free},\\&\text {(b) The}\, {\mathbb {R}}\,\text {-action comes from a CR torus action} {\mathbb {T}}^d \text {on}\, X \text {and}\, \omega _0 \text {and}\, \Theta _X \text {are}\, {\mathbb {T}}^d \text {invariant}. \end{aligned} \end{aligned}$$

When *X* is non-compact, the ${\mathbb {R}}$-action does not always come from a CR torus action. For example, when *X* is the Heisenberg group (see Sect. 5), the standard ${\mathbb {R}}$-action on *X* is free and does not come from any CR torus action.

Assume that the ${\mathbb {R}}$-action is free. Let $D=U\times {\mathcal {I}}$ be a BRT chart with BRT coordinates $x=(x_1,\ldots ,x_{2n+1})$. Since the ${\mathbb {R}}$-action is free, we can extend $x=(x_1,\ldots ,x_{2n+1})$ to ${\hat{D}}:=U\times {\mathbb {R}}$. We identify ${\hat{D}}$ with an open set in *X*.

#### Lemma 4.7

Assume that the ${\mathbb {R}}$-action is free. Let $D=U\times {\mathcal {I}}$ be a BRT chart with BRT coordinates $x=(x_1,\ldots ,x_{2n+1})$. Let $\lambda _1, \lambda \in {\mathbb {R}}$, $\lambda _1<\lambda$. For $u\in \Omega ^{\bullet ,\bullet }_c(D)$, we have

4.9 $$\begin{aligned} (Q_\lambda u)(x)&=\frac{1}{(2\pi )^{2n+1}} \int e^{i<x-y,\eta >}\mathbb {1}{(-\infty ,\lambda ]}(-\eta _{2n+1}) u(y)dyd\eta \in \Omega ^{\bullet ,\bullet }({\hat{D}}),\nonumber \\ \end{aligned}$$

4.10 $$\begin{aligned} (Q_{[\lambda _1,\lambda ]}u)(x)&=\frac{1}{(2\pi )^{2n+1}} \int e^{i<x-y,\eta >}\mathbb {1}{[\lambda _1,\lambda ]}(-\eta _{2n+1}) u(y)dyd\eta \in \Omega ^{\bullet ,\bullet }({\hat{D}}),\nonumber \\ \end{aligned}$$

4.11 $$\begin{aligned} (Q_{\tau }u)(x)&=\frac{1}{(2\pi )^{2n+1}}\int e^{i<x-y,\eta >} \tau (-\eta _{2n+1})u(y)dyd\eta \in \Omega ^{\bullet ,\bullet }({\hat{D}}), \end{aligned}$$

and $\mathrm{supp\,}Q_\lambda u\subset {\hat{D}}$, $\mathrm{supp\,} Q_{[\lambda _1,\lambda ]}u\subset {\hat{D}}$, $\mathrm{supp\,} Q_{\tau }u\subset {\hat{D}}$, where ${\hat{D}}$ is as in the discussion after (4.8).

#### Proof

Let $\chi \in \mathscr {C}^\infty _c({\mathbb {R}})$, $\chi =1$ on $[-1,1]$, $\chi =0$ outside $[-2,2]$. For every $M>0$, put $\tau _{M}(t):=\chi (\frac{t}{M})\tau (t)$. Then,

4.12 $$\begin{aligned} Q_{\tau }u=\lim _{M\rightarrow \infty }Q_{\tau _{M}}u\quad \text {in}\, L^2_{\bullet ,\bullet }(X), \text {for every}\, u\in L^2_{\bullet ,\bullet }(X). \end{aligned}$$

From the Helffer–Sjöstrand formula [12, Proposition 7.2], we see that

4.13 $$\begin{aligned} Q_{\tau _{M}}=\frac{1}{2\pi i} \int _{{\mathbb {C}}}\frac{\partial \tilde{\tau }_{M}}{\partial {\overline{z}}}(z-\sqrt{-1}T)^{-1} dz\wedge d{\overline{z}}\ \ \text {on}\, L^2_{\bullet ,\bullet }(X), \end{aligned}$$

where $\tilde{\tau }_{M}\in \mathscr {C}^\infty _c({\mathbb {C}})$ is an extension of $\tau _{M}$ with $\frac{\partial \tilde{\tau }_{M}}{\partial {\overline{z}}}=0$ on ${\mathbb {R}}$. It is not difficult to see that for $u\in \Omega ^{\bullet ,\bullet }_c(D)$,

4.14 $$\begin{aligned} (z-\sqrt{-1}T)^{-1}u=\frac{1}{(2\pi )^{2n+1}} \int e^{i<x-y,\eta >}\frac{1}{z+\eta _{2n+1}}u(y)dyd\eta \in \Omega ^{\bullet ,\bullet }({\hat{D}})\quad \end{aligned}$$

and $\mathrm{supp\,}(z-\sqrt{-1}T)^{-1}u\subset {\hat{D}}$. From (4.13) and (4.14), we have

4.15 $$\begin{aligned} (Q_{\tau _{M}}u)(x)=\frac{1}{2\pi i}\frac{1}{(2\pi )^{2n+1}} \int _{{\mathbb {C}}}\int e^{i<x-y,\eta >}\frac{\frac{\partial \tilde{\tau }_{M}}{\partial {\overline{z}}}}{z+\eta _{2n+1}}u(y)dyd\eta dz\wedge d{\overline{z}} \in \Omega ^{\bullet ,\bullet }({\hat{D}}) \end{aligned}$$

and $\mathrm{supp\,}Q_{\tau _{M}}u\subset {\hat{D}}$, for every $u\in \Omega ^{\bullet ,\bullet }_c(D)$. By Cauchy integral formula, we see that

$$\begin{aligned} \frac{1}{2\pi i}\int _{{\mathbb {C}}}\frac{1}{z+\eta _{2n+1}} \frac{\partial \tilde{\tau }_{M}}{\partial {\overline{z}}} dz\wedge d{\overline{z}}=\tau _{M}(-\eta _{2n+1}). \end{aligned}$$

From this observation and (4.15), we deduce that

4.16 $$\begin{aligned} (Q_{\tau _{M}}u)(x)=\frac{1}{(2\pi )^{2n+1}} \int e^{i\langle x-y,\eta \rangle }\tau _{M}(-\eta _{2n+1})u(y)dyd\eta \in \Omega ^{\bullet ,\bullet }({\hat{D}}) \end{aligned}$$

and $\mathrm{supp\,}Q_{\tau _{M}}u\subset {\hat{D}}$, for every $u\in \Omega ^{\bullet ,\bullet }_c(D)$. From (4.12) and (4.16), we get (4.11).

Let $\gamma _\varepsilon \in \mathscr {C}^\infty _c({\mathbb {R}})$, $\lim _{\varepsilon \rightarrow 0}\gamma _\varepsilon (t)= \mathbb {1}{(-\infty ,\lambda ]}(t)$, for every $t\in {\mathbb {R}}$. We can repeat the proof above and get that

$$\begin{aligned} Q_\lambda u=\lim _{\varepsilon \rightarrow 0}Q_{\gamma _\varepsilon }u= \frac{1}{(2\pi )^{2n+1}} \int e^{i\langle x-y,\eta \rangle }\mathbb {1}{(-\infty ,\lambda ]}(-\eta _{2n+1})u(y)dyd\eta \in \Omega ^{\bullet ,\bullet }({\hat{D}}) \end{aligned}$$

and $\mathrm{supp\,}Q_{\lambda }u\subset {\hat{D}}$, for every $u\in \Omega ^{\bullet ,\bullet }_c(D)$. We obtain (4.9). The proof of (4.10) is similar. $\square$

We now assume that the ${\mathbb {R}}$-action is not free. From (4.8), we know that the ${\mathbb {R}}$-action comes from a CR torus action $\mathbb T^d=(e^{i\theta _1},\ldots ,e^{i\theta _d})$ on *X* and $\omega _0$, $\Theta _X$ are ${\mathbb {T}}^d$ invariant. Since the ${\mathbb {R}}$-action comes from the ${\mathbb {T}}^d$-action, there exist $\beta _1,\ldots ,\beta _d\in {\mathbb {R}}$, such that

4.17 $$\begin{aligned} T=\beta _1T_1+\ldots +\beta _dT_d, \end{aligned}$$

where $T_j$ is the vector field on *X* given by $T_ju:= \frac{\partial }{\partial \theta _j}((1,\ldots ,1,e^{i\theta _j},1,\ldots ,1)^*u)$ $|_{\theta _j=0}$, $u\in \Omega ^{\bullet ,\bullet }(X)$, $j=1,\ldots ,d$. For $(m_1,\ldots ,m_d)\in {\mathbb {Z}}^d$, put

$$\begin{aligned}\begin{aligned}&L^{2,m_1,\ldots ,m_d}_{\bullet ,\bullet }(X)\\&:=\{u\in L^2_{\bullet ,\bullet }(X);\, (e^{i\theta _1},\ldots ,e^{i\theta _d})^*u= e^{im_1\theta _1+\ldots +im_d\theta _d}u, \text { for all}\, (\theta _1,\ldots ,\theta _d)\in \mathbb {R}^d\} \end{aligned} \end{aligned}$$

and let

4.18 $$\begin{aligned} Q_{m_1,\ldots ,m_d}: L^2_{\bullet ,\bullet }(X)\rightarrow L^{2,m_1,\ldots ,m_d}_{\bullet ,\bullet }(X) \end{aligned}$$

be the orthogonal projection. It is not difficult to see that for every $u\in L^2_{\bullet ,\bullet }(X)$, we have

4.19 $$\begin{aligned} \begin{aligned} Q_\lambda u&=\sum _{\begin{array}{c} (m_1,\ldots ,m_d)\in {\mathbb {Z}}^d,\\ -m_1\beta _1-\ldots -m_d\beta _d\le \lambda \end{array}}Q_{m_1,\ldots ,m_d}u,\\ Q_{[\lambda _1,\lambda ]}u&=\sum _{\begin{array}{c} (m_1,\ldots ,m_d)\in {\mathbb {Z}}^d,\\ \lambda _1\le -m_1\beta _1-\ldots -m_d\beta _d\le \lambda \end{array}}Q_{m_1,\ldots ,m_d}u,\\ Q_\tau u&=\sum _{(m_1,\ldots ,m_d)\in {\mathbb {Z}}^d} \tau (-m_1\beta _1-\ldots -m_d\beta _d)Q_{m_1,\ldots ,m_d}u. \end{aligned} \end{aligned}$$

From Lemma 4.7 and (4.19), we conclude that

#### Proposition 4.8

Let $\lambda _1, \lambda \in {\mathbb {R}}$, $\lambda _1<\lambda$. For $u\in {{\,\mathrm{Dom}\,}}\overline{\partial }_b$, we have $Q_\lambda u, Q_{[\lambda _1,\lambda ]}u, Q_\tau u\in {{\,\mathrm{Dom}\,}}\overline{\partial }_b$ and $\overline{\partial }_bQ_\lambda u=Q_\lambda \overline{\partial }_bu$, $\overline{\partial }_bQ_{[\lambda _1,\lambda ]} u=Q_{[\lambda _1,\lambda ]}\overline{\partial }_bu$, $\overline{\partial }_bQ_\tau u=Q_\tau \overline{\partial }_bu$. Similarly, for $u\in {{\,\mathrm{Dom}\,}}\overline{\partial }^*_b$, we have $Q_\lambda u, Q_{[\lambda _1,\lambda ]}u, Q_\tau u\in {{\,\mathrm{Dom}\,}}\overline{\partial }^*_b$ and $\overline{\partial }^*_bQ_\lambda u= Q_\lambda \overline{\partial }^*_bu$, $\overline{\partial }^*_bQ_{[\lambda _1,\lambda ]} u= Q_{[\lambda _1,\lambda ]}\overline{\partial }^*_bu$, $\overline{\partial }^*_bQ_\tau u= Q_\tau \overline{\partial }^*_bu$.

For $$\lambda \in {\mathbb {R}}$$, define

4.20 $$\begin{aligned} \begin{aligned}&\Box _{b,\lambda }: {{\,\mathrm{Dom}\,}}\Box _{b,\lambda } \subset \mathscr {E}(\lambda ,\sqrt{-1}T)\rightarrow \mathscr {E}(\lambda ,\sqrt{-1}T),\\ {{\,\mathrm{Dom}\,}}\Box _{b,\lambda }&:={{\,\mathrm{Dom}\,}}\Box _{b}\bigcap \mathscr {E}(\lambda ,\sqrt{-1}T),\,\, \Box _{b,\lambda }u=\Box _bu,\ \ \text {for }\,u\in {{\,\mathrm{Dom}\,}}\Box _{b,\lambda }, \end{aligned} \end{aligned}$$

where $\Box _{b}$ is defined in (2.8), (2.9). From Proposition 4.8, we see that

4.21 $$\begin{aligned} \begin{aligned}&{{\,\mathrm{Dom}\,}}\Box _{b,\lambda }=Q_\lambda ({{\,\mathrm{Dom}\,}}\Box _b),\\&Q_\lambda \Box _b=\Box _bQ_\lambda =\Box _{b,\lambda }Q_\lambda \ \ \text {on}\, {{\,\mathrm{Dom}\,}}\Box _b. \end{aligned} \end{aligned}$$

From now on, we write $\Box ^{(q)}_b$ and $\Box ^{(q)}_{b,\lambda }$ to denote $\Box _b$ and $\Box _{b,\lambda }$ acting on (0, *q*) forms, respectively.

### Local Closed Range for $\Box ^{(0)}_b$

In this section, we will establish the local closed range property for $\Box ^{(0)}_b$ under appropriate curvature assumptions. We first need the following.

#### Lemma 4.9

Assume that $2\sqrt{-1}\mathscr {L}=\Theta _X$, $g_X$ is complete and there is $C>0$ such that

$$\begin{aligned} \sqrt{-1}R^{K^*_X}\ge -C\Theta _X. \end{aligned}$$

Then, for any $u\in L^2_{0,q}(X)$, $1\le q\le n$, $u\in {{\,\mathrm{Dom}\,}}\overline{\partial }_b\cap {{\,\mathrm{Dom}\,}}\overline{\partial }^*_b\cap \mathscr {E}(\lambda ,\sqrt{-1}T)$, $\lambda \in {\mathbb {R}}$, $\lambda <-C$, we have

4.22 $$\begin{aligned} \Vert u\Vert ^2\le \frac{1}{q(-\lambda -C)}\Bigr (\Vert \overline{\partial }_bu\Vert ^2+\Vert \overline{\partial }^*_bu\Vert ^2\Bigr ). \end{aligned}$$

#### Proof

Let $\lambda <-C$ and let $u\in {{\,\mathrm{Dom}\,}}\overline{\partial }_b\cap {{\,\mathrm{Dom}\,}}\overline{\partial }^*_b\cap \mathscr {E}(\lambda ,\sqrt{-1}T)$, $u\in L^2_{0,q}(X)$, $1\le q\le n$. Let $M\gg 1$ and let $u_M:=Q_{[-M,\lambda ]}u$. By Proposition 4.8, we see that

$$\begin{aligned} u_M\in {{\,\mathrm{Dom}\,}}\overline{\partial }_b\cap {{\,\mathrm{Dom}\,}}\overline{\partial }^*_b\cap \mathscr {E}(\lambda ,\sqrt{-1}T)\bigcap {{\,\mathrm{Dom}\,}}(\sqrt{-1}T). \end{aligned}$$

From this observation and (4.1), we have

4.23 $$\begin{aligned} \begin{aligned} -\lambda \Vert u_M\Vert ^2&\le (\,-\sqrt{-1}Tu_M\,|\,u_M\,)\le \frac{1}{q}\Bigr (\Vert Q_{[-M,\lambda ]} \overline{\partial }_bu\Vert ^2+\Vert Q_{[-M,\lambda ]}\overline{\partial }^*_bu\Vert ^2\Bigr )+C\Vert u_M\Vert ^2\\&\le \frac{1}{q}\Bigr (\Vert \overline{\partial }_bu\Vert ^2+\Vert \overline{\partial }^*_bu\Vert ^2\Bigr )+C\Vert u_M\Vert ^2. \end{aligned} \end{aligned}$$

Since $\lambda <-C$ we deduce from (4.23) that

4.24 $$\begin{aligned} \Vert u_M\Vert ^2\le \frac{1}{q(-\lambda -C)}\Bigr (\Vert \overline{\partial }_bu\Vert ^2+\Vert \overline{\partial }^*_bu\Vert ^2\Bigr ). \end{aligned}$$

Letting $M\rightarrow \infty$ in (4.24) we get (4.22). $\square$

For every $q=0,1,\ldots ,n$, put $\mathscr {E}^{(q)}(\lambda ,\sqrt{-1}T):= \mathscr {E}(\lambda ,\sqrt{-1}T)\bigcap L^2_{0,q}(X)$. We prove now a vanishing theorem for harmonic forms which are eigenforms of $\sqrt{-1}T$.

#### Theorem 4.10

Assume that $2\sqrt{-1}\mathscr {L}=\Theta _X$, $g_X$ is complete and there is $C>0$ such that

$$\begin{aligned} \sqrt{-1}R^{K^*_X}\ge -C\Theta _X. \end{aligned}$$

Let $q\in \{1,\ldots ,n\}$. Let $\lambda \in {\mathbb {R}}$, $\lambda <-C$. The operator

$$\begin{aligned} \Box ^{(q)}_{b,\lambda }: {{\,\mathrm{Dom}\,}}\Box ^{(q)}_{b,\lambda }\subset \mathscr {E}^{(q)} (\lambda ,\sqrt{-1}T)\rightarrow \mathscr {E}^{(q)}(\lambda ,\sqrt{-1}T) \end{aligned}$$

has closed range and $\ker \Box ^{(q)}_{b,\lambda }=\{0\}$. Hence, there is a bounded operator

$$\begin{aligned} G^{(q)}_\lambda : \mathscr {E}^{(q)}(\lambda ,\sqrt{-1}T)\rightarrow {{\,\mathrm{Dom}\,}}\Box ^{(q)}_{b,\lambda } \end{aligned}$$

such that

4.25 $$\begin{aligned} \Box ^{(q)}_{b,\lambda }G^{(q)}_\lambda =I\ \ \text {on}\, \mathscr {E}^{(q)}(\lambda ,\sqrt{-1}T). \end{aligned}$$

#### Proof

Let $u\in {{\,\mathrm{Dom}\,}}\Box ^{(q)}_{b,\lambda }$. From (4.22), we have

$$\begin{aligned} \Vert u\Vert ^2\le \frac{1}{q(-\lambda -C)}\Bigr (\Vert \overline{\partial }_bu\Vert ^2+ \Vert \overline{\partial }^*_bu\Vert ^2\Bigr )= \frac{1}{q(-\lambda -C)}(\,\Box ^{(q)}_{b,\lambda }u\,|\,u\,). \end{aligned}$$

Hence,

4.26 $$\begin{aligned} \Vert u\Vert \le \frac{1}{q(-\lambda -C)}\Vert \Box ^{(q)}_{b,\lambda }u\Vert . \end{aligned}$$

From (4.26), the theorem follows. $\square$

We now consider (0, 1)-forms. Let $G^{(1)}_\lambda$ be as in (4.25). Since $G^{(1)}_\lambda$ is $L^2$ bounded, there is $C_0>0$ such that

4.27 $$\begin{aligned} \Vert G^{(1)}_\lambda v\Vert \le C_0\Vert v\Vert ,\ \ \text {for every}\, v\in \mathscr {E}^{(1)}(\lambda ,\sqrt{-1}T). \end{aligned}$$

We use the previous result to solve the $\overline{\partial }_b$-equation for eigenforms of $\sqrt{-1}T$.

#### Theorem 4.11

Assume that $2\sqrt{-1}\mathscr {L}=\Theta _X$, $g_X$ is complete and there is $C>0$ such that

$$\begin{aligned} \sqrt{-1}R^{K^*_X}\ge -C\Theta _X. \end{aligned}$$

Let $\lambda \in {\mathbb {R}}$, $\lambda <-C$. For every $v\in \mathscr {E}^{(1)}(\lambda ,\sqrt{-1}T)$ with $\overline{\partial }_bv=0$, we can find $u\in {{\,\mathrm{Dom}\,}}\overline{\partial }_b\bigcap \mathscr {E}^{(0)}(\lambda ,\sqrt{-1}T)$ such that

4.28 $$\begin{aligned} \begin{aligned}&\overline{\partial }_bu=v,\\&\Vert u\Vert ^2\le C_0\Vert v\Vert ^2, \end{aligned} \end{aligned}$$

where $C_0>0$ is a constant as in (4.27).

#### Proof

Let $v\in \mathscr {E}^{(1)}(\lambda ,\sqrt{-1}T)$ with $\overline{\partial }_bv=0$. From (4.25), we have

4.29 $$\begin{aligned} v=\overline{\partial }_b\,\overline{\partial }^*_bG^{(1)}_\lambda v+\overline{\partial }^*_b\overline{\partial }_bG^{(1)}_\lambda v. \end{aligned}$$

Since $\overline{\partial }_b\Bigr (\overline{\partial }^*_b\overline{\partial }_bG^{(1)}_\lambda v\Bigr )= \overline{\partial }_bv-\overline{\partial }^2_b\,\overline{\partial }^*_bG^{(1)}_\lambda v=0$, $\overline{\partial }^*_b\Bigr (\overline{\partial }^*_b\overline{\partial }_bG^{(1)}_\lambda v\Bigr )=0$, $\overline{\partial }^*_b\overline{\partial }_bG^{(1)}_\lambda v\in \ker \Box ^{(1)}_{b,\lambda }$. From Theorem 4.10, we see that $\overline{\partial }^*_b\overline{\partial }_bG^{(1)}_\lambda v=0$. From this observation and (4.29), we get $v=\overline{\partial }_bu$, $u=\overline{\partial }^*_bG^{(1)}_\lambda v$. Now,

$$\begin{aligned} \Vert u\Vert ^2&=\Vert \overline{\partial }^*_bG^{(1)}_\lambda v\Vert ^2\le \Vert \overline{\partial }_bG^{(1)}_\lambda v\Vert ^2+\Vert \overline{\partial }^*_bG^{(1)}_\lambda v\Vert ^2\\&=(\,\Box ^{(1)}_{b,\lambda }G^{(1)}_\lambda v\,|\,G^{(1)}_\lambda v\,)= (\,v\,|\,G^{(1)}_\lambda v\,)\le C_0\Vert v\Vert ^2, \end{aligned}$$

where $C_0>0$ is as in (4.27). The theorem follows. $\square$

Fix $q\in \{0,1,\ldots ,n\}$. Let $S^{(q)}: L^2_{0,q}(X)\rightarrow {{\,\mathrm{Ker}\,}}\square _b^{(q)}$ be the orthogonal projection with respect to $(\,\cdot \,|\,\cdot \,)$. From Proposition 4.8, we can check that

4.30 $$\begin{aligned} \begin{aligned} Q_\lambda S^{(q)}&=S^{(q)}Q_\lambda \text {on}\, L^2_{0,q}(X),\\ Q_{[\lambda _1,\lambda ]}S^{(q)}&=S^{(q)}Q_{[\lambda _1,\lambda ]} \text {on}\, L^2_{0,q}(X),\\ Q_\tau S^{(q)}&=S^{(q)}Q_\tau \text {on}\, L^2_{0,q}(X). \end{aligned} \end{aligned}$$

We recall the following notion introduced in [19, Definition 1.8].

#### Definition 4.12

Fix $q\in \{0,1,2,\ldots ,n\}$. Let $Q:L^2_{0,q}(X)\rightarrow L^2_{0,q}(X)$ be a continuous operator. We say that $\Box ^{(q)}_b$ has local $L^2$ closed range on an open set $D\subset X$ with respect to *Q* if for every $D'\Subset D$, there exist constants $C_{D'}>0$ and $p\in {\mathbb {N}}$, such that

$$\begin{aligned} \Vert Q(I-S^{(q)})u\Vert ^2\le C_{D'}\big (\,(\Box ^{(q)}_b)^pu\,|\,u\big ), \ \ \text {for all}\, u\in \Omega ^{0,q}_c(D'). \end{aligned}$$

We remind that we do not assume that $\Theta _X=2\sqrt{-1}\mathscr {L}$. The Levi form $2\sqrt{-1}\mathscr {L}$ induces a Hermitian metric $\langle \,\cdot \,|\,\cdot \,\rangle _{\mathscr {L}}$ on $\mathbb CTX$ and $\langle \,\cdot \,|\,\cdot \,\rangle _{\mathscr {L}}$ induces a Hermitian metric $\langle \,\cdot \,|\,\cdot \,\rangle _{\mathscr {L}}$ on $T^{*\bullet ,\bullet }X$. More precisely, if *X* is strictly pseudoconvex, i.e., $2\sqrt{-1}\mathscr {L}\in \Omega ^{1,1}(X)$ is positive definite, then we can construct a Hermitian metric $\langle \,\cdot \,|\,\cdot \,\rangle _{\mathscr {L}}$ on $\mathbb CTX=T^{(1,0)}X\oplus T^{(0,1)}X\oplus \mathbb {C}\{T\}$ in the following way: For arbitrary $a,b\in T^{(1,0)}X$, $\langle a| b\rangle _\mathscr {L}:=2\mathscr {L}(a,\overline{b})$, $\langle \overline{a}| \overline{b}\rangle _\mathscr {L}:=\langle b| a\rangle _\mathscr {L}$, $\langle a| \overline{b}\rangle _\mathscr {L}:=0$ and $\langle T| T\rangle _\mathscr {L}:=1$. We simply use $2\sqrt{-1}\mathscr {L}$ to represent $\langle \,\cdot \,|\,\cdot \,\rangle _{\mathscr {L}}$. Let $(\,\cdot \,|\,\cdot \,)_{\mathscr {L}}$ be the $L^2$ inner product on $\Omega ^{\bullet ,\bullet }_c(X)$ induced by $\langle \,\cdot \,|\,\cdot \,\rangle _{\mathscr {L}}$ and let $L^2_{\bullet ,\bullet }(X,\mathscr {L})$ be the completion of $\Omega ^{\bullet ,\bullet }_c(X)$ with respect to $(\,\cdot \,|\,\cdot \,)_{\mathscr {L}}$. We write $L^2(X,\mathscr {L}):=L^2_{0,0}(X,\mathscr {L})$. For $f\in L^2_{\bullet ,\bullet }(X,\mathscr {L})$, we write $\Vert f\Vert ^2_{\mathscr {L}}:=(\,f\,|\,f\,)_{\mathscr {L}}$.

Let $R^{K_X^*}_{\mathscr {L}}$ be the Chern curvature of $K^*_X$ with respect to the Hermitian metric $\langle ,\rangle _{\mathscr {L}}$ on *X*, see (2.26). Locally it can be represented by

4.31 $$\begin{aligned} R^{K_X^*}_{\mathscr {L}}= \overline{\partial }_b\partial _b\log \det \left( \langle Z_j|Z_k \rangle _{\mathscr {L}}\right) _{j,k=1}^n, \end{aligned}$$

where $\{Z_j\}^n_{j=1}\subset T^{(1,0)}X$ is as in (2.15).

For $u\in \Omega ^{\bullet ,\bullet }_c(X)$, from Lemma 4.7 and (4.19), we see that $Q_\lambda u$, $Q_{[\lambda _1,\lambda ]}u$, $Q_\tau u$ are independent of the choices of ${\mathbb {R}}$-invariant Hermitian metrics on *X*.

#### Theorem 4.13

Assume that $g_\mathscr {L}$ is complete and there is $C>0$ such that

4.32 $$\begin{aligned} \sqrt{-1}R^{K^*_X}_{\mathscr {L}}\ge -2C\sqrt{-1}\mathscr {L},\ \ {(2\sqrt{-1}\mathscr {L})^n\wedge \omega _0\ge C\Theta _X^n\wedge \omega _0.} \end{aligned}$$

Let $D\Subset X$ be an open set. Let $\lambda \in {\mathbb {R}}$, $\lambda <-C$. Then, $\square _b^{(0)}$ has local closed range on *D* with respect to $Q_\lambda$.

#### Proof

Let $u\in \mathscr {C}^\infty _c(D)$. Let $v:=\overline{\partial }_bQ_\lambda u =Q_\lambda \overline{\partial }_bu$. Since $\overline{\partial }_bu\in \Omega ^{0,1}_c(D)$,

$$\begin{aligned} Q_\lambda \overline{\partial }_bu\in L^2_{\bullet ,\bullet }(X,\mathscr {L})\bigcap L^2_{\bullet ,\bullet }(X). \end{aligned}$$

From Theorem 4.11, there exists $g\in L^2(X,\mathscr {L})$ with

4.33 $$\begin{aligned} \Vert g\Vert ^2_{\mathscr {L}}\le C_0\Vert \overline{\partial }_bQ_\lambda u\Vert ^2_{\mathscr {L}}\le C_0\Vert \overline{\partial }_bu\Vert ^2_{\mathscr {L}} \end{aligned}$$

such that

4.34 $$\begin{aligned} \overline{\partial }_bg=\overline{\partial }_bQ_\lambda u, \end{aligned}$$

where $C_0>0$ is a constant as in (4.28). Since $\overline{\partial }_b(I-S^{(0)})Q_\lambda u=\overline{\partial }_bQ_\lambda u$ and $(I-S^{(0)})Q_\lambda u\perp \ker \overline{\partial }_b$, we have

4.35 $$\begin{aligned} \Vert (I-S^{(0)})Q_\lambda u\Vert ^2\le \Vert g\Vert ^2\le \frac{1}{C}\Vert g\Vert ^2_{\mathscr {L}}, \end{aligned}$$

where $C>0$ is a constant as in (4.32). From (4.32), (4.33), and (4.35), we have

4.36 $$\begin{aligned} \Vert Q_\lambda (I-S^{(0)})u\Vert ^2= \Vert (I-S^{(0)})Q_\lambda u\Vert ^2\le \frac{1}{C}\Vert g\Vert ^2_{\mathscr {L}} \le \frac{C_0}{C}\Vert \overline{\partial }_bu\Vert ^2_{\mathscr {L}}. \end{aligned}$$

Since $\overline{\partial }_bu$ has compact support in *D*, there exists $C_1>0$ independent of *u* such that

4.37 $$\begin{aligned} \Vert \overline{\partial }_bu\Vert ^2_{\mathscr {L}}\le C_1\Vert \overline{\partial }_bu\Vert ^2. \end{aligned}$$

(4.36) and (4.37), the theorem follows. $\square$

For $\lambda \in {\mathbb {R}}$, $\lambda \le 0$, let $\tau _\lambda \in \mathscr {C}^\infty ({\mathbb {R}},[0,1])$, $\tau _\lambda =1$ on $]-\infty , 2\lambda ]$, $\tau _\lambda =0$ outside $(-\infty ,\lambda ]$. It is clear that $\Vert Q_{\tau _\lambda }(I-S^{(0)})u\Vert \le \Vert Q_{\lambda }(I-S^{(0)})u\Vert$, for every $u\in L^2(X)$. From this observation and Theorem 4.13, we deduce that

#### Theorem 4.14

Assume that $g_\mathscr {L}$ is complete and there is $C>0$ such that

4.38 $$\begin{aligned} \sqrt{-1}R^{K^*_X}_{\mathscr {L}}\ge -2C\sqrt{-1}\mathscr {L},\ \ {(2\sqrt{-1}\mathscr {L})^n\wedge \omega _0\ge C\Theta _X^n\wedge \omega _0.} \end{aligned}$$

Let $D\Subset X$ be an open set. Let $\lambda \in {\mathbb {R}}$, $\lambda <-C$. Then, $\square _b^{(0)}$ has local closed range on *D* with respect to $Q_{\tau _\lambda }$.

### Local Closed Range for $\square _{b}^{(n,0)}$

In this section, we will establish the local closed range property for $\Box ^{(n,0)}_b$ under appropriate curvature assumptions. We observe that the condition (4.32) can be removed, if we consider (*n*, 0)-forms instead of smooth function. We will adopt the same notation as before.

Let $\square _{b}$ be the Gaffney extension of the usual Kohn Laplacian. Let $\square _{b}^{(n,q)}$ be the restriction of $\square _{b}$ acting on (*n*, *q*)-forms. Set

4.39 $$\begin{aligned} \mathscr {E}^{(n,q)}(\lambda ,\sqrt{-1}T):=\mathscr {E}(\lambda ,\sqrt{-1}T)\cap L^2_{n,q}(X),\quad \square _{b,\lambda }^{(n,q)}:=\square _{b}^{(n,q)}|_{\mathscr {E}(\lambda ,\sqrt{-1}T)\cap {{\,\mathrm{Dom}\,}}\square _{b}^{(n,q)}}. \end{aligned}$$

Let $S^{(n,q)}: L^2_{n,q}(X)\rightarrow {{\,\mathrm{Ker}\,}}\square _{b}^{(n,q)}$ be orthogonal projection. It is known that $Q_\tau , Q_\lambda ,Q_{[\lambda _1,\lambda ]}$ commutes with $S^{(n,q)}$ on $L^2_{n,q}(X)$. Now, we present the main result of this section as follows:

#### Theorem 4.15

Let *X* be a CR manifold with a transversal CR $\mathbb {R}$-action. Let $\Theta _X$ be an $\mathbb {R}$-invariant Hermitian metric on *X*. Assume that $g_\mathscr {L}$ is complete. Let $D\Subset X$ be an open set. Let $\lambda \in {\mathbb {R}}$, $\lambda <0$. Then, $\square _b^{(n,0)}$ has local closed range on *D* with respect to $Q_\lambda$, i.e., there exists $C>0$ such that for all $u\in \Omega _c^{n,0}(D)$,

4.40 $$\begin{aligned} \Vert Q_\lambda (I-S^{(n,0)})u\Vert ^2\le C\Vert \overline{\partial }_b u\Vert ^2. \end{aligned}$$

This result is very natural in view of the Kodaira vanishing theorem, in the same way as Theorem 4.13 is parallel to the Kodaira–Serre type vanishing theorem. The proof is analog to the proof of Theorem 4.13.

Firstly, from Corollary 3.6 and the density Lemma 4.1, we obtain the following:

#### Lemma 4.16

With the notations used above, let $\Theta _X$ be a Hermitian metric on *X* such that

4.41 $$\begin{aligned} 2\sqrt{-1}\mathscr {L}=\Theta _X. \end{aligned}$$

Then for any $u\in L^2_{n,q}(X)\cap {{\,\mathrm{Dom}\,}}\overline{\partial }_b\cap {{\,\mathrm{Dom}\,}}\overline{\partial }^*_b \cap {{\,\mathrm{Dom}\,}}(\sqrt{-1}T)$, $1\le q \le n$, we have

4.42 $$\begin{aligned} \left( -\sqrt{-1}Tu|u\right) \le \frac{1}{q}\left( \Vert \overline{\partial }_b u\Vert ^2+ \Vert \overline{\partial }^*_b u\Vert ^2\right) . \end{aligned}$$

#### Lemma 4.17

Assume that $2\sqrt{-1}\mathscr {L}=\Theta _X$ and $g_X$ is complete. Then, for any $u\in L^2_{n,q}(X)\cap {{\,\mathrm{Dom}\,}}\overline{\partial }_b\cap {{\,\mathrm{Dom}\,}}\overline{\partial }^*_b\cap \mathscr {E}(\lambda ,\sqrt{-1}T)$, $\lambda <0$, and $1\le q\le n$, we have

4.43 $$\begin{aligned} \Vert u\Vert ^2\le \frac{1}{q(-\lambda )}\Bigr (\Vert \overline{\partial }_bu\Vert ^2+\Vert \overline{\partial }^*_bu\Vert ^2\Bigr ). \end{aligned}$$

#### Proof

Let $u\in L^2_{n,q}(X)\cap {{\,\mathrm{Dom}\,}}\overline{\partial }_b\cap {{\,\mathrm{Dom}\,}}\overline{\partial }^*_b \cap \mathscr {E}(\lambda ,\sqrt{-1}T)$, $\lambda <0$, and $1\le q\le n$. Let $M\gg 1$ be a sufficiently large positive real number such that $-M<\lambda$ and $u_M:=Q_{[-M,\lambda ]}u$. By Proposition 4.8, we see that

4.44 $$\begin{aligned} u_M\in L^2_{n,q}(X)\cap {{\,\mathrm{Dom}\,}}\overline{\partial }_b\cap {{\,\mathrm{Dom}\,}}\overline{\partial }^*_b \cap \mathscr {E}(\lambda ,\sqrt{-1}T)\cap {{\,\mathrm{Dom}\,}}(\sqrt{-1}T). \end{aligned}$$

Then

4.45 $$\begin{aligned} \begin{aligned} -\lambda \Vert u_M\Vert ^2&\le \left( -\sqrt{-1}Tu_M|u_M\right) \le \frac{1}{q}\left( \Vert \overline{\partial }_b u_M\Vert ^2+\Vert \overline{\partial }^*_b u_M\Vert ^2\right) \\&\le \frac{1}{q}\left( \Vert Q_{[-M,\lambda ]}\overline{\partial }_b u\Vert ^2 +\Vert Q_{[-M,\lambda ]}\overline{\partial }^*_b u\Vert ^2\right) . \end{aligned} \end{aligned}$$

By letting $M\rightarrow +\infty$ we complete the proof. $\square$

Moreover, we have the following analog of Theorem 4.10.

#### Theorem 4.18

Let *X* be a CR manifold with a transversal CR $\mathbb {R}$-action. Let $\Theta _X$ be an $\mathbb {R}$-invariant Hermitian metric on *X*. Assume that $2\sqrt{-1}\mathscr {L}=\Theta _X$ and $g_X$ is complete. Let $1\le q\le n$ and $\lambda <0$. Then the operator

4.46 $$\begin{aligned} \square _{b,\lambda }^{(n,q)}:{{\,\mathrm{Dom}\,}}\square _{b,\lambda }^{(n,q)}\subset \mathscr {E}^{(n,q)}(\lambda ,\sqrt{-1}T)\rightarrow \mathscr {E}^{(n,q)}(\lambda ,\sqrt{-1}T) \end{aligned}$$

has closed range, and

4.47 $$\begin{aligned} {{\,\mathrm{Ker}\,}}\square _{b,\lambda }^{(n,q)}=\{ 0\}. \end{aligned}$$

Hence, there exists a bounded operator

4.48 $$\begin{aligned} G_\lambda ^{(n,q)}:\mathscr {E}^{(n,q)}(\lambda ,\sqrt{-1}T)\rightarrow {{\,\mathrm{Dom}\,}}\square _{b,\lambda }^{(n,q)} \end{aligned}$$

such that

4.49 $$\begin{aligned} \square _{b,\lambda }^{(n,q)}G_\lambda ^{(n,q)}=I \quad \text {on}~\mathscr {E}^{(n,q)}(\lambda ,\sqrt{-1}T). \end{aligned}$$

Therefore, we have $C_0>0$ such that, for all $v\in \mathscr {E}^{(n,1)}(\lambda ,\sqrt{-1}T)$,

4.50 $$\begin{aligned} \Vert G^{(n,1)}_\lambda v\Vert \le C_0\Vert v\Vert . \end{aligned}$$

Secondly, we solve the $\overline{\partial }_b$-equation as follows.

#### Theorem 4.19

Let *X* be a CR manifold with a transversal CR $\mathbb {R}$-action. Let $\Theta _X$ be an $\mathbb {R}$-invariant Hermitian metric on *X*. Assume that $2\sqrt{-1}\mathscr {L}=\Theta _X$ and $g_X$ is complete. Then for every $\lambda <0$ and every $v\in \mathscr {E}^{(n,1)}(\lambda ,\sqrt{-1}T)$ with $\overline{\partial }_b v=0$, there exists $u\in {{\,\mathrm{Dom}\,}}\overline{\partial }_b\cap \mathscr {E}^{(n,0)}(\lambda ,\sqrt{-1}T)$ such that

4.51 $$\begin{aligned} \overline{\partial }_b u=v, \quad \Vert u\Vert ^2\le C_0\Vert v\Vert ^2. \end{aligned}$$

#### Proof

Let $\lambda <0$. Let $v\in \mathscr {E}^{(n,1)}(\lambda ,\sqrt{-1}T)$ with $\overline{\partial }_bv=0$. We have

4.52 $$\begin{aligned} v=\square _{b,\lambda }^{(n,1)}G_\lambda ^{(n,1)}v= \overline{\partial }_b\,\overline{\partial }^*_bG^{(n,1)}_\lambda v+ \overline{\partial }^*_b\overline{\partial }_bG^{(n,1)}_\lambda v. \end{aligned}$$

Since $$\overline{\partial }_b\Bigr (\overline{\partial }^*_b\overline{\partial }_bG^{(n,1)}_\lambda v\Bigr )= \overline{\partial }_bv-\overline{\partial }^2_b\,\overline{\partial }^*_bG^{(n,1)}_\lambda v=0$$, $$\overline{\partial }^*_b\Bigr (\overline{\partial }^*_b\overline{\partial }_bG^{(n,1)}_\lambda v\Bigr )=0$$,

4.53 $$\begin{aligned} \overline{\partial }^*_b\overline{\partial }_bG^{(n,1)}_\lambda v\in {{\,\mathrm{Ker}\,}}\Box ^{(n,1)}_{b,\lambda }. \end{aligned}$$

Thus, we see that $\overline{\partial }^*_b\overline{\partial }_bG^{(n,1)}_\lambda v=0$ and $v=\overline{\partial }_bu$ with $u=\overline{\partial }^*_bG^{(n,1)}_\lambda v$. Thus obtain

4.54 $$\begin{aligned} \begin{aligned} \Vert u\Vert ^2&=\Vert \overline{\partial }^*_bG^{(n,1)}_\lambda v\Vert ^2\le \Vert \overline{\partial }_bG^{(n,1)}_\lambda v\Vert ^2+ \Vert \overline{\partial }^*_bG^{(n,1)}_\lambda v\Vert ^2\\&=(\,\Box ^{(n,1)}_{b,\lambda }G^{(n,1)}_\lambda v\,|\,G^{(n,1)}_\lambda v\,)= (\,v\,|\,G^{(n,1)}_\lambda v\,)\\&\le C_0\Vert v\Vert ^2. \end{aligned} \end{aligned}$$

The proof is complete. $\square$

#### Proof of Theorem 4.15

Note that $\Theta _X$ is not necessarily equal to $2\sqrt{-1}\mathscr {L}$ so we have to deduce the general case to the particular case considered in Theorems 4.16–4.19. Let $\lambda <0$. Let $v:=\overline{\partial }_b Q_\lambda u=Q_\lambda \overline{\partial }_b u$. Note that $Q_\lambda$ is independent of the choice of $\Theta _X$. Then $v\in L^2_{n,1}(X,\mathscr {L})\cap L^2_{n,1}(X)$. From the above theorem, we can find $g\in L^2_{n,0}(X,\mathscr {L})$ such that $\overline{\partial }_b g=v$ and

4.55 $$\begin{aligned} \Vert g\Vert ^2_\mathscr {L}\le C_0\Vert v\Vert ^2_\mathscr {L}= C_0\Vert Q_\lambda \overline{\partial }_b u\Vert _\mathscr {L}^2\le C_0\Vert \overline{\partial }_b u\Vert ^2_\mathscr {L}. \end{aligned}$$

where the first inequality in (4.55) follows from Theorem 4.19. We claim that

$$\begin{aligned} \Vert g\Vert =\Vert g\Vert _\mathscr {L}\end{aligned}$$

and thus $g\in L^2_{n,0}(X,\mathscr {L})\cap L^2_{n,0}(X)$. In fact, we write locally

4.56 $$\begin{aligned} g=\alpha dz_1\wedge \cdots \wedge dz_n. \end{aligned}$$

With respect to $\Theta _X=\sqrt{-1}\langle Z_i|Z_j \rangle dz_i\wedge dz_j$ and $2\sqrt{-1}\mathscr {L}=\sqrt{-1}\langle Z_i|Z_j \rangle _\mathscr {L}dz_i\wedge dz_j$,

4.57 $$\begin{aligned} \begin{aligned} |g|^2&=|\alpha |^2\det (\langle Z_i|Z_j \rangle )^{-1},\\ |g|^2_\mathscr {L}&=|\alpha |^2\det (\langle Z_i|Z_j \rangle _\mathscr {L})^{-1}, \end{aligned} \end{aligned}$$

and, respectively, the volume forms are given by

4.58 $$\begin{aligned} \begin{aligned} \Theta _X^n\wedge \omega _0&=n!(\sqrt{-1})^n\det (\langle Z_i|Z_j \rangle )dz_1\wedge d\overline{z}_1\wedge \cdots \wedge dz_n\wedge d\overline{z}_n\wedge \omega _0,\\ (2\sqrt{-1}\mathscr {L})^n\wedge \omega _0&=n!(\sqrt{-1})^n\det (\langle Z_i|Z_j \rangle _\mathscr {L})dz_1\wedge d\overline{z}_1\wedge \cdots \wedge dz_n\wedge d\overline{z}_n\wedge \omega _0. \end{aligned} \end{aligned}$$

Thus, the claim follows from

4.59 $$\begin{aligned} \Vert g\Vert ^2=\int _X |g|^2 \Theta _X^n\wedge \omega _0=\int _X |g|_\mathscr {L}^2 (2\sqrt{-1}\mathscr {L})^n\wedge \omega _0=\Vert g\Vert ^2_\mathscr {L}. \end{aligned}$$

Since $\overline{\partial }_b(I-S^{(n,0)})Q_\lambda u=\overline{\partial }_b Q_\lambda u=v$ and $(I-S^{(n,0)})Q_\lambda u \perp {{\,\mathrm{Ker}\,}}\overline{\partial }_b$, we have $\overline{\partial }_b(I-S^{(n,0)})Q_\lambda u$ is the solution of minimal norm with respect to $\Theta _X$, i.e.,

4.60 $$\begin{aligned} \Vert \overline{\partial }_b(I-S^{(n,0)})Q_\lambda u\Vert ^2\le \Vert g\Vert ^2=\Vert g\Vert _\mathscr {L}^2\le C_0\Vert \overline{\partial }_b u\Vert _\mathscr {L}^2\le C_0C_1\Vert \overline{\partial }_b u\Vert ^2 \end{aligned}$$

by $${{\,\mathrm{supp}\,}}(u)\Subset D$$. $$\square $$

### $L^2$ Estimates for $\overline{\partial }_{b,E}$

In this section, we prove an analog for the $\overline{\partial }_{b,E}$-operator of the $L^2$-estimates of the Hörmander–Andreotti–Vesentini estimates for $\overline{\partial }$. As in the case of complex manifolds, we use the Bochner–Kodaira–Nakano formula in the present form (3.36). In order to eliminate the first-order error term $[(\nabla ^E)^{1,0},\mathcal {T}^*]-[\overline{\partial }_{b,E},\overline{\mathcal {T}}^*]$ in (3.36), we reformulate (3.36) can be reformulated as in [10, VII.1]. Under the hypothesis of Theorem 3.7, we have on $\Omega ^{\bullet ,\bullet }(X,E)$,

4.61 $$\begin{aligned} \square _b^E=\overline{\square }_{b,\mathcal {T}}^E+[2\mathscr {L}T+\sqrt{-1}R^E, \Lambda ]+\mathcal {T}_\Theta , \end{aligned}$$

where $\overline{\square }_{b,\mathcal {T}}^E:=[(\nabla ^E)^{1,0}+\mathcal {T}, (\nabla ^E)^{1,0*}+\mathcal {T}^*]$ is a positive formally self-adjoint operator, and

4.62 $$\begin{aligned} \mathcal {T}_\Theta :=[\Lambda , [\Lambda , \frac{\sqrt{-1}}{2}\partial _{b}\overline{\partial }_{b}\Theta _X]]-[\overline{\partial }_{b}\Theta _X,(\overline{\partial }_{b}\Theta _X)^*] \end{aligned}$$

is an operator of order zero depending only on the torsion of Hermitian metric $\Theta _X$.

#### Theorem 4.20

($L^2$-estimates for $\overline{\partial }_b$) Let *X* be a CR manifold with a smooth locally free CR $\mathbb {R}$-action. Let $\Theta _X$ be an $\mathbb {R}$-invariant Hermitian metric on *X*. Assume $g_X$ is complete. Let *E* be a ${\mathbb {R}}$-equivariant CR line bundle over *X* with a ${\mathbb {R}}$-invariant Hermitian metric $h^E$. Assume that for some (*r*, *q*), $q\ge 1$, there exists a function $\psi : X\rightarrow [0,\infty )$ such that, for all $s\in \Omega _c^{r,q}(X,E)$, pointwisely

4.63 $$\begin{aligned} \left\langle [2\mathscr {L}T+\sqrt{-1}R^E, \Lambda ]+ \mathcal {T}_\Theta s|s\right\rangle \ge \psi |s|^2. \end{aligned}$$

Then, for any $f\in L^2_{r,q}(X,E)$ satisfying $\overline{\partial }_{b,E} f=0$ and $\int _X \psi ^{-1}|f|^2dv_X< \infty$, there exists $g\in L^2_{r,q-1}(X,E)$ such that $\overline{\partial }_{b,E} g=f$ and $\Vert g\Vert ^2\le \int _X \psi ^{-1}|f|^2dv_X$.

#### Proof

Consider the complex of closed densely defined operators

4.64 $$\begin{aligned} L^2_{r,q-1}(X,E)\xrightarrow {T=\overline{\partial }_{b,E}} L^2_{r,q}(X,E)\xrightarrow {S=\overline{\partial }_{b,E}}L^2_{r,q+1}(X,E), \end{aligned}$$

where *T* and *S* are maximal extensions of $\overline{\partial }_{b,E}$. We apply (4.61) and obtain for all $s\in \Omega _c^{r,q}(X,E)$, it follows that

4.65 $$\begin{aligned} \Vert \overline{\partial }_{b,E} s\Vert ^2+\Vert \overline{\partial }_{b,E}^*s\Vert ^2\ge \left( [2\mathscr {L}T+\sqrt{-1}R^E, \Lambda ]+\mathcal {T}_\Theta s|s\right) \ge \int _X\psi |s|^2dv_X.\quad \end{aligned}$$

By Cauchy–Schwarz inequality,

4.66 $$\begin{aligned} |(f|s)|^2=|(\psi ^{-1/2}f|\psi ^{1/2}s)|^2\le \int _X \psi ^{-1}|f|^2dv_X \cdot \left( \Vert \overline{\partial }_{b,E} s\Vert ^2+\Vert \overline{\partial }_{b,E}^*\Vert ^2 \right) \end{aligned}$$

Since $g_X$ is complete, the above inequality still holds for all $s\in {{\,\mathrm{Dom}\,}}(S)\cap {{\,\mathrm{Dom}\,}}(T^*)$ by the density Lemma 4.1. Consider now $s\in {{\,\mathrm{Dom}\,}}(T^*)$ and write the orthogonal decomposition $s=s_1+s_2$ with $s_1\in {{\,\mathrm{Ker}\,}}(S)$ and $s_2\in {{\,\mathrm{Ker}\,}}(S)^\perp \subset [\mathrm{Im}(S^*)]\subset {{\,\mathrm{Ker}\,}}T^*$. So $s_1=s-s_2\in {{\,\mathrm{Ker}\,}}(S)\cap {{\,\mathrm{Dom}\,}}(T^*)$. Recall $f\in {{\,\mathrm{Ker}\,}}(S)$,

4.67 $$\begin{aligned} |(f|s)|^2=|(f|s_1)|^2\le \int _X \psi ^{-1}|f|^2dv_X\cdot \Vert T^* s_1\Vert ^2 =\int _X \psi ^{-1}|f|^2dv_X\cdot \Vert T^* s\Vert ^2. \end{aligned}$$

We consider $\lambda :\mathrm{Im}(T^*)\rightarrow \mathbb {C}$ defined by $\lambda (T^* s)=(f|s)$ for $s\in {{\,\mathrm{Dom}\,}}(T^*)$. We see that $\lambda$ is $\mathbb {C}$-antilinear map and $|\lambda (T^*s)|\le (\int _X\psi ^{-1}|f|^2dv_X)^{1/2}\Vert T^*s\Vert$, i.e., $\lambda$ is bounded with norm $\Vert \lambda \Vert \le (\int _X\psi ^{-1}|f|^2dv_X)^{1/2}$. By the complex Hahn–Banach theorem we can extend $\lambda$ to $L^2_{r,q-1}(X,E)$ with the same norm $\Vert \lambda \Vert \le (\int _X\psi ^{-1}|f|^2dv_X)^{1/2}$.

By the Riesz representation theorem, there exists $$g\in L^2_{r,q-1}(X,E)$$ such that $$\lambda (\alpha )=(g|\alpha )$$ for $$\alpha \in L^2_{r,q-1}(X,E)$$ with $$\Vert g\Vert ^2= \Vert \lambda \Vert ^2\le \int _X\psi ^{-1}|f|^2dv_X$$. We set $$\alpha =T^*s$$, $$s\in {{\,\mathrm{Dom}\,}}(T^*)$$. Thus $$(g|T^*s)=\lambda (T^*s)=(f|s)$$ and $$g\in {{\,\mathrm{Dom}\,}}(T^{**})={{\,\mathrm{Dom}\,}}(T)$$ satisfying $$Tg=f$$. $$\square $$

For certain complete CR manifold endowed with a Nakano *q*-semipositive line bundle, the $L^2$ method applies to solve the $\overline{\partial }_{b,E}$-equation for (*n*, *q*)-forms as follows. For the cohomology aspect of Nakano *q*-semipositive line bundles on complex manifolds, see [30].

#### Corollary 4.21

Let *X* be a CR manifold with a smooth locally free CR $\mathbb {R}$-action. Let $\Theta _X$ be an $\mathbb {R}$-invariant Hermitian metric on *X*. Assume $g_X$ is complete. Assume $\mathscr {L}=0$ and $d\Theta _X=0$. Let *E* be a ${\mathbb {R}}$-equivariant CR line bundle over *X* with a ${\mathbb {R}}$-invariant Hermitian metric $h^E$. Let $\lambda _1\le \cdots \le \lambda _n$ be eigenvalues of $R^E$ with respect to $\Theta _X$. Assume $(E,h^E)$ is Nakano *q*-semipositive with respect to $\Theta _X$, i.e., $\lambda _1+\cdots +\lambda _q\ge 0$. Then, for any $f\in L^2_{n,q}(X,E)$ satisfying $\overline{\partial }_{b,E} f=0$ and $\int _X (\lambda _1+\cdots +\lambda _q)^{-1}|f|^2dv_X< \infty$, there exists $g\in L^2_{n,q-1}(X,E)$ such that $\overline{\partial }_{b,E} g=f$ and $\Vert g\Vert ^2\le \int _X (\lambda _1+\cdots +\lambda _q)^{-1}|f|^2dv_X$.

### Vanishing Theorems

In this section, we present some vanishing theorems that follow from the previous $L^2$ estimates. We obtain first a CR counterpart of the Kodaira vanishing theorem [23, Theorem 1.5.4.(a)] as follows:

#### Corollary 4.22

Assume that $2\sqrt{-1}\mathscr {L}=\Theta _X$, $g_X$ is complete and let $\lambda <0$ and $1\le q\le n$. Then, we have

4.68 $$\begin{aligned} {{\,\mathrm{Ker}\,}}\square _b \cap \mathscr {E}(\lambda ,\sqrt{-1}T) \cap L^2_{n,q}(X)=0. \end{aligned}$$

This follows immediately from Theorem 4.18.

We obtain a CR counterpart of the Kodaira–Serre vanishing theorem [23, Theorem 1.5.6] as follows:

#### Corollary 4.23

Assume that $2\sqrt{-1}\mathscr {L}=\Theta _X$, $g_X$ is complete and let $C>0$ such that

4.69 $$\begin{aligned} \sqrt{-1}R^{K_X^*}\ge -C\Theta _X. \end{aligned}$$

Let $\lambda <-C$ and $1\le q\le n$. Then, we have

4.70 $$\begin{aligned} {{\,\mathrm{Ker}\,}}\square _b\cap \mathscr {E}(\lambda ,\sqrt{-1}T)\cap L^2_{0,q}(X)=0. \end{aligned}$$

This follows immediately from Theorem 4.10. We note that the previous vanishing theorems on CR manifolds imply the following generalizations due to Andreotti–Vesentini [2] of the Kodaira–Serre and Kodaira vanishing theorems for complete Kähler manifolds.

#### Corollary 4.24

(Andreotti–Vesentini) Let $(M,\omega )$ be a complete Kähler manifold of dimension *n* and let $(L,h^L)\rightarrow M$ be a Hermitian holomorphic line bundle such that $\sqrt{-1}R^L=\omega$ and there is $C>0$ such that $\sqrt{-1}R^{K^*_M}_{\omega }\ge -C\omega$ on *M*. Then there exists $m_0\in \mathbb {N}$ such that for every $m\ge m_0$ we have $H^q_{(2)}(M,L^m)=0$ for $q\ge 1$, where $H^q_{(2)}(M,L^m)$ denotes the $L^2$ *q*-th Dolbeault cohomology group with respect to the metric $(h^L)^m$ and volume form $\omega ^n/n!$ with values in $L^m$.

#### Proof

We apply the previous results for the CR manifold *X* constructed in Example 4.6. In this case $T=\partial _\theta$. For $m\in \mathbb {Z}$, the space $L^2_{0,q}(M,L^m)$ is isometric to the space of *m*-equivariant $L^2$ forms on *X*, $L^2_{0,q}(X)_m=\{u\in L^2_{0,q}(X): (e^{i\theta })^*u=e^{im\theta }u, \text {for any}\, e^{i\theta }\in S^{1}\}$. Note that $L^2_{0,q}(X)_m=\mathscr {E}^{(q)}(-m,\sqrt{-1}\partial _\theta )$ and the $L^2$-Dolbeault complex $(L^2_{0,\bullet }(M,L^m),\overline{\partial })$ is isomorphic to the $\overline{\partial }_b$-complex $(L^2_{0,\bullet }(X)_m,\overline{\partial }_b)$. Hence, the assertion follows from Theorem 4.10. $\square$

In the same vein recover from Theorem 4.18 the following vanishing theorem for the $L^2$-cohomology of positive bundles twisted with the canonical bundle on complete Kähler manifolds.

#### Corollary 4.25

(Andreotti–Vesentini) Let $(M,\omega )$ be a complete Kähler manifold of dimension *n* and let $(L,h^L)\rightarrow M$ be a Hermitian holomorphic line bundle such that $\sqrt{-1}R^L=\omega$. Then $H^{n,q}_{(2)}(M,L)=0$ for $q\ge 1$, where $H^{n,q}_{(2)}(M,L)$ denotes the $L^2$ *q*-th Dolbeault cohomology group with respect to the metric $h^L$ and volume form $\omega ^n/n!$ with values in $K_X\otimes L$.

### Szegö Kernel Asymptotic Expansions

In this section, we prove Theorem 1.2 and Corollary 1.4. We first introduced some notations. Let $D\subset X$ be an open coordinate patch with local coordinates $x=(x_1,\ldots ,x_{2n+1})$. Let $m\in {\mathbb {R}}$, $0\le \rho ,\delta \le 1$. Let $S^m_{\rho ,\delta }(T^*D)$ denote the Hörmander symbol space on $T^*D$ of order *m* type $(\rho ,\delta )$ and let $S^m_{\mathrm{cl\,}}(T^*D)$ denote the space of classical symbols on $T^*D$ of order *m*, see Grigis–Sjöstrand [11, Definition 1.1 and p. 35] and Definition 2.1. Let $L^m_{\rho ,\delta }(D)$ and $L^m_{\mathrm{cl\,}}(D)$ denote the space of pseudodifferential operators on *D* of order *m* type $(\rho ,\delta )$ and the space of classical pseudodifferential operators on *D* of order *m*, respectively.

Let $\Sigma$ be the characteristic manifold of $\Box _b$. We have

4.71 $$\begin{aligned} \begin{aligned} \Sigma&=\Sigma ^-\cup \Sigma ^+,\\ \Sigma ^-&=\{(x,-c\omega _0(x))\in T^*X;\, c<0\},\\ \Sigma ^+&=\{(x,-c\omega _0(x))\in T^*X;\, c>0\}. \end{aligned} \end{aligned}$$

We recall the following definition introduced in [19, Definition 2.4].

#### Definition 4.26

Let $Q:L^2(X)\rightarrow L^2(X)$ be a continuous operator. Let $D\Subset X$ be an open local coordinate patch of *X* with local coordinates $x=(x_1,\ldots ,x_{2n+1})$ and let $\eta =(\eta _1,\ldots ,\eta _{2n+1})$ be the dual variables of *x*. We write

$$\begin{aligned} Q\equiv 0 \text {at}\, \Sigma ^-\cap T^*D\,, \end{aligned}$$

if for every $D'\Subset D$,

$$\begin{aligned} Q(x,y)\equiv \int e^{i\langle x-y,\eta \rangle }q(x,\eta )d\eta \,\,\text {on}\, D', \end{aligned}$$

where $q(x,\eta )\in S^0_{1,0}(T^*D')$ and there exist $M>0$ and a conic open neighborhood $\Lambda _-$ of $\Sigma ^-$ such that for every $(x,\eta )\in T^*D'\cap \Lambda _-$ with $|\eta |\ge M$, we have $q(x,\eta )=0$.

For a given point $x_0\in D$, let $\{W_j\}_{j=1}^{n}$ be an orthonormal frame of $T^{(1,0)}X$ with respect to $\langle \,\cdot \,|\,\cdot \,\rangle$ near $x_0$, for which the Levi form is diagonal at $x_0$. Put

4.72 $$\begin{aligned} \mathscr {L}_{x_0}(W_j,\overline{W}_\ell )=\mu _j(x_0)\delta _{j\ell }\,,\;\; j,\ell =1,\ldots ,n\,. \end{aligned}$$

We will denote by

4.73 $$\begin{aligned} \det \mathscr {L}_{x_0}=\prod _{j=1}^{n}\mu _j(x_0)\,. \end{aligned}$$

We recall the following results in [19, Theorems 1.9, 5.1].

#### Theorem 4.27

Let $D\Subset X$ be an open coordinate patch with local coordinates $x=(x_1,\ldots ,x_{2n+1})$. Let $Q:L^2(X)\rightarrow L^2(X)$ be a continuous operator and let $Q^*$ be the $L^2$ adjoint of *Q* with respect to $(\,\cdot \,|\,\cdot \,)$. Suppose that $\Box ^{(0)}_b$ has local $L^2$ closed range on *D* with respect to *Q* and $QS^{(0)}=S^{(0)}Q$ on $L^2(X)$ and

$$\begin{aligned} Q-Q_0\equiv 0\quad \text {at}\, \Sigma ^-\cap T^*D, \end{aligned}$$

where $$Q_0\in L^0_{\mathrm{cl\,}}(D)$$. Then,

4.74 $$\begin{aligned} (Q^*S^{(0)}Q)(x,y)\equiv \int ^\infty _0e^{i\varphi (x,y)t}a(x,y,t)dt\ \ \text {on}\, D, \end{aligned}$$

where

4.75 $$\begin{aligned} \begin{aligned}&\varphi \in \mathscr {C}^\infty (D\times D),\ \ \mathrm{Im\,}\varphi (x, y)\ge 0,\\&\varphi (x, x)=0,\ \ \varphi (x, y)\ne 0\ \ \text {if}\ \ x\ne y,\\&d_x\varphi (x, y)\big |_{x=y}=\omega _0(x), \ \ d_y\varphi (x, y)\big |_{x=y}=-\omega _0(x), \\&\varphi (x, y)=-\overline{\varphi }(y, x), \end{aligned} \end{aligned}$$

$a(x, y, t)\in S^{n}_{\mathrm{cl\,}}\big (D\times D\times {\mathbb {R}}_+\big )$ and the leading term $a_0(x,y)$ of the expansion (2.4) of *a*(*x*, *y*, *t*) satisfies

4.76 $$\begin{aligned} a_0(x, x)=\frac{1}{2}\pi ^{-n-1} |\mathrm{det\,}{\mathcal {L}}_x|\overline{q(x,\omega _0(x))}q(x,\omega _0(x)),\ \ \text {for all}\, x\in D, \end{aligned}$$

where $\det {\mathcal {L}}_x$ is the determinant of the Levi form defined in (4.73), $q(x,\eta )\in \mathscr {C}^\infty (T^*D)$ is the principal symbol of *Q*.

We refer the reader to [19, Theorems 3.3, 4.4] for more properties for the phase $\varphi$ in (4.75). Let $D=U\times {\mathcal {I}}$ be a BRT chart with BRT coordinates $x=(x_1,\ldots ,x_{2n+1})$. For $\lambda \in {\mathbb {R}}$, put

4.77 $$\begin{aligned} {\hat{Q}}_{\tau _\lambda }:=(2\pi )^{-(2n+1)}\int e^{i\langle x-y,\eta \rangle } \tau _\lambda (-\eta _{2n+1})d\eta \in L^0_{1,0}(D), \end{aligned}$$

where $\tau _\lambda \in \mathscr {C}^\infty ({\mathbb {R}})$ is as in the discussion before Theorem 4.14. It is not difficult to see that

4.78 $$\begin{aligned} {\hat{Q}}_{\tau _\lambda }-I\equiv 0\ \ \text {at}\, \Sigma ^-\bigcap T^*D. \end{aligned}$$

Assume that the ${\mathbb {R}}$-action is free. From (4.11), we see that $Q_{\tau _\lambda } ={\hat{Q}}_{\tau _\lambda }$ on *D*. From this observation, Theorems 4.14, 4.27, (4.78), and noticing that $Q^*_{\tau _\lambda }S^{(0)}Q_{\tau _\lambda }= Q_{\tau ^2_\lambda }S^{(0)}$, where $Q^*_{\tau _\lambda }$ is the $L^2$ adjoint of $Q_{\tau _\lambda }$ with respect to $(\,\cdot \,|\,\cdot \,)$, we get

#### Theorem 4.28

Suppose that the ${\mathbb {R}}$-action is free. Assume that $g_\mathscr {L}$ is complete and there is $C>0$ such that

$$\begin{aligned} \sqrt{-1}R^{K^*_X}_{\mathscr {L}}\ge -2C\sqrt{-1}\mathscr {L},\ \ {(2\sqrt{-1}\mathscr {L})^n\wedge \omega _0\ge C\Theta _X^n\wedge \omega _0.} \end{aligned}$$

Let $D=U\times {\mathcal {I}}\Subset X$ be a BRT chart with BRT coordinates $x=(x_1,\ldots ,x_{2n+1})$. Let $\lambda \in {\mathbb {R}}$, $\lambda <-C$. Then,

4.79 $$\begin{aligned} (Q_{\tau ^2_\lambda }S^{(0)})(x,y)\equiv \int ^\infty _0e^{i\varphi (x,y)t}s(x,y,t)dt\ \ \text {on}\, D, \end{aligned}$$

where $\varphi \in \mathscr {C}^\infty (D\times D)$ is as in (4.74), $s(x, y, t)\in S^{n}_{\mathrm{cl\,}}\big (D\times D\times {\mathbb {R}}_+\big )$ and the leading term $s_0(x,y)$ of the expansion (2.4) of *s*(*x*, *y*, *t*) satisfies

4.80 $$\begin{aligned} s_0(x, x)=\frac{1}{2}\pi ^{-n-1} |\mathrm{det\,}{\mathcal {L}}_x|,\ \ \text {for all}\, x\in D. \end{aligned}$$

We now assume that the ${\mathbb {R}}$-action is not free. From (4.8), we know that the ${\mathbb {R}}$-action comes from a CR torus action $\mathbb T^d=(e^{i\theta _1},\ldots ,e^{i\theta _d})$ on *X* and $\omega _0$, $\Theta _X$ are ${\mathbb {T}}^d$ invariant. We will use the same notations as in the discussion before Proposition 4.8. We need

#### Lemma 4.29

Suppose that the ${\mathbb {R}}$-action is not free. With the notations and assumptions used above, let $D=U\times {\mathcal {I}}\Subset X$ be a BRT chart with BRT coordinates $x=(x',x_{2n+1})$, $x'=(x_1,\ldots ,x_{2n})$. Fix $D_0\Subset D$ and $\lambda \in {\mathbb {R}}$. For $u\in \mathscr {C}^\infty _c(D_0)$, we have

4.81 $$\begin{aligned} \begin{aligned}&Q_{\tau _\lambda }u={\hat{Q}}_{\tau _\lambda }u+{\hat{R}}_{\tau _\lambda }u\ \ \text {on}\, D_0,\\&({\hat{R}}_{\tau _\lambda }u)(x)=\frac{1}{2\pi }\sum _{(m_1,\ldots ,m_d)\in {\mathbb {Z}}^d}\, \int \limits _{{\mathbb {T}}^d}e^{i\langle x_{2n+1}-y_{2n+1}, \eta _{2n+1}\rangle +i(\sum ^d_{j=1}m_j\beta _j)y_{2n+1}-im_1\theta _1- \ldots -im_d\theta _d}\\&\times \tau _\lambda (-\eta _{2n+1}) (1-\chi (y_{2n+1}))u((e^{i\theta _1}, \ldots ,e^{i\theta _d})\circ x')d{\mathbb {T}}_dd\eta _{2n+1}dy_{2n+1}\ \ \text {on}\, D_0, \end{aligned} \end{aligned}$$

where $\chi \in \mathscr {C}^\infty _c(I)$, $\chi (x_{2n+1})=1$ for every $(x',x_{2n+1})\in D_0$ and $\beta _1\in {\mathbb {R}},\ldots ,\beta _d\in {\mathbb {R}}$ are as in (4.17).

#### Proof

We also write $y=(y',y_{2n+1})=(y_1,\ldots ,y_{2n+1})$, $y'=(y_1,\ldots ,y_{2n})$, to denote the BRT coordinates *x*. Let $u\in \mathscr {C}^\infty _c(D_0)$. From (4.19), it is easy to see that on *D*,

4.82 $$\begin{aligned} \begin{aligned}&Q_{\tau _\lambda }u(y) =\sum _{(m_1,\ldots ,m_d)\in {\mathbb {Z}}^d}\tau _\lambda (-\sum ^d_{j=1}m_j\beta _j)e^{i(\sum ^d_{j=1}m_j\beta _j) y_{2n+1}}\times \\&\quad \int _{{\mathbb {T}}^d}e^{-(im_1\theta _1+\ldots +im_d\theta _d)} u((e^{i\theta _1},\ldots ,e^{i\theta _d})\circ y')dT_d. \end{aligned} \end{aligned}$$

Now, we claim that

4.83 $$\begin{aligned} {\hat{Q}}_{\tau _\lambda }+{\hat{R}}_{\tau _\lambda } =Q_{\tau _\lambda }\ \ \text {on}\, \mathscr {C}^\infty _0(D_0). \end{aligned}$$

Let $u\in \mathscr {C}^\infty _c(D_0)$. From Fourier inversion formula, it is straightforward to see that

4.84 $$\begin{aligned} \begin{aligned}&{\hat{Q}}_{\tau _\lambda }u(x) =\frac{1}{2\pi }\sum _{(m_1,\ldots ,m_d)\in {\mathbb {Z}}^d} \int e^{i\langle x_{2n+1}-y_{2n+1},\eta _{2n+1}\rangle } \tau _\lambda (-\eta _{2n+1})\chi (y_{2n+1})\\&\quad \times e^{i(\sum ^d_{j=1}m_j\beta _j)y_{2n+1}- im_1\theta _1-\ldots -im_d\theta _d}u((e^{i\theta _1}, \ldots ,e^{i\theta _d})\circ x')d{\mathbb {T}}_ddy_{2n+1}d\eta _{2n+1}. \end{aligned} \end{aligned}$$

From (4.84) and the definition of ${\hat{R}}_{\tau _\lambda }$, we have

4.85 $$\begin{aligned} \begin{aligned}&({\hat{Q}}_{\tau _\lambda }+{\hat{R}}_{\tau _\lambda })u(x)\\&=\frac{1}{2\pi }\sum _{(m_1,\ldots ,m_d)\in {\mathbb {Z}}^d} \int e^{i\langle x_{2n+1}-y_{2n+1},\eta _{2n+1}\rangle } \tau _\lambda (-\eta _{2n+1})\\&\quad \times e^{i(\sum ^d_{j=1}m_j\beta _j)y_{2n+1}-im_1\theta _1- \ldots -im_d\theta _d}u((e^{i\theta _1}, \ldots ,e^{i\theta _d})\circ x')d{\mathbb {T}}_ddy_{2n+1}d\eta _{2n+1}. \end{aligned} \end{aligned}$$

Note that the following formula holds for every $\alpha \in {\mathbb {R}}$,

4.86 $$\begin{aligned} \int e^{i\alpha y_{2n+1}}e^{-iy_{2n+1}\eta _{2n+1}}dy_{2n+1} =2\pi \delta _\alpha (\eta _{2n+1}), \end{aligned}$$

where the integral is defined as an oscillatory integral and $\delta _\alpha$ is the Dirac measure at $\alpha$. Using (4.82), (4.86), and the Fourier inversion formula, (4.85) becomes

4.87 $$\begin{aligned} \begin{aligned} ({\hat{Q}}_{\tau _\lambda }+{\hat{R}}_{\tau _\lambda })u(x) =&\sum _{(m_1,\ldots ,m_d)\in {\mathbb {Z}}^d} \tau _\lambda (-\sum ^d_{j=1}m_j\beta _j) e^{i(\sum ^d_{j=1}m_j\beta _j)x_{2n+1}}\times \\&\int _{{\mathbb {T}}_d}e^{-im_1\theta _1-\ldots - im_d\theta _d}u((e^{i\theta _1},\ldots ,e^{i\theta _d})\circ x')d{\mathbb {T}}_d\\ =&Q_{\tau _\lambda }u(x). \end{aligned} \end{aligned}$$

From (4.87), the claim (4.83) follows. $\square$

To study $Q_{\tau ^2_\lambda }S^{(0)}$ when the ${\mathbb {R}}$ is not free, we also need the following two known results [19, Theorems 3.2, 5.2].

#### Theorem 4.30

We assume that the ${\mathbb {R}}$-action is arbitrary. Let $D\Subset X$ be a coordinate patch with local coordinates $x=(x_1,\ldots ,x_{2n+1})$. Then there exist properly supported continuous operators $A\in L^{-1}_{\frac{1}{2},\frac{1}{2}}(D)$ , ${\tilde{S}}\in L^{0}_{\frac{1}{2},\frac{1}{2}}(D)$, such that

4.88 $$\begin{aligned} \begin{aligned}&\Box ^{(0)}_bA+{\tilde{S}}=I \text {on}\, D,\\&A^*\Box ^{(0)}_b+{\tilde{S}}=I \text {on}\, D,\\&\Box ^{(0)}_b{\tilde{S}}\equiv 0\ \ \text {on}\, D,\\&A\equiv A^*\ \ \text {on}\, D,\ \ {\tilde{S}}A\equiv 0\ \ \text {on}\, D,\\&{\tilde{S}}\equiv {\tilde{S}}^*\equiv {\tilde{S}}^2\ \ \text {on}\, D, \end{aligned} \end{aligned}$$

where $A^*$, ${\tilde{S}}^*$ are the formal adjoints of *A*, ${\tilde{S}}$ with respect to $(\,\cdot \,|\,\cdot \,)$, respectively, and ${\tilde{S}}(x,y)$ satisfies

4.89 $$\begin{aligned} {\tilde{S}}(x, y)\equiv \int ^{\infty }_{0}e^{i\varphi (x, y)t}s(x, y, t)dt\ \ \text {on}\, D, \end{aligned}$$

where $\varphi (x,y)\in \mathscr {C}^\infty (D\times D)$ and $s(x, y, t)\in S^{n}_{\mathrm{cl\,}}(D\times D\times {\mathbb {R}}_+)$ are as in (4.79).

#### Theorem 4.31

Let us consider an arbitrary ${\mathbb {R}}$-action and let $Q:L^2(X)\rightarrow L^2(X)$ be a continuous operator and let $Q^*$ be the $L^2$ adjoint of *Q* with respect to $(\,\cdot \,|\,\cdot \,)$. Suppose that $\Box ^{(0)}_b$ has local $L^2$ closed range on *D* with respect to *Q* and $QS^{(0)}=S^{(0)}Q$ on $L^2(X)$. Let *D* be a coordinate patch with local coordinates $x=(x_1,\ldots ,x_{2n+1})$. We have

4.90 $$\begin{aligned} Q^*S^{(0)}Q\equiv {\tilde{S}}^*Q^*Q{\tilde{S}} \text {on}\, D, \end{aligned}$$

where ${\tilde{S}}$ is as in Theorem 4.30.

For the proof we need the following.

#### Lemma 4.32

Suppose that the ${\mathbb {R}}$-action is not free. Fix $p\in X$. Let $D=U\times {\mathcal {I}}$ be a BRT chart defined near *p* with BRT coordinates $x=(x',x_{2n+1})$, $x'=(x_1,\ldots ,x_{2n})$, $x(p)=0$. Fix $D_0\Subset D$, $p\in D_0$, and $\lambda \in {\mathbb {R}}$. Then,

$$\begin{aligned} {\tilde{S}}{\hat{R}}_{\tau _\lambda }\equiv 0\ \ \text {on}\, D_0, \end{aligned}$$

where ${\hat{R}}_{\tau _\lambda }$ and ${\tilde{S}}$ are as in Lemma 4.29 and Theorem 4.30, respectively.

#### Proof

From (4.75), we may assume $D_0$ is small so that

4.91 $$\begin{aligned} |\partial _{y_{2n+1}}\varphi (x,y)|\ge C, \text {for every}\, (x,y)\in D_0, \end{aligned}$$

where $C>0$ is a constant. Let $g\in \mathscr {C}^\infty _c(D_0)$. From (4.81) and (4.89), we have

4.92 $$\begin{aligned} \begin{aligned}&({\tilde{S}}{\hat{R}}_{\tau _\lambda }g)(x)\\&=\frac{1}{2\pi }\sum _{(m_1,\ldots ,m_d)\in {\mathbb {Z}}^d}\, \int e^{it\varphi (x,u)}a(x,u,t)e^{i\langle u_{2n+1}-y_{2n+1}, \eta _{2n+1}\rangle +i(\sum ^d_{j=1}m_j\beta _j)y_{2n+1}-i\sum ^d_{j=1}m_j\theta _j}\\&\times \tau _\lambda (-\eta _{2n+1}) (1-\chi (y_{2n+1})) g((e^{i\theta _1},\ldots ,e^{i\theta _d})\circ u')d{\mathbb {T}}_dd \eta _{2n+1}dy_{2n+1}dv_X(u)\ \ \text {on}\, D_0, \end{aligned} \end{aligned}$$

where we also write $u=(u',u_{2n+1})$, $u'=(u_1,\ldots ,u_{2n})$, to denote the BRT coordinates *x*. Since $u_{2n+1}\ne y_{2n+1}$, for every $(u',u_{2n+1})\in D_0$, $y_{2n+1}\in \mathrm{Supp\,}(1-\chi (y_{2n+1}))$, we can integrate by parts in $\eta _{2n+1}$ and rewrite (4.92):

4.93 $$\begin{aligned} \begin{aligned}&({\tilde{S}}{\hat{R}}_{\tau _\lambda }g)(x)\\&=\frac{1}{2\pi }\sum _{(m_1,\ldots ,m_d)\in {\mathbb {Z}}^d}\, \int e^{it\varphi (x,u)}a(x,u,t)\frac{1}{i(u_{2n+1}-y_{2n+1})}\\&\times e^{i\langle u_{2n+1}-y_{2n+1},\eta _{2n+1}\rangle + i(\sum ^d_{j=1}m_j\beta _j)y_{2n+1}-i\sum ^d_{j=1}m_j\theta _j}\\&\times \tau '_\lambda (-\eta _{2n+1}) (1-\chi (y_{2n+1})) g((e^{i\theta _1},\ldots ,e^{i\theta _d})\circ u') d{\mathbb {T}}_dd\eta _{2n+1}dy_{2n+1}dv_X(u)\ \ \text {on}\, D_0. \end{aligned} \end{aligned}$$

Let

$$\begin{aligned}\begin{aligned}&A(x,u',y_{2n+1})\\&:=\int e^{it\varphi (x,u)+i\langle u_{2n+1}-y_{2n+1}, \eta _{2n+1}\rangle }a(x,u,t)(1-\chi (y_{2n+1})) \frac{1}{i(u_{2n+1}-y_{2n+1})}\\&\quad \quad \times \tau '_\lambda (-\eta _{2n+1})d\eta _{2n+1} du_{2n+1}dt.\end{aligned} \end{aligned}$$

By (4.91) there exists $c>0$ such that

$$\begin{aligned}&|\partial _{u_{2n+1}}(it\varphi (x,u)+ i\langle u_{2n+1}-y_{2n+1},\eta _{2n+1}\rangle )|\ge ct,\ \\&\quad \text {for}\, t\gg |\lambda |, \eta _{2n+1}\in \mathrm{Supp\,}\tau '_\lambda (-\eta _{2n+1}) \end{aligned}$$

for every $(x,u)\in D_0\times D_0$. Hence, we can integrate by parts in $u_{2n+1}$ and $\eta _{2n+1}$ and deduce that

4.94 $$\begin{aligned} A(x,u',y_{2n+1})\in \mathscr {C}^\infty (D_0\times D_0\times {\mathbb {R}}) \text {and}\, A(x,u',y_{2n+1}) \text {is a Schwartz function in}\, y_{2n+1}. \end{aligned}$$

We have

4.95 $$\begin{aligned} \begin{aligned}&({\tilde{S}}{\hat{R}}_{\tau _\lambda }g)(x)\\&=\frac{1}{2\pi }\sum _{(m_1,\ldots ,m_d)\in {\mathbb {Z}}^d}\int A(x,u',y_{2n+1}) e^{i(\sum ^d_{j=1}m_j\beta _j)y_{2n+1}-im_1\theta _1-\ldots -im_d\theta _d}\\&\times g((e^{i\theta _1},\ldots ,e^{i\theta _d})\circ u')dv(u')dy_{2n+1}d{\mathbb {T}}_d, \end{aligned} \end{aligned}$$

where $dv_X(u)=dv(u')du_{2n+1}$. From (4.94) and (4.95), we have

4.96 $$\begin{aligned} \begin{aligned} \Vert {\tilde{S}}{\hat{R}}_{\tau _\lambda }g\Vert _{D_0,s}&\le C\sum _{(m_1,\ldots ,m_d)\in {\mathbb {Z}}^d} \int \Big |\int _{{\mathbb {T}}^d}e^{-im_1\theta _1-\ldots -im_d\theta _d} g((e^{i\theta _1},\ldots ,e^{i\theta _d}\circ u')\Big |^2\tau (u)dv_X(u)\\&= C\sum _{(m_1,\ldots ,m_d)\in {\mathbb {Z}}^d} \int \Big |\int _{{\mathbb {T}}^d}e^{-im_1\theta _1-\ldots -im_d\theta _d} g((e^{i\theta _1},\ldots ,e^{i\theta _d}\circ u)\Big |^2\tau (u)dv_X(u)\\&\le {\hat{C}}\sum _{(m_1,\ldots ,m_d)\in {\mathbb {Z}}^d} \int \Big |\int _{{\mathbb {T}}^d}e^{-im_1\theta _1-\ldots -im_d\theta _d} g((e^{i\theta _1},\ldots ,e^{i\theta _d}\circ u)\Big |^2dv_X(u)\\&\le {\hat{C}}_0\Vert g\Vert ^2, \end{aligned} \end{aligned}$$

where $\Vert \cdot \Vert _{D_0,s}$ denotes the standard Sobolev norm of order *s* on $D_0$, $C, {\hat{C}}, {\hat{C}}_0>0$ are constants, $\tau \in \mathscr {C}^\infty _0(D)$, $\tau =1$ near $D_0$. From (4.96), we deduce that

$$\begin{aligned} {\tilde{S}}{\hat{R}}_{\tau _\lambda }: L^2_c(D_0)\rightarrow H^s_{\mathrm{loc\,}}(D_0) \text {is continuous, for every}\, s\in {\mathbb {N}}. \end{aligned}$$

Let $\triangle _X: \mathscr {C}^\infty (X)\rightarrow \mathscr {C}^\infty (X)$ be the standard Laplacian on *X* induced by $\langle \,\cdot \,|\,\cdot \,\rangle$. Since $\langle \,\cdot \,|\,\cdot \,\rangle$ is ${\mathbb {T}}^d$ invariant, $\triangle _X$ is ${\mathbb {T}}^d$ invariant. Fix $s\in {\mathbb {N}}$. Let

$$\begin{aligned} G_s: \mathscr {C}^\infty _c(D_0)\rightarrow \mathscr {C}^\infty _c(D_0) \end{aligned}$$

be a parametrix of $\triangle _X^s$ on $D_0$ and $G_s$ is properly supported on $D_0$. Hence, there is a properly supported smoothing operator

$$\begin{aligned} F_s: {\mathscr {E}}'(D_0)\rightarrow \mathscr {C}^\infty _c(D_0) \end{aligned}$$

such that

4.97 $$\begin{aligned} g=(\triangle ^s_XG_s+F_s)g \ \text {on}\, D_0, \end{aligned}$$

for all $g\in \mathscr {C}^\infty _c(D_0)$. Now, on $D_0$,

4.98 $$\begin{aligned} {\tilde{S}}{\hat{R}}_{\tau _\lambda }g={\tilde{S}}{\hat{R}}_{\tau _\lambda }(\triangle ^s_XG_sg) +{\tilde{S}}{\hat{R}}_{\tau _\lambda }(F_sg). \end{aligned}$$

Since $F_s$ is smoothing, we have

4.99 $$\begin{aligned} \Vert {\tilde{S}}{\hat{R}}_{\tau _\lambda }(F_sg)\Vert _{D_0,s}\le C\Vert g\Vert _{-s}, \end{aligned}$$

where $C>0$ is a constant. Now, we can integrate by parts and repeat the proof of (4.95) and show that

4.100 $$\begin{aligned} \begin{aligned}&({\tilde{S}}{\hat{R}}_{\tau _\lambda }\triangle ^s_XG_sg)(x)\\&=\frac{1}{2\pi }\sum _{(m_1,\ldots ,m_d)\in {\mathbb {Z}}^d}\int A_s(x,u',y_{2n+1}) e^{i(\sum ^d_{j=1}m_j\beta _j)y_{2n+1}-im_1\theta _1-\ldots -im_d\theta _d}\\&\times (G_sg)((e^{i\theta _1},\ldots ,e^{i\theta _d})\circ u')dv(u')dy_{2n+1}d{\mathbb {T}}_d, \end{aligned} \end{aligned}$$

where

4.101 $$\begin{aligned} A_s(x,u',y_{2n+1})\in \mathscr {C}^\infty (D_0\times D_0\times {\mathbb {R}}) \text {is a Schwartz function in}\, y_{2n+1}. \end{aligned}$$

From (4.100), (4.101), and noticing that $G_s: H^{-2s}_{\mathrm{comp\,}}(D)\rightarrow H^0_{\mathrm{comp\,}}(D)$ is continuous, we can repeat the proof of (4.96) and conclude that

4.102 $$\begin{aligned} \Vert {\tilde{S}}{\hat{R}}_{\tau _\lambda }(\triangle ^s_XG_sg)\Vert _{D_0,s}\le C\Vert G_sg\Vert \le C_1\Vert g\Vert _{-s}, \end{aligned}$$

where $C, C_1>0$ are constants. From (4.97), (4.98), (4.99), and (4.102), we get that

$$\begin{aligned} {\tilde{S}}{\hat{R}}_{\tau _\lambda }: H^{-s}_{\mathrm{comp\,}}(D_0)\rightarrow H^s_{\mathrm{loc\,}}(D_0) \text {is continuous for every}\, s\in {\mathbb {N}}. \end{aligned}$$

Hence, ${\tilde{S}}{\hat{R}}_{\tau _\lambda }$ is smoothing on $D_0$. $\square$

#### Theorem 4.33

Suppose that the ${\mathbb {R}}$-action is not free. Assume that $g_\mathscr {L}$ is complete and there is $C>0$ such that

$$\begin{aligned} \sqrt{-1}R^{K^*_X}_{\mathscr {L}}\ge -2C\sqrt{-1}\mathscr {L},\ \ {(2\sqrt{-1}\mathscr {L})^n\wedge \omega _0\ge C\Theta _X^n\wedge \omega _0.} \end{aligned}$$

Let $D=U\times {\mathcal {I}}\Subset X$ be a BRT chart with BRT coordinates $x=(x_1,\ldots ,x_{2n+1})$. Let $\lambda \in {\mathbb {R}}$, $\lambda <-C$. Then,

4.103 $$\begin{aligned} (Q_{\tau ^2_\lambda }S^{(0)})(x,y)\equiv \int ^\infty _0e^{i\varphi (x,y)t}s(x,y,t)dt\ \ \text {on}\, D, \end{aligned}$$

where $\varphi \in \mathscr {C}^\infty (D\times D)$ and $s(x, y, t)\in S^{n}_{\mathrm{cl\,}}\big (D\times D\times {\mathbb {R}}_+\big )$ are as in (4.79).

#### Proof

From (4.81), (4.90), and Lemma 4.32, we see that on *D*,

$$\begin{aligned} Q_{\tau ^2_\lambda }S^{(0)}\equiv {\tilde{S}}^*{\hat{Q}}_{\tau _\lambda }^*{\hat{Q}}_{\tau _\lambda }{\tilde{S}}. \end{aligned}$$

Using this observation, we can repeat the proof of [19, Theorem 5.8] and obtain the conclusion. $\square$

#### Theorem 4.34

Let us consider an arbitrary ${\mathbb {R}}$-action. Assume that $g_\mathscr {L}$ is complete and there is $C>0$ such that

$$\begin{aligned} \sqrt{-1}R^{K^*_X}_{\mathscr {L}}\ge -2C\sqrt{-1}\mathscr {L},\ \ {(2\sqrt{-1}\mathscr {L})^n\wedge \omega _0\ge C\Theta _X^n\wedge \omega _0.} \end{aligned}$$

Let $D=U\times I$ be a BRT chart with BRT coordinates $x=(x_1,\ldots ,x_{2n+1})$. Let $\lambda \in {\mathbb {R}}$, $\lambda <-C$. Then,

4.104 $$\begin{aligned} (I-Q_{\tau ^2_\lambda })^2S^{(0)}\equiv 0\ \ \text {on}\, D. \end{aligned}$$

#### Proof

From (4.88), we have

4.105 $$\begin{aligned} (I-Q_{\tau ^2_\lambda })S^{(0)}={\tilde{S}}(I-Q_{\tau ^2_\lambda })S^{(0)}. \end{aligned}$$

From Lemma 4.32, we have

$$\begin{aligned} {\tilde{S}}(I-Q_{\tau ^2_\lambda })\equiv {\tilde{S}}(I-{\hat{Q}}_{\tau ^2_\lambda })\ \ \text {on}\, D_0. \end{aligned}$$

Since $\mathrm{WF\,}(I-{\hat{Q}}_{\tau ^2_\lambda })\bigcap \Sigma ^- =\emptyset$ and $\mathrm{WF'\,}({\tilde{S}})=\mathrm{diag\,}(\Sigma ^-\times \Sigma ^-)$, we have

4.106 $$\begin{aligned} {\tilde{S}}(I-{\hat{Q}}_{\tau ^2_\lambda })\equiv 0\ \ \text {on}\, D_0, \end{aligned}$$

where $\mathrm{WF\,}(I-{\hat{Q}}_{\tau ^2_\lambda })$ denotes the wave front set of $I-{\hat{Q}}_{\tau ^2_\lambda }$ and

$$\begin{aligned} \mathrm{WF'\,}({\tilde{S}})=\{(x,\xi ,y,\eta )\in T^*D\times T^*D;\, (x,\xi ,y,-\eta ) \in \mathrm{WF\,}({\tilde{S}}). \end{aligned}$$

From (4.105) and (4.106), we get

4.107 $$\begin{aligned} (I-Q_{\tau ^2_\lambda })S^{(0)}:L^2(X)\rightarrow \mathscr {C}^\infty (D) \text {is continuous} \end{aligned}$$

and hence

4.108 $$\begin{aligned} S^{(0)}(I-Q_{\tau ^2_\lambda }):{\mathscr {E}}'(D)\rightarrow L^2(X) \text {is continuous}. \end{aligned}$$

From (4.107) and (4.108), we get

$$\begin{aligned} (I-Q_{\tau ^2_\lambda })S^{(0)}(I-Q_{\tau ^2_\lambda }): {\mathscr {E}}'(D)\rightarrow \mathscr {C}^\infty (D) \text {is continuous}. \end{aligned}$$

The theorem follows. $\square$

We can now prove the main result of this work.

#### Theorem 4.35

(=Theorem 1.2) Let the ${\mathbb {R}}$-action be arbitrary. Assume that $g_\mathscr {L}$ is complete and there is $C>0$ such that

$$\begin{aligned} \sqrt{-1}R^{K^*_X}_{\mathscr {L}}\ge -2C\sqrt{-1}\mathscr {L},\ \ {(2\sqrt{-1}\mathscr {L})^n\wedge \omega _0\ge C\Theta _X^n\wedge \omega _0.} \end{aligned}$$

Let $D\Subset X$ be a local coordinate patch with local coordinates $x=(x_1,\ldots ,x_{2n+1})$. Then,

4.109 $$\begin{aligned} S^{(0)}(x,y)\equiv \int ^\infty _0e^{i\varphi (x,y)t}s(x,y,t)dt\ \ \text {on}\, D, \end{aligned}$$

where $\varphi \in \mathscr {C}^\infty (D\times D)$ satisfies (4.75), $s(x, y, t)\in S^{n}_{\mathrm{cl\,}}\big (D\times D\times {\mathbb {R}}_+\big )$ and the leading term $s_0(x,y)$ of the expansion (2.4) of *s*(*x*, *y*, *t*) satisfies

4.110 $$\begin{aligned} s_0(x, x)=\frac{1}{2}\pi ^{-n-1} |\mathrm{det\,}{\mathcal {L}}_x|,\ \ \text {for all}\, x\in D. \end{aligned}$$

#### Proof

Let $\lambda \in {\mathbb {R}}$, $\lambda <-C$. From Theorem 4.34, we have

4.111 $$\begin{aligned} (I-2Q_{\tau ^2_\lambda }+Q_{\tau ^4_\lambda })S^{(0)}\equiv 0\ \ \text {on}\, D. \end{aligned}$$

We can repeat the proofs of Theorems 4.28 and 4.33 and get

4.112 $$\begin{aligned} \begin{aligned}&Q_{\tau ^2_\lambda }S^{(0)}\equiv \int ^\infty _0e^{i\varphi (x,y)t} {\hat{s}}(x,y,t)\ \ \text {on}\, D,\\&Q_{\tau ^4_\lambda }S^{(0)}\equiv \int ^\infty _0e^{i\varphi (x,y)t} {\tilde{s}}(x,y,t)\ \ \text {on}\, D, \end{aligned} \end{aligned}$$

where $\varphi \in \mathscr {C}^\infty (D\times D)$, ${\hat{s}}(x, y, t), {\tilde{s}}(x,y,t)\in S^{n}_{\mathrm{cl\,}} \big (D\times D\times {\mathbb {R}}_+\big )$ are as in (4.79). From (4.111) and (4.112), the theorem follows. $\square$

#### Proof of Theorem 1.3

The proof is analogous to the proof of Theorem 1.2 by using Theorem 4.15 instead of Theorem 4.13. $\square$

#### Proof of Corollary 1.4

Let *K* be a compact set of *X*. Fix $x\in K$. From Theorem 1.2 and the fact that the Szegő kernel is smoothing away the diagonal, we can repeat the proof of [19, Theorem 1.10] and deduce that there are open neighborhoods $V_x\subset U_x$ of *x* and global smooth $L^2$ CR functions $(f_{0,x}, f_{1,x},\cdots ,f_{N_x,x} )=F_x$ such that $F_x: U_x\rightarrow \mathbb {C}^{N_x+1}$ is an embedding and $\sup _{K\setminus U_x}|f_{0,x}|\le \frac{1}{2}$, $\inf _{V_x}|f_{0,x}|\ge 1$. There exists $x_1,x_2,\ldots ,x_m\in K$ such that $K\subset V_{x_1}\cup V_{x_2}\cup V_{x_m}\subset U_{x_1}\cup U_{x_1}\cup U_{x_2}\cup U_{x_m}$. Then $K\ni x\mapsto (F_{x_1},\cdots , F_{x_m})$ is an embedding. $\square$

#### Proof of Corollary 1.5

We proceed as in the proof of Corollary 1.5 by working on a compact coordinate patch *K* with coordinates $(x_1,\ldots ,x_{2n+1})$ and observing that in these coordinates a CR (*n*, 0)-form equals $fdz_1\wedge \ldots \wedge dz_n$ with *f* a CR function on *K*. $\square$

## Examples

We now consider Heisenberg group $\mathbb {H}=\mathbb {C}^n\times \mathbb {R}$ with CR structure

5.1 $$\begin{aligned} T^{(1,0)}\mathbb {H}:=\mathrm{span\,}\left\{ \frac{\partial }{\partial z_j} +i\frac{\partial \phi }{\partial z_j}(z)\frac{\partial }{\partial x_{2n+1}}\right\} _{j=1}^n, \end{aligned}$$

where $\phi \in \mathscr {C}^\infty (\mathbb {C}^n,\mathbb {R})$. Let $(\,\cdot \,|\,\cdot \,)_{\mathbb {H}}$ be the $L^2$ inner product on $\mathbb {H}$ induced by the Euclidean measure *dx* on ${\mathbb {R}}^{2n+1}$. Let

$$\begin{aligned} S_{\mathbb {H}}: L^2(\mathbb {H})\rightarrow \Big \{u\in L^2(\mathbb {H});\, \Big (\frac{\partial }{\partial {\overline{z}}_j}-i\frac{\partial \phi }{\partial {\overline{z}}_j}(z) \frac{\partial }{\partial x_{2n+1}}\Big )u=0\Big \} \end{aligned}$$

be the orthogonal projection with respect to $(\,\cdot \,|\,\cdot \,)_{\mathbb {H}}$ and let $S_{\mathbb {H}}(x,y)\in {\mathscr {D}}'(\mathbb {H}\times \mathbb {H})$ be the distribution kernel of $S_{\mathbb {H}}$. From Theorem 1.2, we deduce

### Corollary 5.1

With the notations used above, assume that $\left( \frac{\partial ^2\phi (z)}{\partial z_j\partial {\overline{z}}_k}\right) ^n_{j,k=1}$ is positive definite, at every $z\in {\mathbb {C}}^n$. Let $0<\lambda _1(z)\le \ldots \le \lambda _n(z)$ be the eigenvalues of $\left( \frac{\partial ^2\phi (z)}{\partial z_j\partial {\overline{z}}_k}\right) ^n_{j,k=1}$, for every $z\in {\mathbb {C}}^n$. Suppose that there is $C>0$ such that

5.2 $$\begin{aligned} \begin{aligned}&\sqrt{-1}\partial \overline{\partial }\left( -\log \det \left( \frac{\partial ^2\phi }{\partial z_j\partial {\overline{z}}_k}\right) ^n_{j,k=1}\right) \ge -C\sqrt{-1}\partial \overline{\partial }\phi \ \ ,\\&\frac{1}{\lambda _1(z)}\le C,\ \ \text {for every}\, z\in {\mathbb {C}}^n. \end{aligned} \end{aligned}$$

Let $D\Subset \mathbb {H}$ be any open set. Then,

5.3 $$\begin{aligned} S_{\mathbb {H}}(x,y)\equiv \int ^\infty _0e^{i\varphi (x,y)t}s(x,y,t)dt\ \ \text {on}\, D, \end{aligned}$$

where $\varphi \in \mathscr {C}^\infty (D\times D)$ and $s(x, y, t)\in S^{n}_{\mathrm{cl\,}}\big (D\times D\times {\mathbb {R}}_+\big )$ are as in Theorem 1.2.

### Example 5.2

With the notations used in Corollary 5.1, assume that

5.4 $$\begin{aligned} \phi (z)=|z|^2+r(z), \end{aligned}$$

with $r(z)\in \mathscr {C}^\infty _c(\mathbb {C}^n)$ and $\sqrt{-1}\partial \overline{\partial }(|z|^2+r(z))>0$ on $\mathbb {C}^n$. With this $\phi$, we can check the conditions of Corollary 5.1 fulfilled as follows. In fact, in this case, we have

$$\begin{aligned}\det \left( \frac{\partial ^2\phi }{\partial z_j\partial {\overline{z}}_k}\right) ^n_{j,k=1} =\det \left( \frac{\partial ^2(|z|^2+r(z))}{\partial z_j\partial {\overline{z}}_k}\right) ^n_{j,k=1}=1+F(z)>0 \end{aligned}$$

with some $F(z)\in \mathscr {C}^\infty _c(\mathbb {C}^n)$. And we have

$$\begin{aligned} \sqrt{-1}\partial \overline{\partial }\left( -\log \det \left( \frac{\partial ^2\phi }{\partial z_j\partial {\overline{z}}_k}\right) ^n_{j,k=1}\right) =\sqrt{-1}\partial \overline{\partial }\left( -\log (1+F(z))\right) \in \Omega _c^{1,1}(\mathbb {C}^n). \end{aligned}$$

Since $r(z)\in \mathscr {C}^\infty _c(\mathbb {C}^n)$ and $\sqrt{-1}\partial \overline{\partial }\phi =\sqrt{-1}\partial \overline{\partial }(|z|^2+r(z))>0$, we have a uniform lower bound for the smallest eigenvalue, i.e., $\lambda _1(z)>1/C_1$ for some $C_1>0$. Moreover, we can choose $C_2>0$ sufficiently large such that

$$\begin{aligned} \sqrt{-1}\partial \overline{\partial }\left( -\log (1+F(z))\right) +C_2\sqrt{-1}\partial \overline{\partial }\phi \ge 0, \end{aligned}$$

since the first term $\sqrt{-1}\partial \overline{\partial }\left( -\log (1+F(z))\right)$ is a real (1, 1)-form with compact support in $\mathbb {C}^n$ and the second term $\sqrt{-1}\partial \overline{\partial }\phi$ is a real positive (1, 1)-form with a uniformly positive lower bound for the smallest eigenvalue $\lambda _1(z)>1/C_1$ on $\mathbb {C}^n$. Finally, we obtain $C:=\max \{C_1,C_2\}>0$ as desired in (5.2).

With this $\phi$, it is easy to see that (5.2) hold. This example shows that, after small perturbation of the Levi form of Heisenberg group, we still can obtain the Szegő kernel expansion via Corollary 5.1.

### Example 5.3

Let $(X,T^{(1,0)}X)$ be a strictly pseudoconvex, CR manifold of dimension $2n+1$, $n\ge 1$, with a discrete, proper, CR action $\Gamma$ such that the quotient $X/\Gamma$ is compact. Assume *X* admits a transversal CR $\mathbb {R}$-action on *X* and let $\Theta _X$ be a $\Gamma$-invariant, $\mathbb {R}$-invariant, Hermitian metric on *X*. Then the conclusion of Theorem 1.2 holds. In fact, the $\Gamma$-covering manifold is complete and we can find the desired constant *C* depending on the fundamental domain $U\Subset X$ given by the $\Gamma$-action such that (1.7) is fulfilled. As a consequence, if we consider the circle bundle case in which $R^L=2\mathscr {L}$, we could obtain the Bergman kernel expansion for covering manifold [23, 6.1.2].
